# Palladium-catalyzed synthesis and anti-AD biological activity evaluation of *N*-aryl-debenzeyldonepezil analogues

**DOI:** 10.3389/fchem.2023.1282978

**Published:** 2023-12-08

**Authors:** Jing-Jing Xu, Jiao Luo, Heng Xi, Jin-Bu Xu, Lin-Xi Wan

**Affiliations:** ^1^ Department of Pharmacy, The Third People’s Hospital of Chengdu, Chengdu, China; ^2^ Sichuan Engineering Research Center for Biomimetic Synthesis of Natural Drugs, School of Life Science and Engineering, Southwest Jiaotong University, Chengdu, China; ^3^ Sichuan Research Center for Drug Precision Industrial Technology, West China School of Pharmacy, Sichuan University, Chengdu, China

**Keywords:** donepezil, Alzheimer’s disease, Buchwald-Hartwig reaction, AChE, neuroprotection

## Abstract

A series of novel *N*-aryl-debenzeyldonepezil derivatives (**1–26**) were designed and synthesized as cholinesterase inhibitors by the modification of anti-Alzheimer’s disease drug donepezil, using Palladium catalyzed Buchwald-Hartwig cross-coupling reaction as a key chemical synthesis strategy. *In vitro* cholinesterase inhibition studies demonstrated that the majority of synthesized compounds exhibited high selective inhibition of AChE. Among them, analogue **13** possessing a quinoline functional group showed the most potent AChE inhibition effect and significant neuroprotective effect against H_2_O_2_-induced injury in SH-SY5Y cells. Furthermore, Compound **13** did not show significant cytotoxicity on SH-SY5Y. These results suggest that **13** is a potential multifunctional active molecule for treating Alzheimer’s disease.

## Graphical Abstract

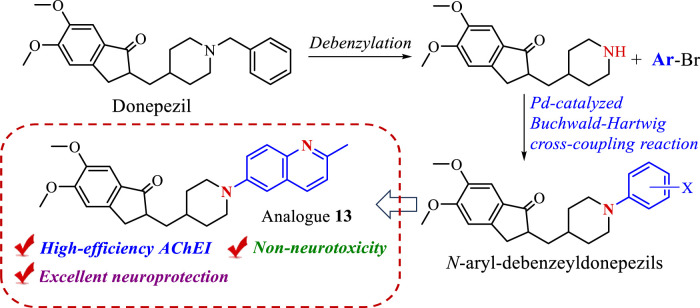

## 1 Introduction

**FIGURE 1:**
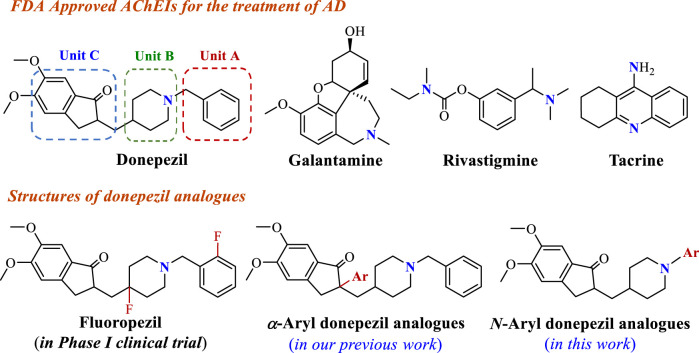
Approved AChEIs for the treatment of AD and structures of donepezil analogues.

Alzheimer’s disease (AD) is a common age-related neurodegenerative brain disorder characterized by progressive cognitive dysfunction, memory loss, and restrictions in daily activities (Ross and Poirier, 2004). According to the World Health Organization (WHO), approximately 50 million people suffer from AD disease around the world, and the number of patients will reach more than 150 million in 2050 as the population ages (Du et al., 2022). As one of the neurodegenerative disorders, the exact pathogenesis of AD is not fully known. Many studies have revealed that AD might involve abnormality of multiple neurochemical factors, such as low levels of acetylcholine (ACh), the amplitude of unusual deposits of *β*-amyloid (A*β*) peptide, hyperphosphorylated tau protein, neuronal apoptosis, and so on (Li et al., 2020; Perry et al., 2002; Lee et al., 2018; Mozaffarnia et al., 2020). Many hypotheses have been proposed based on these pathogenic factors to treat AD. The typical cholinergic hypothesis suggests that the selective loss of cholinergic neurons in the brain results in cognitive impairment in AD, whereas improvement of the central cholinergic transmission is an effective treatment strategy (Du et al., 2018). Among the several approaches attempted to enhance ACh levels in the brain, acetylcholinesterase inhibitors (AChEIs) have been proven in patients with mild or moderate AD (Anand and Singh, 2013). Up to now, donepezil, galantamine, rivastigmine, and tacrine as AChEIs have been approved by FDA used in AD treatment (Figure 1) (Pardo-Moreno et al., 2022). Although tacrine regrettably had to be withdrawn from the market in 2005 due to its hepatotoxicity (Summers et al., 1989), all four approved AChEIs possess a basic nitrogen atom. Notably, the basic nitrogen is protonated at physiological pH to provide a temporary positive charge and interacts with the active site of AChE, generating an impact on the overall affinity of AChE for the ligands (Peauger et al., 2017). Therefore, the incorporation of a nitrogen atom in bioactive compounds to recreate their unique properties has been regarded as an effective AChEIs medicinal development route.

Donepezil, approved by the FDA in 1996, is a second-generation specific reversible AChEI for the treatment of mild and moderate AD, showing good patient tolerance and mild adverse effects (Seltzer, 2007). The crystal structure of donepezil-*rh*AChE complex showed benzyl moiety (Unit A) in donepezil as an important site forms π-π interactions with residues of the active site (Akhoon et al., 2020). Furthermore, the piperidine ring (Unit B) and indanone (Unit C) reach out to π-π stacking interactions with peripheral anionic and catalytic active sites of AChE (Cheung et al., 2012). These findings contribute to the cognition of potential pharmacophores interacting with the residues of the active site. With increased research enthusiasm in terms of functional AChEI molecule design, using donepezil’s key structural moieties to develop novel AChEIs has drawn immense attention. For instance, fluorine-containing donepezil analogues fluoropezil possessing a longer drug-target residence time has been developed, which is preparing to enter phase II trial (Zhou et al., 2021; Qian et al., 2023). In our previous study, various nitrogen-containing functional groups were introduced into Unit C to provide a series of *α*-aryl donepezil analogues owning high AChE inhibition (Wan et al., 2021a). In order to find better anti-AD compounds, considering that the aromatic ring in Unit A is a vital binding site, we envisaged using aromatic nitrogen heterocycles in place of the phenyl group might form the additional bonding force to strengthen the AChE inhibitory activity. Furthermore, the Palladium-catalyzed Buchwald-Hartwig cross-coupling reaction is one of the powerful approaches for constructing abundant structures libraries via the formation of C-*N* bonds and provides an important chemical ligation strategy for the present study (Dorel et al., 2019).

Therefore, as part of our ongoing research program toward exploiting novel anti-AD bioactive molecules (Wan et al., 2021a; Wan et al., 2021b; Wan et al., 2021c; Miao et al., 2021; Xu et al., 2023; Zhang et al., 2023), the present work pay attention to the AChE inhibitory activity affected by the benzyl moiety in donepezil, and proposed the structural modification of donepezil for efficiently introducing aromatic nitrogen heterocycles or aryl functional groups located at *N* position via Palladium-catalyzed Buchwald-Hartwig reaction, hoping to find the potential bioactive molecules. Herein, the synthesis and pharmacological evaluation of donepezil analogues are reported.

## 2 Results and discussion

### 2.1 Chemistry

**SCHEME 1:**
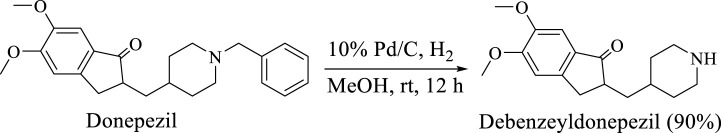
Preparation of debenzyldonepezil.

To conduct the biological activity evaluation of a new class of *N*-aryl-debenzeyldonepezil derivates, a set of target compound **1–26** were prepared by employing debenzeyldonepezil as a key intermediate and introducing an aryl functional group attached to the nitrogens of piperidine ring via Buchwald-Hartwig C-*N* cross-coupling. At the beginning of this study, donepezil was reacted with 10% Pd/C in methanol under a hydrogen atmosphere at room temperature for 4 h resulting in the generation of debenzeyldonepezil in 90% yield (Scheme 1). Next, debenzeyldonepezil and 2-bromopyridine were chosen as model substrates to explore the optimum Buchwald-Hartwig reaction conditions for the preparation of aiming *N*-aryl-debenzeyldonepezil. According to previous reports (Heravi et al., 2018; Dorel et al., 2019), after examination of ligands, bases, and solvents, we found that the ligand played a vital role in this C-*N* cross-coupling process. As shown in Table 1, the monophosphine ligands PPh_3_ and PCy_3_ afforded little product **1** (entries 1 and 2). The yield of the derived product was dramatically improved when using Mephos as a ligand. Therefore, the *N*-arylated product **1** could be recieved in as high as 89% yield in the presence of Pd(OAc)_2_ (10 mol%), Mephos (20 mol%), and *t*-BuOK (3 equiv.) in 1,4-dioxane at 90°C for 17 h (entry 3). Then, the influence of different bases and solvents on the reaction process was investigated. When other bases such as *t*-BuONa, K_2_CO_3_, and KF were used instead of *t*-BuOK, the desired cross-coupling product **1** was obtained in unsatisfactory yields (entries 4–6). Similarly, replacing 1,4-dioxane with DME and THF provided a lower yield of 37% and 23%, respectively (entries 7 and 8).

**TABLE 1:** Optimization of the *N*-arylation reaction conditions^a^.

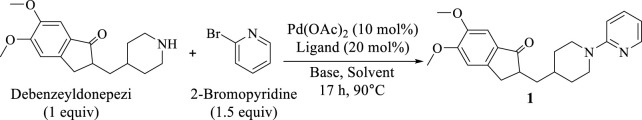				
Entry	Ligand	Base	Solvent	Yield (%)^b^
1	PPh_3_	*t*-BuOK	Dioxane	<5
2	PCy_3_	*t*-BuOK	Dioxane	<5
**3**	**Mephos**	***t-*BuOK**	**Dioxane**	**89**
4	Mephos	*t*-BuONa	Dioxane	10
5	Mephos	K_2_CO_3_	Dioxane	<5
6	Mephos	KF	Dioxane	<5
7	Mephos	*t*-BuOK	DME	37
8	Mephos	*t*-BuOK	THF	23

^a^ Reaction conditions: debenzeyldonepezil (0.1 mmol, 1.0 equiv.), 3-bromopyridine (0.15 mmol, 1.5 equiv.), Pd(OAc)_2_ (10 mol%), ligand (20 mol%), and base (0.3 mmol, 3 equiv.) in 2 mL solvent at 90°C for 17 h under argon atmosphere.

^b^ Isolated yield.

Bold values indicates the best reaction condition.

**SCHEME 2:**
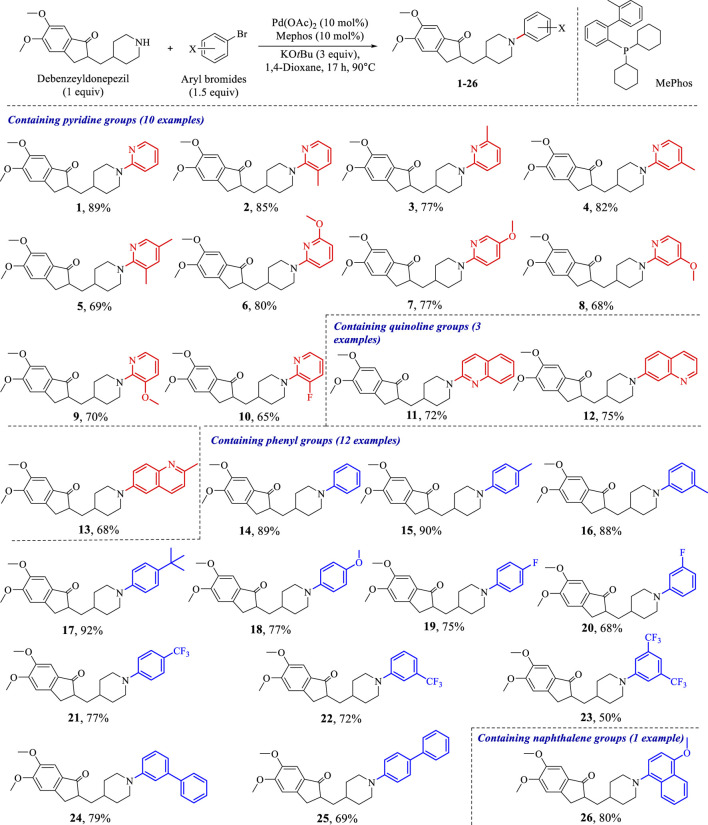
Palladium-catalyzed synthesis of *N*-aryl-debenzeyldonepezil derivatives.

Subsequently, Palladium-catalyzed Buchwald-Hartwig reactions between debenzeyldonepezil and various aryl bromides were conducted under standard reaction conditions. As exemplified in Scheme 2, the investigated *N*-heteroaryl bromides smoothly provided the corresponding *N*-arylated debenzeyldonepezil products **1–13** in excellent yields (65%–89%). Both electron-donating groups (methyl and methoxy) and halogen fluorine at *meta*-, *para*- or *ortho*-position of 2-bromopyridine were well tolerated. The bromide substrates bearing a multisubstituted ring were tested and afforded the expected products **3** and **5** in 77% and 69% yields, respectively. Meanwhile, quinoline groups were suitable as well in this process to furnish corresponding products in yields of 72% (**11**), 75% (**12**), and 68% (**13**). In addition to pyridine and quinoline bromides, the substrates carrying phenyl rings were examined and provided the conjugate products in 50–92% yield (**14**–**26**). The unsubstituted benzene-bromide furnished the derivative **14** in 89% yield. Similarly, the phenyl bromides bearing electron-donating groups (methyl, methoxy, and tertiary butyl) (**15**–**18**) worked efficiently. The substrates carrying electron-withdrawing groups (fluorine and trifluoromethyl) (**19**–**23**) were in lower yields in comparison to that of **15**–**18**. Compounds **24** and **25** equipped biphenyl groups were successfully prepared in yields of 79% and 69%, respectively. Furthermore, the reaction of debenzeyldonepezil with 1-bromo-4-methoxynaphthalene gave compound **26** an 80% yield. Structures of these *N*-aryl-debenzeyldonepezil analogues were characterized by NMR and HRESIMS spectra.

### 2.2 Biological activities

#### 2.2.1 *In vitro* cholinesterase inhibition studies

The inhibitory activity of *N*-aryl-debenzeyldonepezil analogues (**1–26**) against AChE (from electric eel) was initially evaluated by means of Ellman’s spectrophotometric method (Ellman et al., 1961), employing donepezil as the positive control. The screening test was conducted at the concentrations of 100 and 50 μM, and the ability of these molecules to inhibit the enzymes is reported in Table 2. When introducing methylpyridine-provided compounds **2–5**, the AChE inhibition activity of them was obviously lower than that of pyridine-substituted analogue **1**. For the position of the methoxyl substituent group, compounds **8** with 4-methoxy pyridine and **9** with 3-methoxypyridine obtained maintenance in activity, while the activity of **6** (6-methoxypyridine) and **7** (5-methoxypyridine) decreased. The only compound **10** (*Ee*AChE inhibitory rate = 44.1 ± 3.0% at 50 μM) in the series bearing a strong electron-withdrawing group also resulted in a larger decrease of AChE inhibitory activity than analogue **1**. The above results implied that the steric effects of these substituent groups possibly combining with electronic effects led to an adverse impact on the inhibition potency for this set of analogues. In short, the *N*-aryl-debenzeyldonepezils **1–10** equipping pyridine ring showed slightly decreased AChE inhibitory capacity, compared to that of donepezil, which revealed that the introduction of pyridine is at a significant disadvantage.

**TABLE 2:** Cholinesterase Inhibitory Activities of *N*-aryl-debenzeyldonepezil analogues.

Compound	*Ee*AChE inhibitory rate (IR) [%]^a^		*Eq*BuChE inhibitory rate (IR) [%]^a^	
	[I] = 100 *µ*M	[I] = 50 *µ*M	[I] = 100 *µ*M	[I] = 50 *µ*M
**1**	86.3 ± 3.0	58.3 ± 2.8	40.0 ± 2.1	19.4 ± 2.7
**2**	82.7 ± 2.8	52.1 ± 0.9	31.0 ± 3.3	−19.6 ± 1.3
**3**	82.2 ± 3.2	52.9 ± 3.2	37.9 ± 2.7	31.3 ± 1.0
**4**	84.4 ± 1.5	39.0 ± 2.7	33.5 ± 2.7	36.1 ± 2.4
**5**	84.2 ± 2.6	49.2 ± 3.2	29.0 ± 2.2	26.5 ± 3.7
**6**	87.4 ± 4.0	42.9 ± 2.0	0.9 ± 1.5	27.9 ± 1.3
**7**	86.4 ± 1.4	49.3 ± 1.9	13.1 ± 0.6	25.9 ± 2.4
**8**	83.1 ± 2.8	59.4 ± 1.0	11.7 ± 1.7	29.4 ± 3.7
**9**	82.3 ± 3.0	59.8 ± 1.1	9.4 ± 2.0	37.2 ± 0.9
**10**	77.7 ± 1.9	44.1 ± 3.0	6.8 ± 3.9	24.1 ± 2.3
**11**	87.3 ± 1.7	61.1 ± 2.1	24.6 ± 2.0	13.7 ± 2.8
**12**	84.5 ± 1.2	59.0 ± 1.7	16.9 ± 3.7	30.4 ± 1.7
**13**	89.0 ± 2.2	74.4 ± 3.5	40.3 ± 2.3	12.7 ± 2.0
**14**	81.1 ± 2.8	42.8 ± 3.7	13.3 ± 1.0	23.8 ± 2.2
**15**	78.5 ± 1.1	47.8 ± 2.8	−26.7 ± 1.8	35.8 ± 2.7
**16**	74.9 ± 2.0	52.9 ± 1.9	17.9 ± 2.1	2.1 ± 3.2
**17**	75.0 ± 3.1	53.7 ± 1.2	14.4 ± 3.2	4.5 ± 2.0
**18**	65.5 ± 2.1	53.0 ± 1.1	25.3 ± 2.2	5.1 ± 2.3
**19**	64.1 ± 3.2	61.9 ± 3.5	20.9 ± 2.9	11.9 ± 2.5
**20**	71.6 ± 3.9	57.1 ± 2.9	68.8 ± 1.0	1.1 ± 1.9
**21**	65.6 ± 1.0	56.9 ± 1.5	1.7 ± 3.6	1.3 ± 2.7
**22**	53.0 ± 0.9	59.3 ± 0.9	14.6 ± 2.9	−5.4 ± 3.2
**23**	60.3 ± 1.5	62.4 ± 2.0	3.5 ± 1.6	−18.8 ± 3.9
**24**	81.5 ± 2.9	50.4 ± 3.9	14.1 ± 1.9	1.2 ± 1.9
**25**	78.6 ± 1.0	50.7 ± 0.8	−3.8 ± 2.7	0.4 ± 2.5
**26**	68.1 ± 3.0	58.0 ± 2.3	18.8 ± 1.5	−4.9 ± 1.0
**Donepezil**	87.2 ± 1.2	66.9 ± 2.9	27.8 ± 2.8	25.3 ± 1.3

^a^ Percent inhibition data are the mean ± SD, of three independent experiments each performed in duplicate.

On the other hand, for the analogues possessing the quinoline fragments, compounds **11–13** exhibited favorable inhibitory activities, implying that the presence of this basic atomic group could increase interactions with the active sites of the enzymes. Compound **13** showed the best inhibitory potency, which needs an in-depth investigation. Many studies of AChE crystal structures have confirmed that the active site gorge is located in the deep and narrow “canyon” (Cheung et al., 2012; Liu et al., 2022), which implied that the larger group 2-methylquinoline might contribute to occupying the active site and obtaining compound **13** with high activity against AChE. Moreover, most compounds of the phenyl series (**14–23**) showed moderate inhibition effect against AChE. Introducing electron-withdrawing groups into the benzene ring, as shown in compounds **19–23**, improved activity more than that of analogues with electron-donating groups (**15–18**), probably due to electronic effects. However, two diphenyl analogues (**24** and **25**) among this subset of compounds seem to be noneffective, showing an *Ee*AChE inhibitory rate of only about 50% at 50 µM. Then, the *Eq*BuChE inhibition effects of these *N*-aryl-debenzeyldonepezil derivatives were also explored. As expected, all of them showed low inhibitory activity against *eq*BuChE.

#### 2.2.2 Molecular modelling studies

**FIGURE 2:**
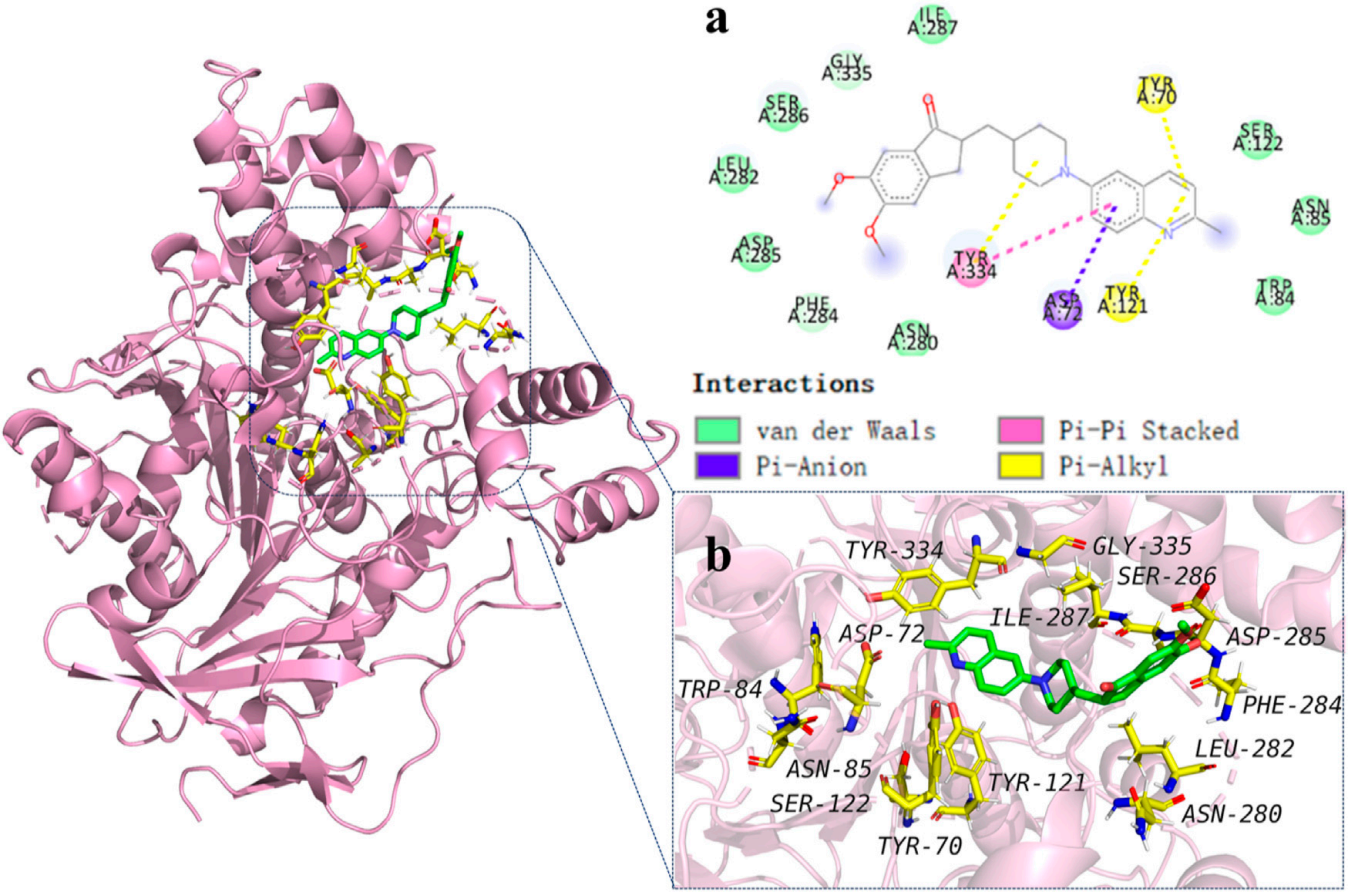
Molecular modeling of **13** in the active cavity of AChE (PDB ID: 1acj). **(A)** Residues of the active site involved in docking, and types of bonds involved in docking: π–π bond is shown as pick dashed lines, π–Alkyl bonds are shown as yellow dashed lines, and π–Anion bonds are shown as purple dashed lines; **(B)** Best docking positioning of compound **13** with AChE.

**FIGURE 3:**
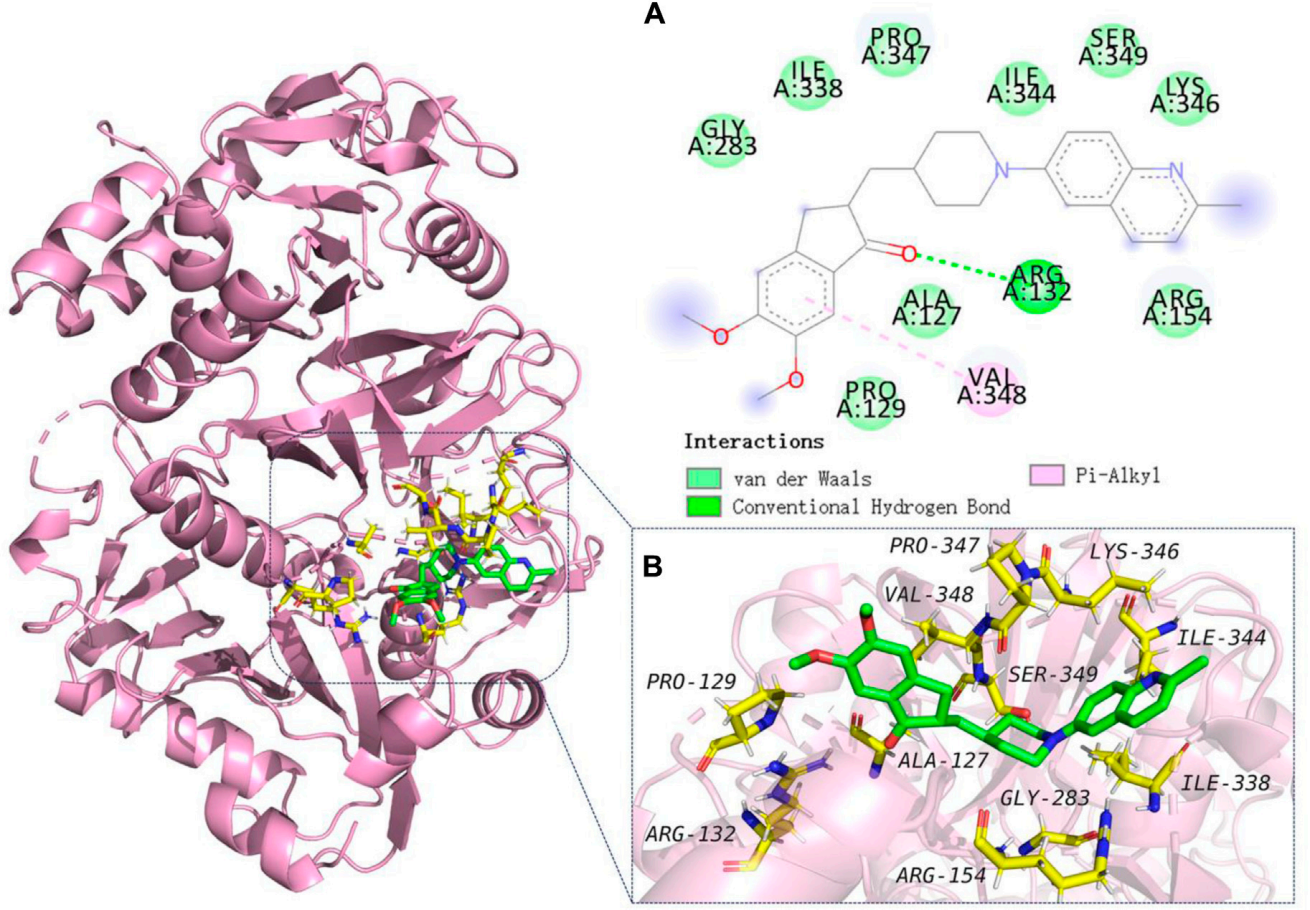
Molecular modeling of **13** in the active cavity of BuChE (PDB ID: 6ASM). **(A)** Residues of the active site involved in docking, and types of bonds involved in docking: π–π bond is shown as a pink dashed line and conventional hydrogen bonds are shown as green dashed lines; **(B)** Best docking positioning of compound **13** with BuChE.

To explore the binding mechanisms of compound **13** on AChE (PDB ID code: 1acj), a docking study to reveal the sites of binding and relative binging energy was performed. The highest-scored pose of **13** with a binding energy of −6.9 kcal/mol is depicted in Figure 2. Molecular docking results indicated that analogue **13** was suitably filled in the active site of AChE. Two π-alkyl hydrophobic interactions were formed by the quinoline fragment with Tyr 70 and Tyr 121, respectively. The benzene ring from quinoline established a π-anion bond with Asp 72. Moreover, attempt 334 was in a favorable position of stacking with the piperidine ring and quinoline’s benzene ring, forming a π–π and a π–alkyl interactions. Comparatively, in the active cavity of BuChE (PDB ID code: 6ASM), only the benzene ring from indanone interacted with Val 348 by π–alkyl interaction, and the carbonyl showed a conventional hydrogen bond with residues Arg 132. The highest-scored pose of **13** presented a binding energy of −5.6 kcal/mol (Figure 3). The above results could explain the compound **13** possessed the selective AChE inhibitory activity, which verified the *in vitro* enzyme inhibitory assay.

#### 2.2.3 Neuroprotection effects against H_2_O_2_-induced injury in SH-SY5Y cells

The death of neuronal cells is the central abnormality occurring in brains suffering from AD (Niikura et al., 2002). Neuroprotection is a useful way to protect the neuronal cells from damage, which potentially slows down or prevents the progression of AD (Coleman et al., 2004). To explore the therapeutic potential of synthesized analogues **1–26**, the neuroprotective effects of **1–26** against H_2_O_2_-induced injury in SH-SY5Y cells were performed. Firstly, the neurotoxicity of compounds **1–26** on SH-SY5Y cell viability was determined using the MTT assay method. As shown in Table 3, *N*-aryl-debenzeyldonepezils **6–8**, **17–22,** and **26** did not affect the cell viability of SH-SY5Y cells even at concentrations up to 50 μM, indicating that they were not neurotoxic. Then, non-cytotoxic compounds (**6–8**, **17–22,** and **26**) were examined for their potential neuroprotective activity on damaged SH-SY5Y cellular models induced by 700 μM H_2_O_2_. In the model group, 700 μM H_2_O_2_ significantly reduced cell viability and decreased to 61% compared to the control group. The protective effect of analogues **6–8**, **17–22,** and **26** against H_2_O_2_ are shown in Table 4. The survival rate of damaged SH-SY5Y cells increased to 115% after the following treatment of compound **13**, suggesting that synthetic compound 13 can protect cells against H_2_O_2_-induced cell death.

**TABLE 3:** Toxicity of compounds **1–26** on SH-SY5Y cells at 50 μM.

Compound	Cell viability (%)^a^	Compound	Cell viability (%)^a^
**1**	47.1 ± 1.1	**14**	112.0 ± 2.3
**2**	83.3 ± 1.1	**15**	101.8 ± 2.9
**3**	77.6 ± 4.7	**16**	81.1 ± 3.2
**4**	74.4 ± 1.4	**17**	103.1 ± 1.9
**5**	66.1 ± 0.5	**18**	109.6 ± 3.1
**6**	98.6 ± 1.5	**19**	99.6 ± 1.7
**7**	103.8 ± 2.9	**20**	100.2 ± 0.9
**8**	109.2 ± 2.2	**21**	96.5 ± 1.2
**9**	76.8 ± 2.5	**22**	91.5 ± 0.3
**10**	101.0 ± 2.6	**23**	56.8 ± 4.0
**11**	91.2 ± 4.9	**24**	85.1 ± 1.9
**12**	77.2 ± 3.0	**25**	66.6 ± 1.3
**13**	112.1 ± 1.9	**26**	103.3 ± 2.2

^a^ Data are the means ± SD, of at least three determinations.

**TABLE 4:** Neuroprotective effect of non-cytotoxic compounds against H_2_O_2_-induced injury in SH-SY5Y cells^a^.

Compound	Cell viability (%)^b^	Compound	Cell viability (%)^b^
**6**	44.6 ± 2.0	**17**	68.4 ± 2.3
**7**	45.5 ± 1.2	**18**	56.0 ± 1.5
**8**	43.3 ± 4.0	**19**	44.3 ± 3.9
**10**	41.3 ± 2.5	**20**	37.0 ± 2.2
**11**	56.0 ± 3.5	**21**	42.5 ± 1.9
**13**	115.3 ± 2.7	**22**	45.9 ± 2.6
**14**	52.6 ± 3.0	**26**	41.3 ± 1.7
**15**	49.3 ± 2.0		

^a^ Neuroprotective effect of compounds (50 μM) on H_2_O_2_-induced (700 μM) neurotoxicity in SH-SY5Y, cells. The cell viability in the control was taken as 100%, and the average value of cell viability under H_2_O_2_ exposure in the model group was 61.2% ± 1.3% (*n* = 3).

^b^ Data are the means ± SD, of at least three determinations.

#### 2.2.4 BBB prediction

Penetrating the blood-brain barrier (BBB) is extremely significant for anti-AD medicines, so the BBB penetration of compounds **1–26** was predicted by using Calculate Molecular Properties (Discovery Studio 2020 Client). The predicted data are listed in Table 5. The level “0” shows that the Brain-Blood ratio is greater than 5:1, the value “1” expresses that the Brain-Blood ratio is between 1:1 and 5:1, and the value “2” expresses that the Brain-Blood ratio is between 0.3:1 and 1:1. Besides compound **11**, most of the synthesized compounds are BBB permeable.

**TABLE 5:** The BBB prediction of compounds by Discovery Studio 2020 Client.

Compound	Level	ADMET_BBB	Compound	Level	ADMET_BBB
**1**	1	0.142	**14**	1	0.163
**2**	1	0.459	**15**	1	0.163
**3**	1	0.459	**16**	1	0.373
**4**	1	0.396	**17**	1	0.676
**5**	0	1.259	**18**	0	1.109
**6**	0	0.722	**19**	0	1.146
**7**	1	0.556	**20**	1	0.530
**8**	1	0.643	**21**	0	0.811
**9**	1	0.329	**22**	0	0.740
**10**	1	0.163	**23**	0	0.740
**11**	2	−0.156	**24**	0	0.827
**12**	1	0.649	**25**	0	0.827
**13**	1	0.610	**26**	0	1.146
**Donepezil**	0	0.649			

## 3 Conclusion

In summary, a series of new *N*-aryl-debenzeyldonepezil analogues (**1–26**) were efficiently synthesized in modification of donepezil by using Palladium-catalyzed Suzuki-Miyaura cross-coupling reaction as a key synthetic method. The cholinesterase inhibition and neuroprotective effects of these analogues were evaluated to develop potential candidate molecules for the treatment of AD. Among them, compound **13** exhibited good selective inhibitory activities against AChE, which might be attributed to the introduction of nitrogen-containing aromatic heterocycle. Molecular modeling was performed to explain the possible interaction between compound **13** and AChE. The prediction of bioavailability disclosed that analogue **13** had a favorable capacity to pass through the blood-brain barrier (BBB). Furthermore, further study showed that active compound **13** displayed non-neurotoxicity and good neuroprotective potency against H_2_O_2_-induced injury in SH-SY5Y cells. These results showed that compound **13** could be developed as an efficient multifunctional potential active molecule for the treatment of AD.

## 4 Experimental section

### 4.1 Chemistry

#### 4.1.1 Materials and methods

All reactions were conducted under an inert atmosphere of dry argon. Anhydrous THF was purchased from Aladdin and used without further purification. Unless otherwise noted, the reagents and solvents used in this article were all commercially available analytical or chemical grades and used directly without any purification. Reactions were monitored by thin layer chromatography (TLC) on silica gel plates (GF 254) using UV light to visualize the course of the reactions. Silica gel H (Qingdao Sea Chemical Factory, Qingdao, PR China) was used for column chromatography. NMR spectra were recorded by using a Bruker AV 400 or 600 nuclear magnetic resonance instrument. Chemical shifts (*δ*) were recorded in ppm relative to TMS as the internal standard. HRESIMS spectra were recorded on a Waters Acquity UPLC/Q–TOF micro mass spectrometer.

#### 4.1.2 Synthesis of debenzeyldonepezil

Donepezil (2.635 mmol) was suspended in 40 mL of a MeOH solution. To the reaction was added 10% Pd/C (0.2635 mmol), stirred at 25°C under an argon atmosphere for 12 h. Then, the mixture was filtered and evaporated under a vacuum. The reaction crude product was purified by flash column chromatography using PE-EA (3:1 to 1:1) to obtain debenzeyldonepezil as amorphous powder (90% yield); ^1^H NMR (400 MHz, CDCl_3_) *δ* 7.16 (s, 1H), 6.85 (s, 1H), 3.95 (s, 3H), 3.89 (s, 3H), 3.23 (dd, *J* = 17.4, 8.0 Hz, 1H), 3.09 (t, *J* = 9.6 Hz, 2H), 2.74−2.67 (m, 2H), 2.63−2.57 (m, 2H), 1.92−1.86 (m, 1H), 1.77 (d, *J* = 13.2 Hz, 1H), 1.69 (d, *J* = 13.2 Hz, 1H), 1.69 (d, *J* = 13.2 Hz, 1H), 1.34−1.24 (m, 2H), 1.22−1.11 (m, 2H); ^13^C NMR (100 MHz, CDCl_3_) *δ* 208.0, 155.6, 149.6, 148.9, 129.4, 107.5, 104.5, 56.3, 56.2, 46.9, 46.8, 45.3, 39.5, 35.0, 34.4, 33.4, 33.1; HRESIMS *m/z* 290.1740 [M + H]^+^ (calcd for C_17_H_24_NO_3_, 290.1756).

#### 4.1.3 Synthesis of *N*-aryl-debenzeyldonepezil analogues 1–26

The suspension of Pd(OAc)_2_ (5 mol%) and Mephos (10 mol%) in anhydrous 1,4-dioxane (3 mL) was stirred at 30°C under an argon atmosphere for 2 h to obtain a brown solution. The brown solution was added to a 10 mL sealed dry reaction vial containing debenzeyldonepezil (1 mmol), naphthalenyl, bromides (1.5 mmol), and *t*-BuOK (3 mmol) via a syringe. The reaction mixture was stirred for 17 h at 90°C before quenching with two drops of H_2_O. Then, the mixture was diluted with ethyl acetate (3 mL), and filtered over a pad of MgSO_4_. The pad was rinsed with ethyl acetate, and the mixture was concentrated *in vacuo*. The reaction crude product was purified by flash column chromatography using PE-EA (2:1–1:2) to obtain the *N*-aryl-debenzeyldonepezil analogues **1–26** (50%–89% yields).

Compound **1**, amorphous powder, 89% yield; ^1^H NMR (400 MHz, CDCl_3_) *δ* 8.18−8.16 (m, 1H), 7.44−7.42 (m, 1H), 7.17 (s, 1H), 6.86 (s, 1H), 6.66 (d, *J* = 8.4 Hz, 1H), 6.57−6.55 (m, 1H), 4.33 – 4.28 (m, 2H), 3.96 (s, 3H), 3.90 (s, 3H), 3.31 (q, *J* = 8.4 Hz, 1H), 2.87−2.79 (m, 2H), 2.77−2.70 (m, 2H), 1.97−1.90 (m, 1H), 1.87−1.78 (m, 2H), 1.40−1.25 (m, 4H); ^13^C NMR (100 MHz, CDCl_3_) *δ* 207.8, 159.8, 155.7, 149.7, 148.9, 148.2, 137.6, 129.5, 112.9, 107.6, 107.4, 104.6, 56.4, 56.3, 45.9, 45.9, 45.5, 39.0, 35.0, 33.6, 32.8, 31.6; HRESIMS *m/z* 367.2012 [M + H]^+^ (calcd for C_22_H_27_N_2_O_3_, 367.2022).

Compound **2**, amorphous powder, 85% yield; ^1^H NMR (400 MHz, CDCl_3_) *δ* 8.15−8.14 (m, 1H), 7.39−7.37 (m, 1H), 7.18 (s, 1H), 6.87 (s, 1H), 6.82 (dd, *J* = 7.2, 4.8 Hz, 1H), 3.96 (s, 3H), 3.91 (s, 3H), 3.49−3.42 (m, 2H), 3.28 (q, *J* = 8.4 Hz, 1H), 2.83−2.73 (m, 4H), 2.26 (s, 3H), 2.02−1.96 (m, 1H), 1.91−1.77 (m, 2H), 1.53−1.36 (m, 4H); ^13^C NMR (100 MHz, CDCl_3_) *δ* 207.9, 162.6, 155.7, 149.6, 148.9, 145.3, 139.2, 129.5, 125.3, 117.7, 107.5, 104.6, 56.4, 56.2, 50.39, 50.38, 45.6, 39.0, 34.8, 33.7, 33.4, 32.3, 18.4; HRESIMS *m/z* 381.2162 [M + H]^+^ (calcd for C_23_H_29_N_2_O_3_, 381.2178).

Compound **3**, amorphous powder, 77% yield; ^1^H NMR (400 MHz, CDCl_3_) *δ* 7.34 (t, *J* = 8.4 Hz, 1H), 7.18 (s, 1H), 6.87 (s, 1H), 6.45 (d, *J* = 2.7 Hz, 1H), 6.43 (s, 1H), 4.35−4.30 (m, 2H), 3.96 (s, 3H), 3.91 (s, 3H), 3.29 (q, *J* = 8.0 Hz, 1H), 2.83−2.79 (m, 1H), 2.78−2.71 (m, 3H), 2.39 (s, 3H), 1.97−1.90 (m, 1H), 1.86−1.77 (m, 2H), 1.40−1.30 (m, 4H); ^13^C NMR (100 MHz, CDCl_3_) *δ* 207.8, 159.5, 156.9, 155.7, 149.6, 148.9, 137.8, 129.4, 112.2, 107.5, 104.6, 104.0, 56.3, 56.2, 46.0, 45.9, 45.4, 38.9, 35.0, 33.5, 32.8, 31.6, 24.7; HRESIMS *m/z* 381.2165 [M + H]^+^ (calcd for C_23_H_29_N_2_O_3_, 381.2178).

Compound **4**, amorphous powder, 82% yield; ^1^H NMR (400 MHz, CDCl_3_) *δ* 8.04 (d, *J* = 5.2 Hz, 1H), 7.18 (s, 1H), 6.87 (s, 1H), 6.48 (s, 1H), 6.43 (d, *J* = 5.2 Hz, 1H), 4.31−4.27 (m, 2H), 3.96 (s, 3H), 3.91 (s, 3H), 3.31 (q, *J* = 9.2 Hz, 1H), 2.85−2.79 (m, 2H), 2.78−2.70 (m, 2H), 2.25 (s, 3H), 1.97−1.90 (m, 1H), 1.87−1.77 (m, 2H), 1.40−1.29 (m, 4H); ^13^C NMR (100 MHz, CDCl_3_) *δ* 207.8, 160.0, 155.7, 149.6, 148.9, 148.4, 147.7, 129.4, 114.4, 107.8, 107.5, 104.6, 56.4, 56.3, 46.1, 46.0, 45.4, 39.0, 35.0, 33.5, 32.7, 31.6, 21.6; HRESIMS *m/z* 381.2163 [M + H]^+^ (calcd for C_23_H_29_N_2_O_3_, 381.2178).

Compound **5**, amorphous powder, 69% yield; ^1^H NMR (400 MHz, CDCl_3_) *δ* 7.96 (d, *J* = 2.0 Hz, 1H), 7.22 (d, *J* = 2.0 Hz, 1H), 7.19 (s, 1H), 6.88 (s, 1H), 3.97 (s, 3H), 3.91 (s, 3H), 3.40−3.35 (m, 2H), 3.29 (q, *J* = 8.0 Hz, 1H), 2.79−2.73 (m, 4H), 2.24 (s, 3H), 2.21 (s, 3H), 2.02−1.96 (m, 1H), 1.90−1.76 (m, 2H), 1.53−1.37 (m, 4H); ^13^C NMR (100 MHz, CDCl_3_) *δ* 208.0, 160.7, 155.6, 149.6, 149.0, 145.2, 140.2, 129.5, 127.0, 125.0, 107.5, 104.6, 56.4, 56.3, 50.8, 50.7, 45.7, 39.0, 34.8, 33.7, 33.4, 32.3, 18.1, 17.6; HRESIMS *m/z* 395.2321 [M + H]^+^ (calcd for C_24_H_31_N_2_O_3_, 395.2335).

Compound **6**, amorphous powder, 80% yield; ^1^H NMR (400 MHz, CDCl_3_) *δ* 7.37 (t, *J* = 8.0 Hz, 1H), 7.17 (s, 1H), 6.86 (s, 1H), 6.16 (d, *J* = 8.0 Hz, 1H), 6.02 (d, *J* = 8.0 Hz, 1H), 4.33−4.27 (m, 2H), 3.96 (s, 3H), 3.90 (s, 3H), 3.85 (s, 3H), 3.28 (q, *J* = 9.2 Hz, 1H), 2.83−2.71 (m, 4H), 1.96−1.89 (m, 1H), 1.85−1.76 (m, 2H), 1.40−1.24 (m, 4H); ^13^C NMR (100 MHz, CDCl_3_) *δ* 207.7, 163.2, 158.5, 155.6, 149.6, 148.8, 140.1, 129.4, 107.5, 104.5, 98.3, 97.4, 56.3, 56.2, 53.0, 45.8, 45.7, 45.4, 38.9, 34.9, 33.5, 32.6, 31.5; HRESIMS *m/z* 397.2116 [M + H]^+^ (calcd for C_23_H_29_N_2_O_4_, 397.2127).

Compound **7**, amorphous powder, 77% yield; ^1^H NMR (400 MHz, CDCl_3_) *δ* 7.93 (d, *J* = 3.2 Hz, 1H), 7.18 (s, 1H), 7.13 (dd, *J* = 6.0, 2.8 Hz, 1H), 6.87 (s, 1H), 6.66 (d, *J* = 8.8 Hz, 1H), 4.16−4.10 (m, 2H), 3.96 (s, 3H), 3.91 (s, 3H), 3.79 (s, 3H), 3.31 (q, *J* = 9.2 Hz, 1H), 2.81−2.77 (m, 2H), 2.76−2.71 (m, 2H), 1.97−1.91 (m, 1H), 1.88−1.78 (m, 2H), 1.44−1.30 (m, 4H); ^13^C NMR (100 MHz, CDCl_3_) *δ* 207.9, 155.7, 155.6, 149.7, 149.0, 148.9, 133.8, 129.5, 125.2, 108.7, 107.6, 104.6, 56.5, 56.4, 56.3, 47.2, 47.1, 45.5, 39.0, 34.9, 33.6, 32.8, 31.6; HRESIMS *m/z* 397.2110 [M + H]^+^ (calcd for C_23_H_29_N_2_O_4_, 397.2127).

Compound **8**, amorphous powder, 68% yield; ^1^H NMR (400 MHz, CDCl_3_) *δ* 8.03 (d, *J* = 5.8 Hz, 1H), 7.17 (s, 1H), 6.86 (s, 1H), 6.21 (dd, *J* = 5.8, 2.2 Hz, 1H), 6.12 (d, *J* = 2.2 Hz, 1H), 4.30−4.24 (m, 2H), 3.96 (s, 3H), 3.90 (s, 3H), 3.80 (s, 3H), 3.31 (q, *J* = 9.2 Hz, 1H), 2.86−2.78 (m, 2H), 2.75−2.71 (m, 2H), 1.96−1.90 (m, 1H), 1.86−1.77 (m, 2H), 1.40−1.29 (m, 4H); ^13^C NMR (100 MHz, CDCl_3_) *δ* 207.8, 167.4, 161.6, 155.7, 149.6, 149.2, 148.8, 129.4, 107.5, 104.6, 100.7, 92.1, 56.4, 56.2, 55.0, 46.1, 46.0, 45.4, 38.9, 35.0, 33.5, 32.7, 31.5; HRESIMS *m/z* 397.2117 [M + H]^+^ (calcd for C_23_H_29_N_2_O_4_, 397.2127).

Compound **9**, amorphous powder, 70% yield; ^1^H NMR (400 MHz, CDCl_3_) *δ* 7.86 (dd, *J* = 3.6, 1.2 Hz, 1H), 7.18 (s, 1H), 7.01 (dd, *J* = 6.4, 1.6 Hz, 1H), 6.87 (s, 1H), 6.81 (dd, *J* = 8.0, 4.8 Hz, 1H), 3.96 (s, 3H), 3.94−3.92 (m, 2H), 3.90 (s, 3H), 3.84 (s, 3H), 3.28 (q, *J* = 9.2 Hz, 1H), 2.80−2.72 (m, 4H), 2.01−1.94 (m, 1H), 1.88−1.76 (m, 2H), 1.57−1.34 (m, 4H); ^13^C NMR (100 MHz, CDCl_3_) *δ* 207.9, 155.6, 153.0, 149.6, 148.9, 147.2, 138.9, 129.5, 117.5, 116.8, 107.5, 104.6, 56.3, 56.2, 55.4, 49.1, 49.0, 45.6, 39.0, 35.0, 33.5, 33.3, 32.0; HRESIMS *m/z* 397.2114 [M + H]^+^ (calcd for C_23_H_29_N_2_O_4_, 397.2127).

Compound **10**, amorphous powder, 65% yield; ^1^H NMR (400 MHz, CDCl_3_) *δ* 7.97−7.94 (m, 1H), 7.24−7.10 (m, 2H), 6.85 (d, *J* = 2.0 Hz, 1H), 6.73−6.66 (m, 1H), 4.14−4.02 (m, 2H), 3.94 (s, 3H), 3.89 (s, 3H), 3.32−3.17 (m, 1H), 2.94−2.66 (m, 4H), 1.98−1.92 (m, 1H), 1.87−1.73 (m, 2H), 1.49−1.37 (m, 4H); ^13^C NMR (100 MHz, CDCl_3_) *δ* 208.0, 158.2, 149.6 (*d*, J = 30 Hz), 142.8 (d, *J* = 5 Hz), 142.6 (d, *J* = 60 Hz), 131.4, 129.4, 123.1 (d, *J* = 19 Hz), 115.8, 107.5, 104.5, 56.3, 56.2, 48.8, 48.7, 45.5, 39.1, 34.9, 33.5, 33.3, 31.9, 28.8; HRESIMS *m/z* 385.1911 [M + H]^+^ (calcd for C_22_H_26_FN_2_O_3_, 385.1927).

Compound **11**, amorphous powder, 72% yield; ^1^H NMR (400 MHz, CDCl_3_) *δ* 7.86 (d, *J* = 10.0 Hz, 1H), 7.69 (d, *J* = 8.4 Hz, 1H), 7.58 (dd, *J* = 6.4, 1.2 Hz, 1H), 7.51−7.49 (m, 1H), 7.20−7.20 (m, 1H), 7.18 (s, 1H), 7.01 (d, *J* = 9.2 Hz, 1H), 6.87 (s, 1H), 4.59−4.56 (m, 2H), 3.97 (s, 3H), 3.91 (s, 3H), 3.32−3.24 (q, *J* = 8.4 Hz, 1H), 3.00−2.93 (m, 2H), 2.79−2.72 (m, 2H), 1.99−1.92 (m, 1H), 1.90−1.83 (m, 2H), 1.42−1.31 (m, 4H); ^13^C NMR (100 MHz, CDCl_3_) *δ* 207.7, 157.7, 155.7, 149.7, 148.8, 137.4, 129.5, 129.4, 127.3, 127.2, 126.7, 123.0, 122.2, 110.0, 107.5, 104.6, 56.4, 56.3, 45.8, 45.4, 45.3, 38.9, 35.1, 33.6, 32.9, 31.8; HRESIMS *m/z* 417.2166 [M + H]^+^ (calcd for C_26_H_29_N_2_O_3_, 417.2178).

Compound **12**, amorphous powder, 75% yield; ^1^H NMR (400 MHz, CDCl_3_) *δ* 8.88 (dd, *J* = 4.2, 1.8 Hz, 1H), 8.50 (d, *J* = 7.6 Hz, 1H), 7.79 (d, *J* = 8.4 Hz, 1H), 7.61 (t, *J* = 7.6 Hz, 1H), 7.38 (dd, *J* = 8.6, 4.0 Hz, 1H), 7.20 (s, 1H), 7.12 (d, *J* = 6.4 Hz, 1H), 6.88 (s, 1H), 3.97 (s, 3H), 3.92 (s, 3H), 3.41−3.35 (m, 2H), 3.34−3.27 (m, 1H), 2.83−2.75 (m, 4H), 2.08−2.03 (m, 1H), 1.97−1.87 (m, 2H), 1.71−1.62 (m, 4H); ^13^C NMR (100 MHz, CDCl_3_) *δ* 207.8, 155.7, 150.9, 150.2, 149.7, 149.6, 148.9, 132.6, 129.5, 129.4, 124.4, 120.3, 115.2, 115.1, 107.5, 104.6, 56.4, 56.3, 54.2, 45.6, 39.0, 34.7, 33.8, 33.5, 32.6, 29.8; HRESIMS *m/z* 417.2161 [M + H]^+^ (calcd for C_26_H_29_N_2_O_3_, 417.2178).

Compound **13**, amorphous powder, 68% yield; ^1^H NMR (400 MHz, CDCl_3_) *δ* 7.89 (s, 1H), 7.87 (s, 1H), 7.48 (dd, *J* = 6.8, 2.4 Hz, 1H), 7.19−7.17 (m, 2H), 7.02 (d, *J* = 2.8 Hz, 1H), 6.87 (s, 1H), 3.96 (s, 3H), 3.91 (s, 3H), 3.84−3.78 (m, 2H), 3.32 (q, *J* = 7.6 Hz, 1H), 2.84−2.72 (m, 4H), 2.68 (s, 3H), 2.00−1.97 (m, 1H), 1.94−1.90 (m, 2H), 1.57−1.37 (m, 4H); ^13^C NMR (100 MHz, CDCl_3_) *δ* 207.8, 155.9, 155.7, 149.7, 149.4, 148.9, 143.3, 135.1, 129.4, 129.1, 127.7, 123.3, 122.3, 109.6, 107.5, 104.6, 56.4, 56.2, 50.3, 50.2, 45.4, 38.8, 34.5, 33.5, 32.9, 31.8, 25.0; HRESIMS *m/z* 431.2322 [M + H]^+^ (calcd for C_27_H_31_N_2_O_3_, 431.2335).

Compound **14**, amorphous powder, 89% yield; ^1^H NMR (400 MHz, CDCl_3_) *δ* 7.35−7.31 (m, 3H), 7.04 (d, *J* = 8.0 Hz, 2H), 6.95 (s, 1H), 6.90 (t, *J* = 7.6 Hz, 1H), 4.04 (s, 3H), 3.99 (s, 3H), 3.81−3.74 (m, 1H), 3.39−3.32 (m, 1H), 2.85−2.81 (m, 2H), 2.79−2.75 (m, 2H), 2.09−2.00 (m, 1H), 1.97−1.88 (m, 2H), 1.60−1.44 (m, 4H); ^13^C NMR (100 MHz, CDCl_3_) *δ* 207.8, 155.7, 152.0, 149.6, 148.8, 129.4, 129.2, 119.5, 116.7, 107.5, 104.6, 56.3, 56.2, 50.1, 50.0 45.4, 38.8, 34.5, 33.5, 33.0, 31.9; HRESIMS *m/z* 366.2050 [M + H]^+^ (calcd for C_23_H_28_NO_3_, 366.2069).

Compound **15**, amorphous powder, 90% yield; ^1^H NMR (400 MHz, CDCl_3_) *δ* 7.18 (s, 1H), 7.06 (d, *J* = 8.4 Hz, 2H), 6.86 (s, 1H), 6.87 (d, *J* = 8.4 Hz, 2H), 3.97 (s, 3H), 3.91 (s, 3H), 3.29 (q, *J* = 8.0 Hz, 1H), 2.78−2.62 (m, 4H), 2.26 (s, 3H), 1.99−1.93 (m, 1H), 1.89−1.79 (m, 2H), 1.50−1.42 (m, 2H), 1.53−1.34 (m, 4H); ^13^C NMR (100 MHz, CDCl_3_) *δ* 207.9, 155.7, 150.0, 149.7, 148.9, 129.8, 129.5, 129.1, 117.2, 107.6, 104.6, 56.4, 56.3, 50.9, 50.8, 45.6, 38.9, 34.6, 33.6, 33.2, 32.0, 20.5; HRESIMS *m/z* 380.2210 [M + H]^+^ (calcd for C_24_H_30_NO_3_, 380.2226).

Compound **16**, amorphous powder, 88% yield; ^1^H NMR (400 MHz, CDCl_3_) *δ* 7.18 (s, 1H), 7.14 (t, *J* = 7.6 Hz, 1H), 6.87 (s, 1H), 6.76 (d, *J* = 8.6 Hz, 2H), 6.66 (d, *J* = 7.6 Hz, 1H), 3.97 (s, 3H), 3.91 (s, 3H), 3.71−3.66 (m, 2H), 3.31 (q, *J* = 8.0 Hz, 1H), 2.78−2.67 (m, 4H), 2.31 (s, 3H), 1.99−1.94 (m, 1H), 1.89−1.79 (m, 2H), 1.52−1.34 (m, 4H); ^13^C NMR (100 MHz, CDCl_3_) *δ* 207.8, 155.7, 152.1, 149.6, 148.9, 138.9, 129.5, 129.1, 120.5, 117.7, 113.9, 107.6, 104.7, 56.4, 56.4, 56.3, 50.3, 45.5, 39.0, 34.6, 33.6, 33.2, 32.0, 22.0; HRESIMS *m/z* 380.2213 [M + H]^+^ (calcd for C_24_H_30_NO_3_, 380.2226).

Compound **17**, amorphous powder, 92% yield; ^1^H NMR (400 MHz, CDCl_3_) *δ* 7.29−7.26 (m, 2H), 7.19 (s, 1H), 6.91−6.89 (m, 2H), 6.87 (s, 1H), 3.97 (s, 3H), 3.91 (s, 3H), 3.69−3.63 (m, 2H), 3.31 (q, *J* = 8.0 Hz, 1H), 2.79−2.73 (m, 2H), 2.72−2.64 (m, 2H), 1.99−1.93 (m, 1H), 1.89−1.79 (m, 2H), 1.52−1.41 (m, 4H), 1.29 (s, 9H); ^13^C NMR (100 MHz, CDCl_3_) *δ* 207.8, 155.7, 149.6, 148.9, 142.2, 129.5, 126.0, 125.9, 116.4, 107.5, 104.6, 56.4, 56.3, 50.4, 50.3, 45.5, 38.9, 34.5, 34.1, 33.5, 33.2, 32.0, 31.6; HRESIMS *m/z* 422.2681 [M + H]^+^ (calcd for C_27_H_36_NO_3_, 422.2695).

Compound **18**, amorphous powder, 77% yield; ^1^H NMR (400 MHz, CDCl_3_) *δ* 7.18 (s, 1H), 6.94 (d, *J* = 9.2 Hz, 2H), 6.87 (s, 1H), 6.84 (d, *J* = 9.2 Hz, 2H), 3.97 (s, 3H), 3.91 (s, 3H), 3.77 (s, 3H), 3.55−3.50 (m, 2H), 3.26 (q, *J* = 8.0 Hz, 1H), 2.78−2.71 (m, 2H), 2.68−2.60 (m, 2H), 2.00−1.93 (m, 1H), 1.90−1.80 (m, 2H), 1.54−1.35 (m, 4H); ^13^C NMR (100 MHz, CDCl_3_) *δ* 207.9, 155.7, 153.9, 149.7, 148.9, 146.5, 129.5, 119.0, 114.6, 107.6, 104.7, 56.4, 56.3, 55.7, 51.8, 45.5, 38.9, 34.4, 33.5, 33.3, 32.1; HRESIMS *m/z* 396.2160 [M + H]^+^ (calcd for C_24_H_30_NO_4_, 396.2175).

Compound **19**, amorphous powder, 75% yield; ^1^H NMR (400 MHz, CDCl_3_) *δ* 7.18 (s, 1H), 6.98−6.95 (m, 1H), 6.94−6.90 (m, 2H), 6.90−6.86 (m, 2H), 3.97 (s, 3H), 3.91 (s, 3H), 3.59−3.54 (m, 2H), 3.27 (q, *J* = 9.2 Hz, 1H), 2.80−2.72 (m, 2H), 2.70−2.63 (m, 2H), 2.02−1.87 (m, 2H), 1.88−1.74 (m, 2H), 1.60 (s, 1H), 1.50 (m, 2H); ^13^C NMR (100 MHz, CDCl_3_) *δ* 207.8, 155.8, 149.7, 148.9, 148.8 (d, *J* = 2.4 Hz), 129.5, 118.6 (d, *J* = 10 Hz), 115.7 (d, *J* = 20 Hz), 107.6, 104.7, 56.4, 56.3, 51.3, 45.5, 38.9, 34.4, 33.6, 33.2, 32.1; HRESIMS *m/z* 384.1961 [M + H]^+^ (calcd for C_23_H_27_FNO_3_, 384.1975).

Compound **20**, amorphous powder, 68% yield; ^1^H NMR (400 MHz, CDCl_3_) *δ* 7.19 (d, *J* = 5.2 Hz, 1H), 7.15 (dd, *J* = 8.3, 7.1 Hz, 1H), 6.87 (s, 1H), 6.69 (dd, *J* = 6.0, 2.4 Hz, 1H), 6.61 (dt, *J* = 12.8, 2.4 Hz, 1H), 6.51−6.46 (m, 1H), 3.97 (s, 3H), 3.91 (s, 3H), 3.73−3.68 (m, 2H), 3.29 (q, *J* = 8.0 Hz, 1H), 2.78−2.71 (m, 4H), 1.98−1.91 (m, 1H), 1.89−1.79 (m, 2H), 1.60−1.36 (m, 4H); ^13^C NMR (100 MHz, CDCl_3_) *δ* 207.8, 164.1 (d, *J* =240 MHz), 155.8, 153.5 (d, *J* =10 MHz), 149.7, 148.9, 130.2 (d, *J* =10 MHz), 129.5, 111.7, 107.6, 105.5 (d, *J* =20 MHz), 104.7, 103.1 (d, *J* =20 MHz), 56.4, 56.3, 49.6, 49.6, 45.5, 38.9, 34.5, 33.6, 32.8, 31.7; HRESIMS *m/z* 384.1967 [M + H]^+^ (calcd for C_23_H_27_FNO_3_, 384.1975).

Compound **21**, amorphous powder, 77% yield; ^1^H NMR (400 MHz, CDCl_3_) *δ* 7.47 (d, *J* = 8.4 Hz, 2H), 7.18 (s, 1H), 6.94 (d, *J* = 8.4 Hz, 2H), 6.87 (s, 1H), 3.97 (s, 3H), 3.91 (s, 3H), 3.84−3.79 (m, 2H), 3.30 (q, *J* = 8.0, 1H), 2.86−2.81 (m, 2H), 2.80−2.71 (m, 2H), 1.98−1.91 (m, 1H), 1.90−1.69 (m, 2H), 1.60−1.35 (m, 4H); ^13^C NMR (100 MHz, CDCl_3_) *δ* 207.7, 155.8, 153.7, 149.8, 148.9, 129.5, 126.6 (q, *J* = 4 Hz), 114.9, 111.5, 107.6, 104.7, 100.6, 56.5, 56.3, 48.9, 48.9, 45.4, 38.9, 34.5, 33.6, 32.6, 31.6; HRESIMS *m/z* 434.1930 [M + H]^+^ (calcd for C_24_H_27_F_3_NO_3_, 434.1943).

Compound **22**, amorphous powder, 72% yield; ^1^H NMR (400 MHz, CDCl_3_) *δ* 7.32 (t, *J* = 8.0 Hz, 1H), 7.18 (s, 1H), 7.12 (t, *J* = 2.0 Hz, 1H), 7.08−7.01 (m, 2H), 6.87 (s, 1H), 3.96 (s, 3H), 3.91 (s, 3H), 3.76−3.70 (m, 2H), 3.27 (q, *J* = 7.6 Hz, 1H), 2.81−2.71 (m, 4H), 1.98−1.93 (m, 1H), 1.91−1.82 (m, 2H), 1.50−1.35 (m, 4H); ^13^C NMR (100 MHz, CDCl_3_) *δ* 207.7, 155.8, 152.0, 149.7, 148.9, 131.5 (q, *J* = 30 MHz), 129.7, 129.5, 124.6 (q, *J* = 280 MHz) 119.4, 115.5 (q, *J* = 10 MHz), 112.6 (q, *J* = 10 MHz), 107.6, 104.6, 56.4, 56.3, 49.7, 49.6, 45.4, 38.8, 34.4, 33.6, 32.8, 31.7; HRESIMS *m/z* 434.1935 [M + H]^+^ (calcd for C_24_H_27_F_3_NO_3_, 434.1943).

Compound **23**, amorphous powder, 50% yield; ^1^H NMR (400 MHz, CDCl_3_) *δ* 7.24 (d, *J* = 3.5 Hz, 3H), 7.18 (s, 1H), 6.88 (s, 1H), 3.97 (s, 3H), 3.92 (s, 3H), 3.85−3.76 (m, 2H), 3.34−3.21 (m, 1H), 2.86 (m, 2H), 2.81−2.70 (m, 2H), 1.99−1.93 (m, 1H), 1.92−1.85 (m, 2H), 1.49−1.36 (m, 4H); ^13^C NMR (100 MHz, CDCl_3_) *δ* 207.6, 155.8, 152.1, 149.8, 148.8, 132.4 (q, *J* = 30 MHz), 129.5, 124.9, 121.8, 115.1, 110.6, 107.6, 104.7, 56.5, 56.3, 49.1, 49.0, 45.3, 38.8, 34.3, 33.6, 32.5, 31.5; HRESIMS *m/z* 502.1801 [M + H]^+^ (calcd for C_25_H_26_F_6_NO_3_, 502.1817).

Compound **24**, amorphous powder, 79% yield; ^1^H NMR (400 MHz, CDCl_3_) *δ* 7.60−7.58 (m, 2H), 7.42 (t, *J* = 7.6 Hz, 2H), 7.35−7.30 (m, 2H), 7.19 (s, 1H), 7.16 (t, *J* = 2.0 Hz, 1H), 7.07 (d, *J* = 7.6 Hz, 1H), 6.95 (dd, *J* = 8.2, 2.4 Hz, 1H), 6.87 (s, 1H), 3.96 (s, 3H), 3.91 (s, 3H), 3.80−3.75 (m, 2H), 3.27 (q, *J* = 7.6 Hz, 1H), 2.80−2.73 (m, 4H), 2.08−2.04 (m, 1H), 1.95−1.85 (m, 2H), 1.60−1.42 (m, 4H); ^13^C NMR (100 MHz, CDCl_3_) *δ* 207.8, 155.7, 152.4, 149.7, 148.9, 142.4, 142.1, 129.6, 129.5, 128.8, 127.4, 127.3, 118.6, 115.8, 115.7, 107.6, 104.6, 56.4, 56.3, 50.3, 50.3, 45.5, 38.9, 34.6, 33.6, 33.1, 31.9; HRESIMS *m/z* 442.2370 [M + H]^+^ (calcd for C_29_H_32_NO_3_, 442.2382).

Compound **25**, amorphous powder, 69% yield; ^1^H NMR (400 MHz, CDCl_3_) *δ* 7.55 (d, *J* = 7.2 Hz, 2H), 7.52 (d, *J* = 8.8 Hz, 2H), 7.40 (t, *J* = 8.8 Hz, 2H), 7.40 (t, *J* = 7.6 Hz, 2H), 7.29−7.27 (m, 1H), 7.19 (s, 1H), 7.02 (d, *J* = 8.8 Hz, 1H), 6.88 (s, 1H), 3.97 (s, 3H), 3.92 (s, 3H), 3.79−3.74 (m, 1H), 3.28 (q, *J* = 8.4 Hz, 1H), 2.81−2.72 (m, 4H), 2.01−1.94 (m, 1H), 1.92−1.82 (m, 2H), 1.52−1.37 (m, 4H); ^13^C NMR (100 MHz, CDCl_3_) *δ* 207.9, 155.8, 151.2, 149.7, 148.9, 141.2, 132.1, 129.5, 128.9, 127.9, 126.7, 126.5, 116.7, 107.6, 104.7, 56.4, 56.3, 50.0, 45.5, 38.9, 34.6, 33.6, 33.0, 31.9, 29.9; HRESIMS *m/z* 442.2391 [M + H]^+^ (calcd for C_29_H_32_NO_3_, 442.2382).

Compound **26**, amorphous powder, 80% yield; ^1^H NMR (400 MHz, CDCl_3_) *δ* 8.24 (d, *J* = 8.0 Hz, 1H), 8.20 (d, *J* = 8.0 Hz, 1H), 7.53−7.45 (m, 2H), 7.21 (s, 1H), 7.02 (d, *J* = 8.0 Hz, 1H), 6.89 (s, 1H), 6.74 (d, *J* = 7.6 Hz, 1H), 3.97 (s, 6H), 3.92 (s, 3H), 3.34−3.28 (m, 2H), 2.82−2.78 (m, 2H), 2.09−2.04 (m, 1H), 1.95 – 1.85 (m, 2H), 1.68−1.60 (m, 6H), 1.50−1.42 (m, 1H); ^13^C NMR (100 MHz, CDCl_3_) *δ* 208.0, 155.7, 151.6, 149.7, 149.0, 144.5, 130.3, 129.6, 129.0, 126.7, 126.2, 125.4, 123.6, 122.5, 114.6, 107.6, 104.7, 103.6, 56.4, 56.3, 55.8, 54.0, 45.8, 33.6, 32.9, 29.9; HRESIMS *m/z* 446.2319 [M + H]^+^ (calcd for C_28_H_32_NO_4_, 446.2331).

### 4.2 Biological methods

#### 4.2.1 AChE and BuChE inhibition

All reagents, AChE (from electric eel) and BuChE (from equine serum) were purchased from Sigma-Aldrich. Ellman’s method was used to measure the ChE inhibitory effects of synthesized *N*-aryl-debenzeyldonepezils (Ellman et al., 1961). The tested compounds were dissolved in DMSO and diluted with phosphate buffer to final concentrations. The enzyme solution was prepared by dissolving 2.5 mg (0.5 U/mL) AChE in 1 mL pH 8.0 phosphate buffer. In 96-well plates, 140 μL phosphate buffer (pH 6.7) and 10 μL AChE were incubated with 10 μL of various concentrations of test compounds at 25°C for 20 min. Then, 10 *μ*L DTNB (0.75 mM) and 10 μL ATCI (1.5 mM) were added for incubation at 37°C for 20 min. The absorbance was monitored at 405 nm using a microplate reader (TECAN SPECTRA, Wetzlar, Germany). Each concentration was assayed in triplicate. BuChE assays were present using a similar method to that described above. Enzyme inhibitory activity (%) = [1 – (A_sample_/A_control_)] × 100.

#### 4.2.2 Cell culture and treatment

The neuroprotective activity was evaluated in human neuroblastoma SH-SY5Y cells which were treated with H_2_O_2_. Human SH-SY5Y neuroblastoma cells were obtained from ATCC (Manassas, VA, United States) and were cultured at 37°C in a humidified atmosphere of 5% CO_2_, in Gibcotm Dulbecco’s Modified Eagle Medium (DMEM) supplemented with 10% heat-inactivated fetal bovine serum. The viability of the cells was determined by MTT assay. MTT was purchased from Sigma-Aldrich (St. Louis, MO, United States). SH-SY5Y cells were seeded in 96-well plates at a density of 1 × 10^5^ cells/well for 12 h. Cells were pretreated with 50 μM of each test compound. After 24 h of incubation, hydrogen peroxide solution (H_2_O_2_, 700 μM) was added and incubated for 4 h. Then, MTT (5 mg/mL) was added to each well. After a 4 h treatment, the supernatant was removed, and DMSO (150 μL/well) was added to dissolve the insoluble formazan crystals. Then, the plate was vibrated, and the absorbance was measured at 490 nm using a microplate reader.

#### 4.2.3 Molecular docking analysis

The ligand-protein docking was guided by Discovery Studio 2020 Client and PyRx software to predict the binding poses of the ligand in the active site of AChE. The 3D models of AChE (PDB ID:1acj) and BuChE (PDB ID: 6ASM) were obtained from Protein Data Bank (http://www.rcsb.org/). The 3D models of the protein of AchE and BuChE were further optimized by Discovery Studio 2020 Client to minimize energy. The AutoDock Vina was employed for docking calculations using the default parameters. The blood-brain barrier (BBB) penetration was predicted by the Discovery Studio 2020 Client.

## Data Availability

The raw data supporting the conclusion of this article will be made available by the authors, without undue reservation.
