# Supplementary material for: Palladium-catalyzed synthesis and anti-AD biological activity evaluation of N-aryl-debenzeyldonepezil analogues
Source: Front Chem. 2023 Dec 8;11:1282978. doi: 10.3389/fchem.2023.1282978 (PMC10748399; doi:10.3389/fchem.2023.1282978)
Supplement: Supplementary file 1 [file DataSheet1.PDF]

Supplementary Material

Palladium-Catalyzed Synthesis and Anti-AD Biological Activity Evaluation of *N*-aryl-debenzeyldonepezil Analogues

Jing-Jing Xu[1*], Jiao Luo[1], Heng Xi[1], Jin-Bu Xu[3], Lin-Xi Wan[2*]

[1] Department of Pharmacy, The Third People's Hospital of Chengdu, Chengdu 610041, People's Republic of China;

[2] Sichuan Research Center for Drug Precision Industrial Technology, West China School of Pharmacy, Sichuan University, Chengdu 610041, People's Republic of China

[3] Sichuan Engineering Research Center for Biomimetic Synthesis of Natural Drugs, School of Life Science and Engineering, Southwest Jiaotong University, Chengdu 610031, People's Republic of China

*** Correspondence:** xujingjing0224@163.com (J.-J. Xu); wanlx@scu.edu.cn (L.-X. Wan)

Content

Figure 1. ^{1}H NMR spectrum of debenzyldonepezil ($CDCl_3$) ... 4
Figure 2. ^{13}C NMR spectrum of debenzyldonepezil ($CDCl_3$) ... 4
Figure 3. ^{1}H NMR spectrum of compound **1** ($CDCl_3$) ... 5
Figure 4. ^{13}C NMR spectrum of compound **1** ($CDCl_3$) ... 5
Figure 5. ^{1}H NMR spectrum of compound **2** ($CDCl_3$) ... 6
Figure 6. ^{13}C NMR spectrum of compound **2** ($CDCl_3$) ... 6
Figure 7. ^{1}H NMR spectrum of compound **3** ($CDCl_3$) ... 7
Figure 8. ^{13}C NMR spectrum of compound **3** ($CDCl_3$) ... 7
Figure 9. ^{1}H NMR spectrum of compound **4** ($CDCl_3$) ... 8
Figure 10. ^{13}C NMR spectrum of compound **4** ($CDCl_3$) ... 8
Figure 11. ^{1}H NMR spectrum of compound **5** ($CDCl_3$) ... 9
Figure 12. ^{13}C NMR spectrum of compound **5** ($CDCl_3$) ... 9
Figure 13. ^{1}H NMR spectrum of compound **6** ($CDCl_3$) ... 10
Figure 14. ^{13}C NMR spectrum of compound **6** ($CDCl_3$) ... 10
Figure 15. ^{1}H NMR spectrum of compound **7** ($CDCl_3$) ... 11
Figure 16. ^{13}C NMR spectrum of compound **7** ($CDCl_3$) ... 11
Figure 17. ^{1}H NMR spectrum of compound **8** ($CDCl_3$) ... 12
Figure 18. ^{13}C NMR spectrum of compound **8** ($CDCl_3$) ... 12
Figure 19. ^{1}H NMR spectrum of compound **9** ($CDCl_3$) ... 13
Figure 20. ^{13}C NMR spectrum of compound **9** ($CDCl_3$) ... 13
Figure 21. ^{1}H NMR spectrum of compound **10** ($CDCl_3$) ... 14
Figure 22. ^{13}C NMR spectrum of compound **10** ($CDCl_3$) ... 14
Figure 23. ^{1}H NMR spectrum of compound **11** ($CDCl_3$) ... 15
Figure 24. ^{13}C NMR spectrum of compound **11** ($CDCl_3$) ... 15
Figure 25. ^{1}H NMR spectrum of compound **12** ($CDCl_3$) ... 16
Figure 26. ^{13}C NMR spectrum of compound **12** ($CDCl_3$) ... 16
Figure 27. ^{1}H NMR spectrum of compound **13** ($CDCl_3$) ... 17
Figure 28. ^{13}C NMR spectrum of compound **13** ($CDCl_3$) ... 17
Figure 29. ^{1}H NMR spectrum of compound **14** ($CDCl_3$) ... 18
Figure 30. ^{13}C NMR spectrum of compound **14** ($CDCl_3$) ... 18
Figure 31. ^{1}H NMR spectrum of compound **15** ($CDCl_3$) ... 19
Figure 32. ^{13}C NMR spectrum of compound **15** ($CDCl_3$) ... 19
Figure 33. ^{1}H NMR spectrum of compound **16** ($CDCl_3$) ... 20
Figure 34. ^{13}C NMR spectrum of compound **16** ($CDCl_3$) ... 20
Figure 35. ^{1}H NMR spectrum of compound **17** ($CDCl_3$) ... 21
Figure 36. ^{13}C NMR spectrum of compound **17** ($CDCl_3$) ... 21
Figure 37. ^{1}H NMR spectrum of compound **18** ($CDCl_3$) ... 22
Figure 38. ^{13}C NMR spectrum of compound **18** ($CDCl_3$) ... 22
Figure 39. ^{1}H NMR spectrum of compound **19** ($CDCl_3$) ... 23
Figure 40. ^{13}C NMR spectrum of compound **19** ($CDCl_3$) ... 23
Figure 41. ^{1}H NMR spectrum of compound **20** ($CDCl_3$) ... 24
Figure 42. ^{13}C NMR spectrum of compound **20** ($CDCl_3$) ... 24
Figure 43. ^{1}H NMR spectrum of compound **21** ($CDCl_3$) ... 25
Figure 44. ^{13}C NMR spectrum of compound **21** ($CDCl_3$) ... 25
Figure 45. ^{1}H NMR spectrum of compound **22** ($CDCl_3$) ... 26
Figure 46. ^{13}C NMR spectrum of compound **22** ($CDCl_3$) ... 26

Figure 47. ^{1}H NMR spectrum of compound **23** ($CDCl_3$) 27
Figure 48. ^{13}C NMR spectrum of compound **23** ($CDCl_3$) 27
Figure 49. ^{1}H NMR spectrum of compound **24** ($CDCl_3$) 28
Figure 50. ^{13}C NMR spectrum of compound **24** ($CDCl_3$) 28
Figure 51. ^{1}H NMR spectrum of compound **25** ($CDCl_3$) 29
Figure 52. ^{13}C NMR spectrum of compound **25** ($CDCl_3$) 29
Figure 53. ^{1}H NMR spectrum of compound **26** ($CDCl_3$) 30
Figure 54. ^{13}C NMR spectrum of compound **26** ($CDCl_3$) 30

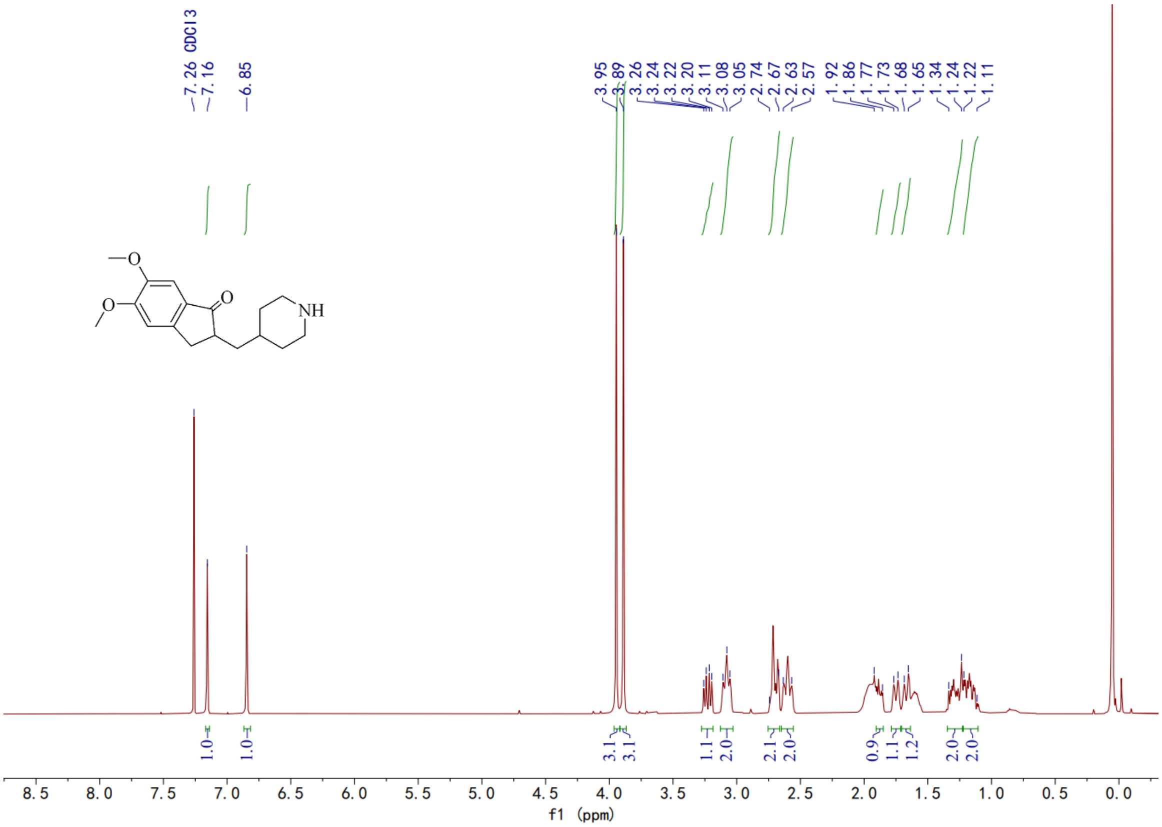

Figure 1. ^{1}H NMR spectrum of debenzyldonepezil ($CDCl_3$)

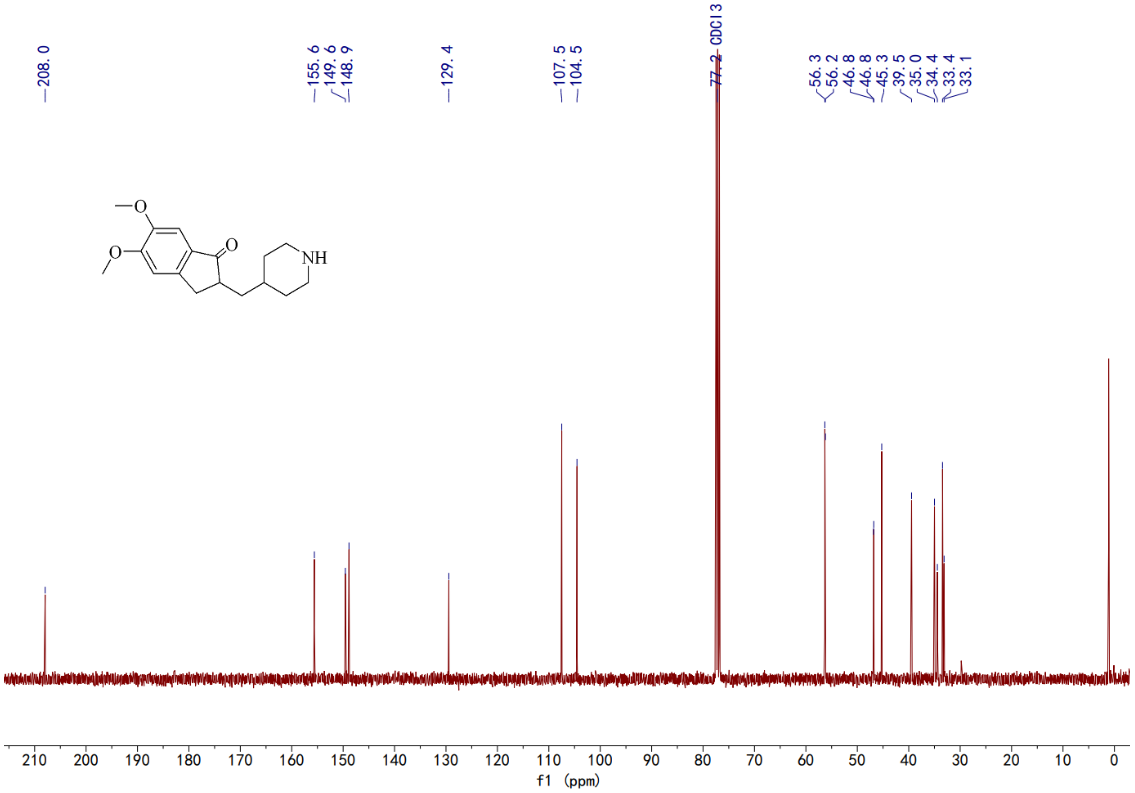

Figure 2. ^{13}C NMR spectrum of debenzyldonepezil ($CDCl_3$)

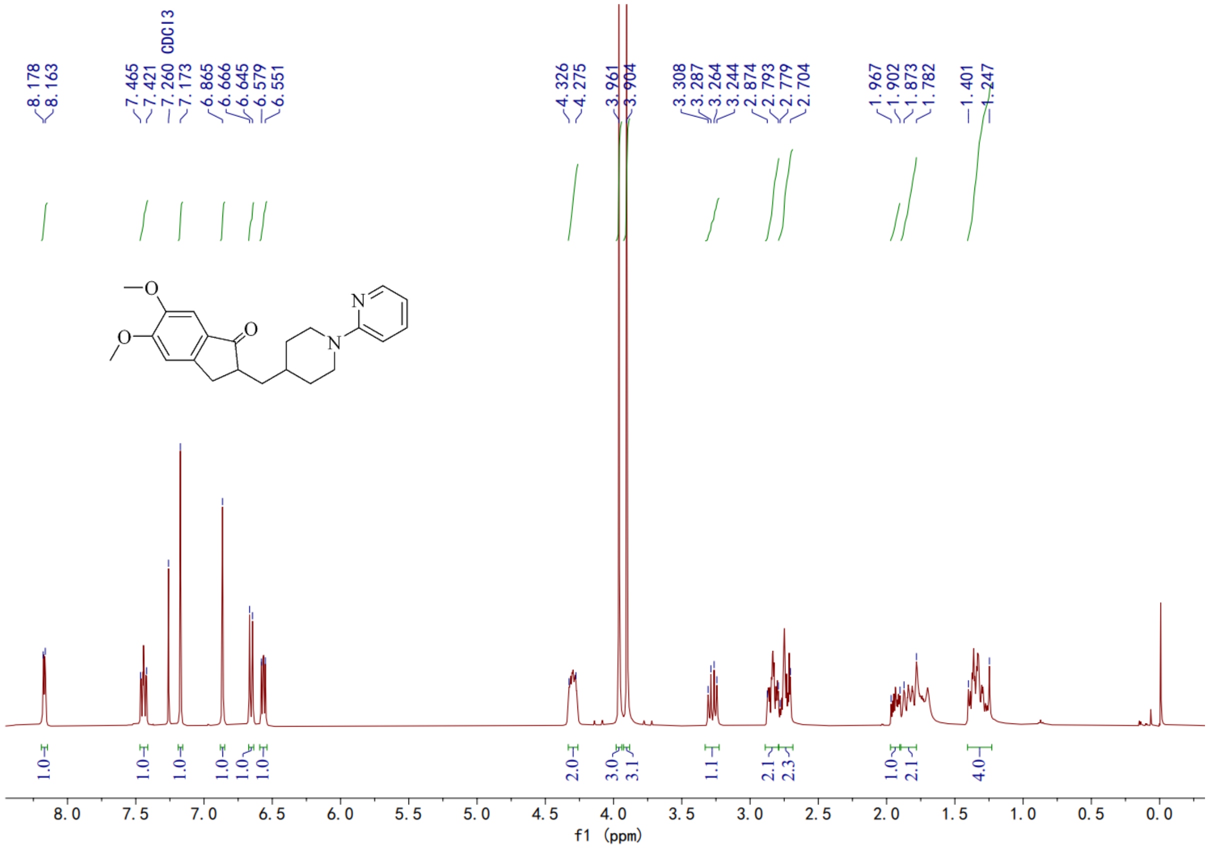

Figure 3. ^{1}H NMR spectrum of compound **1** ($CDCl_3$)

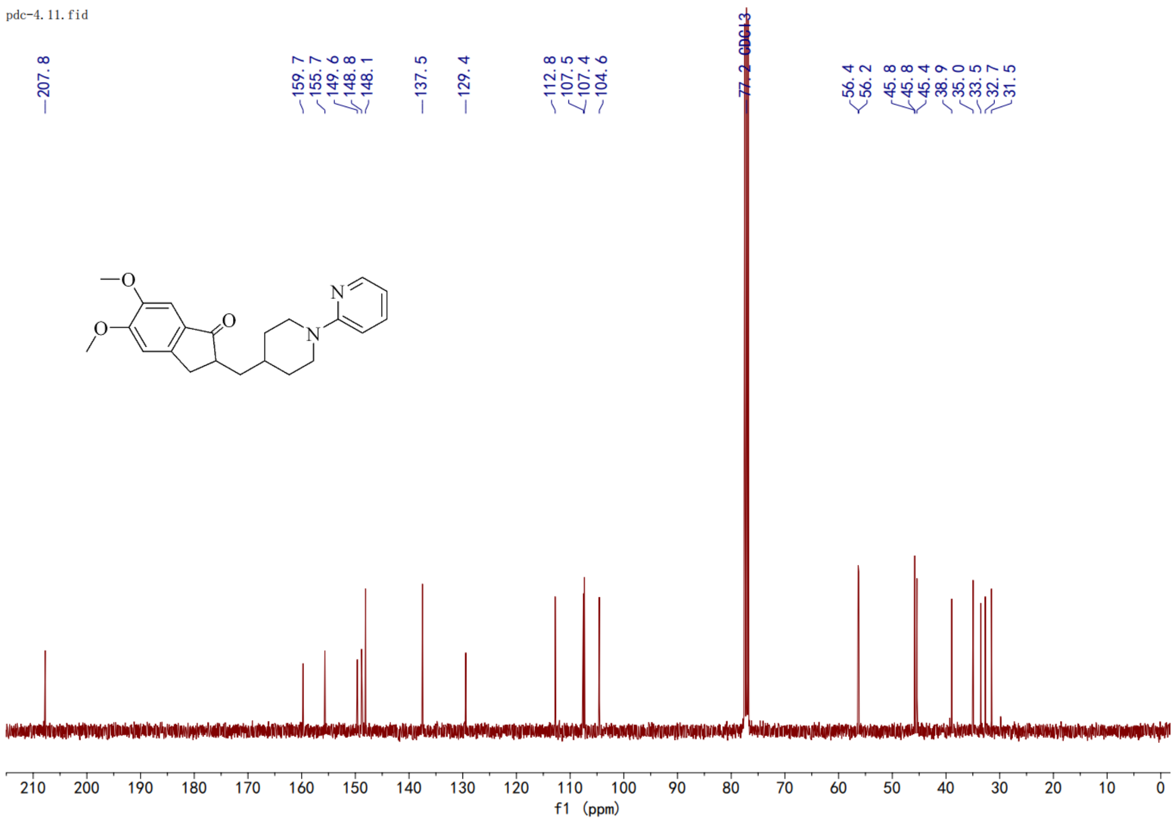

Figure 4. ^{13}C NMR spectrum of compound **1** ($CDCl_3$)

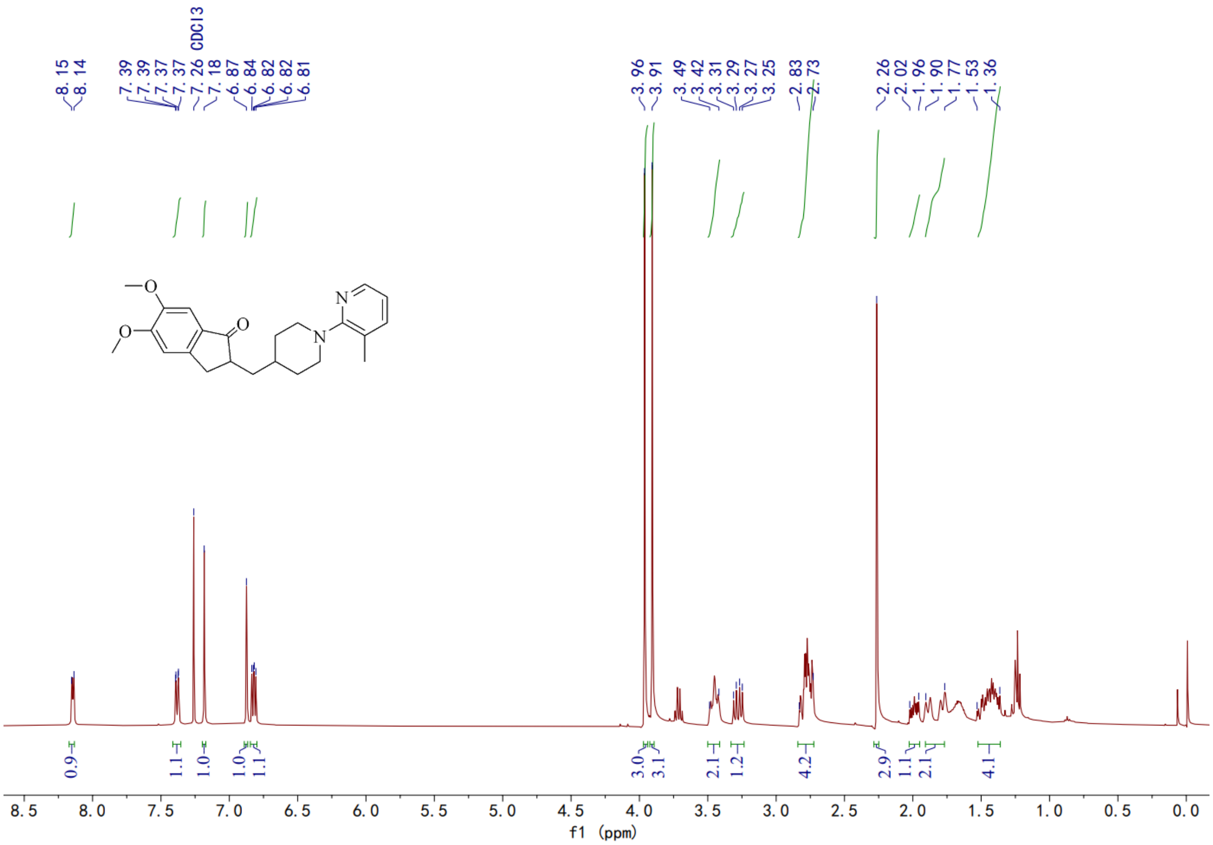

Figure 5. ^{1}H NMR spectrum of compound **2** ($CDCl_3$)

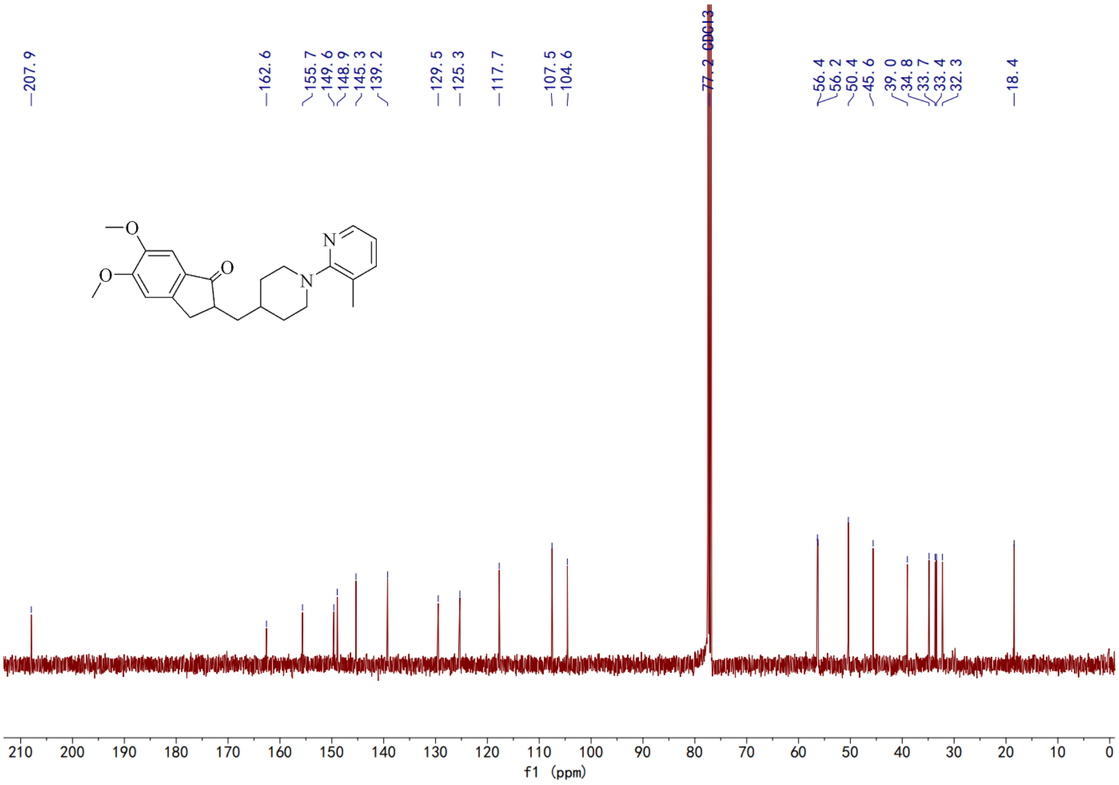

Figure 6. ^{13}C NMR spectrum of compound **2** ($CDCl_3$)

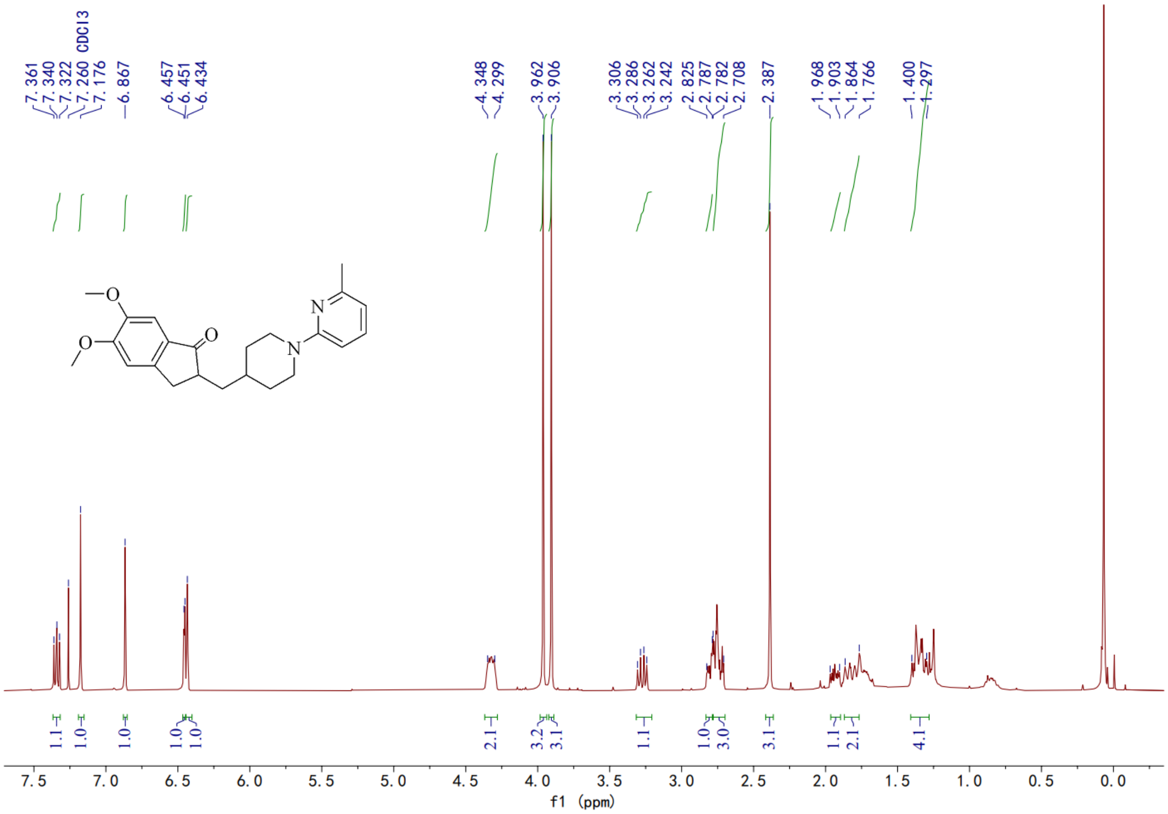

Figure 7. ^{1}H NMR spectrum of compound **3** ($CDCl_3$)

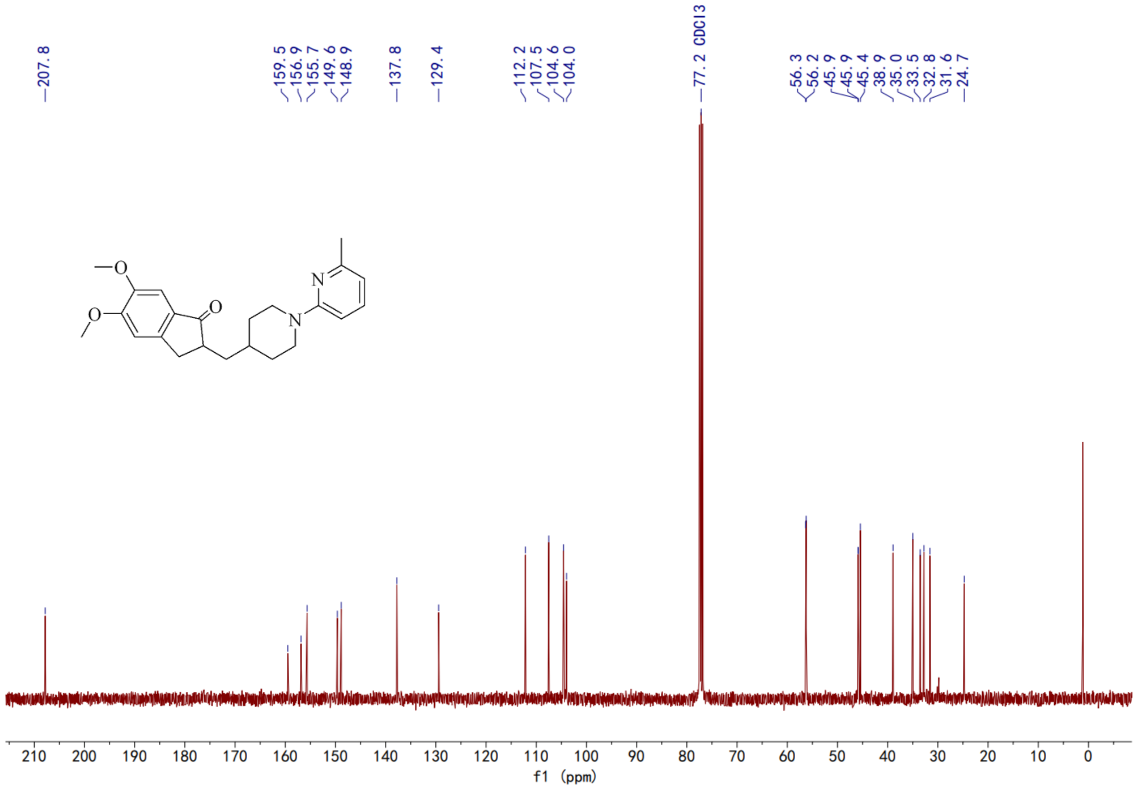

Figure 8. ^{13}C NMR spectrum of compound **3** ($CDCl_3$)

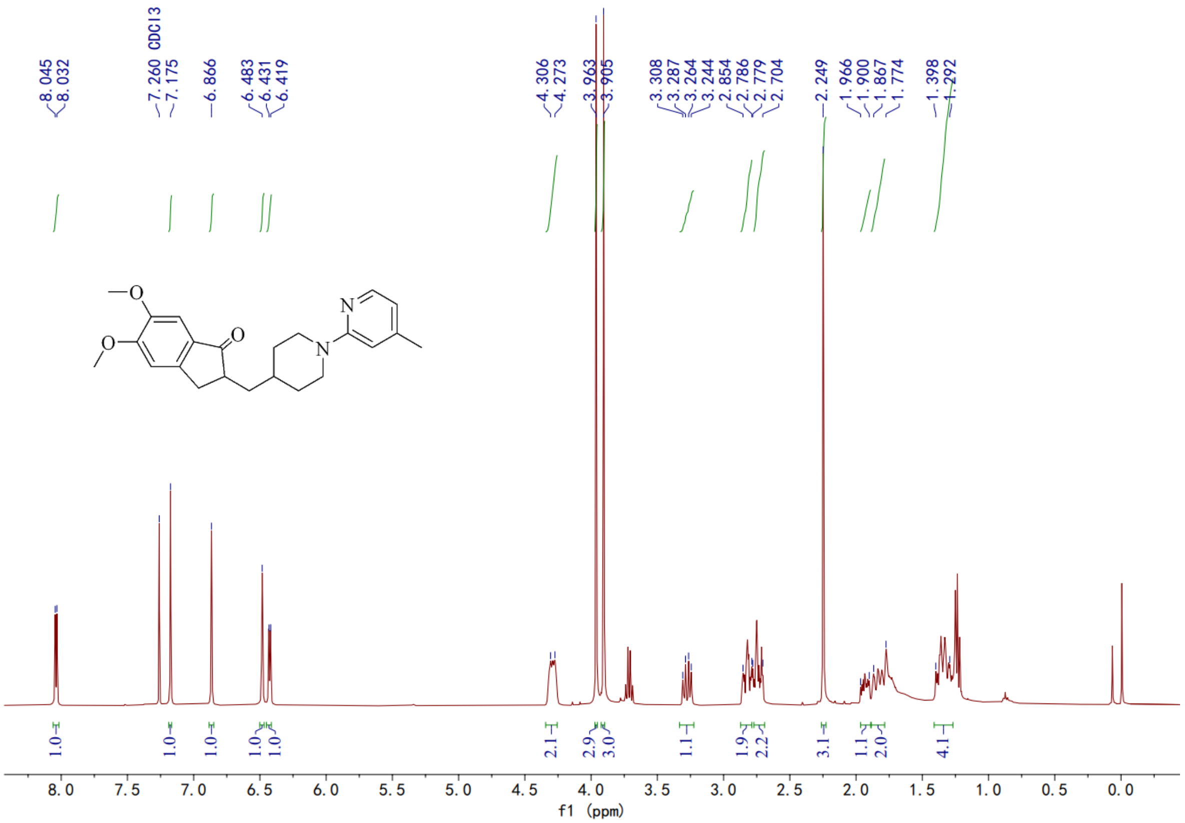

Figure 9. ^{1}H NMR spectrum of compound **4** ($CDCl_3$)

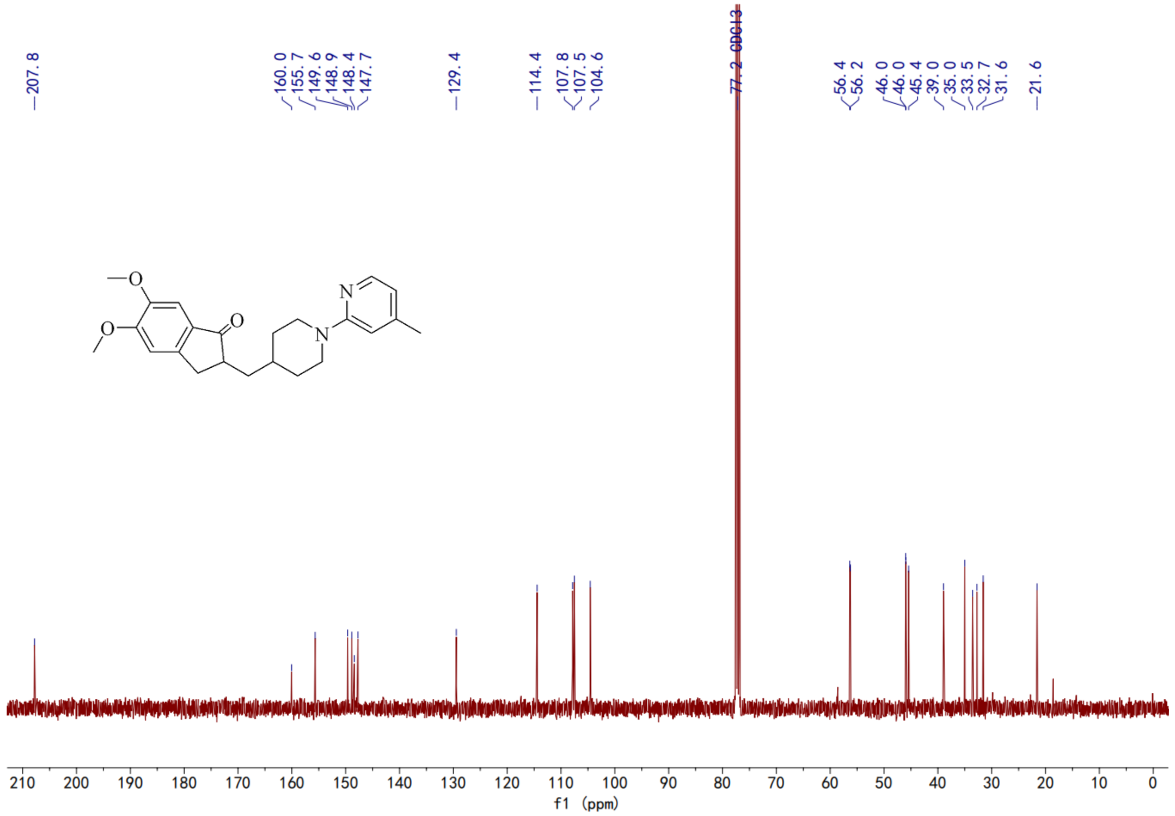

Figure 10. ^{13}C NMR spectrum of compound **4** ($CDCl_3$)

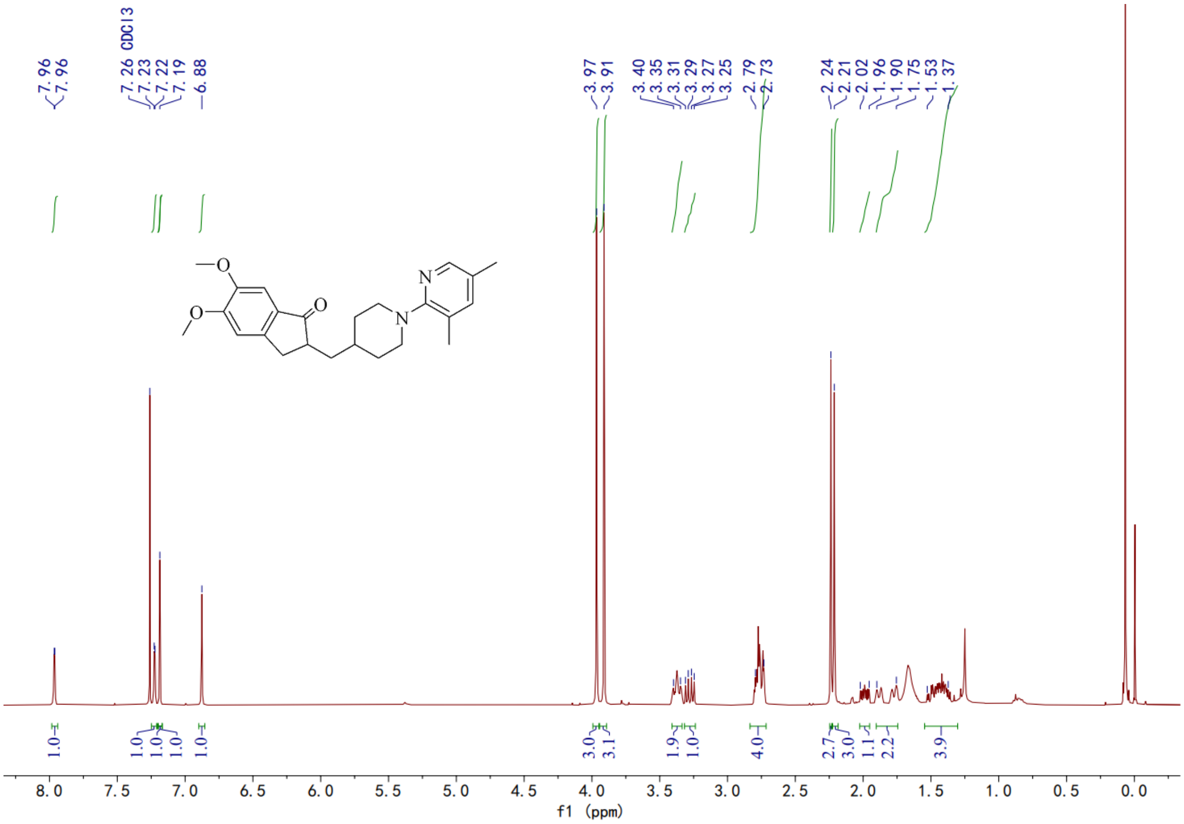

Figure 11. ^{1}H NMR spectrum of compound **5** ($CDCl_3$)

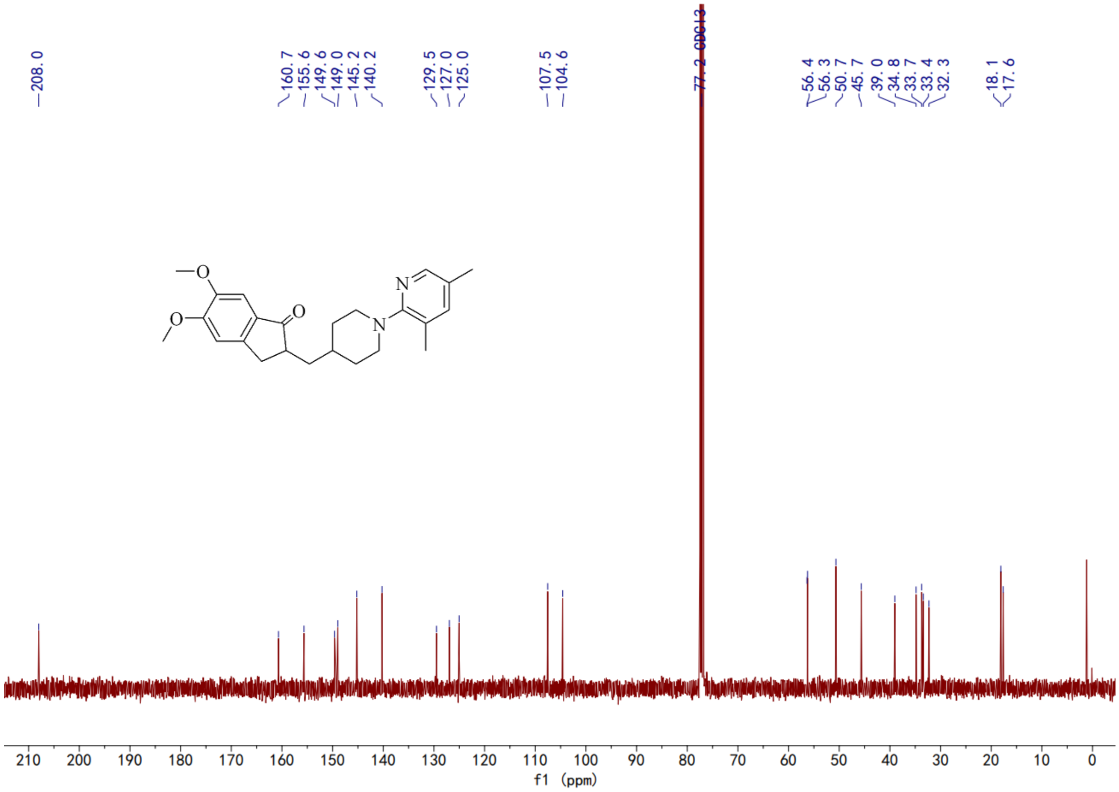

Figure 12. ^{13}C NMR spectrum of compound **5** ($CDCl_3$)

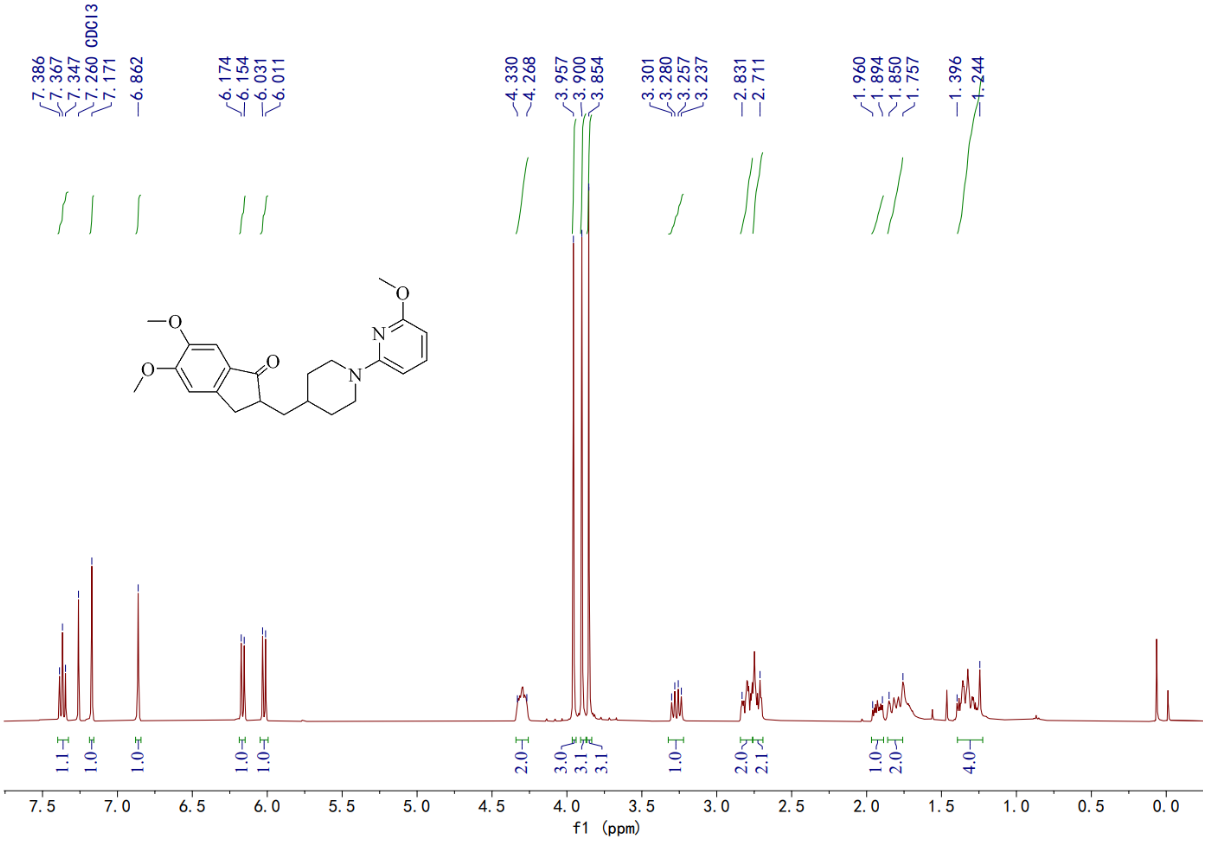

Figure 13. ^{1}H NMR spectrum of compound **6** ($CDCl_3$)

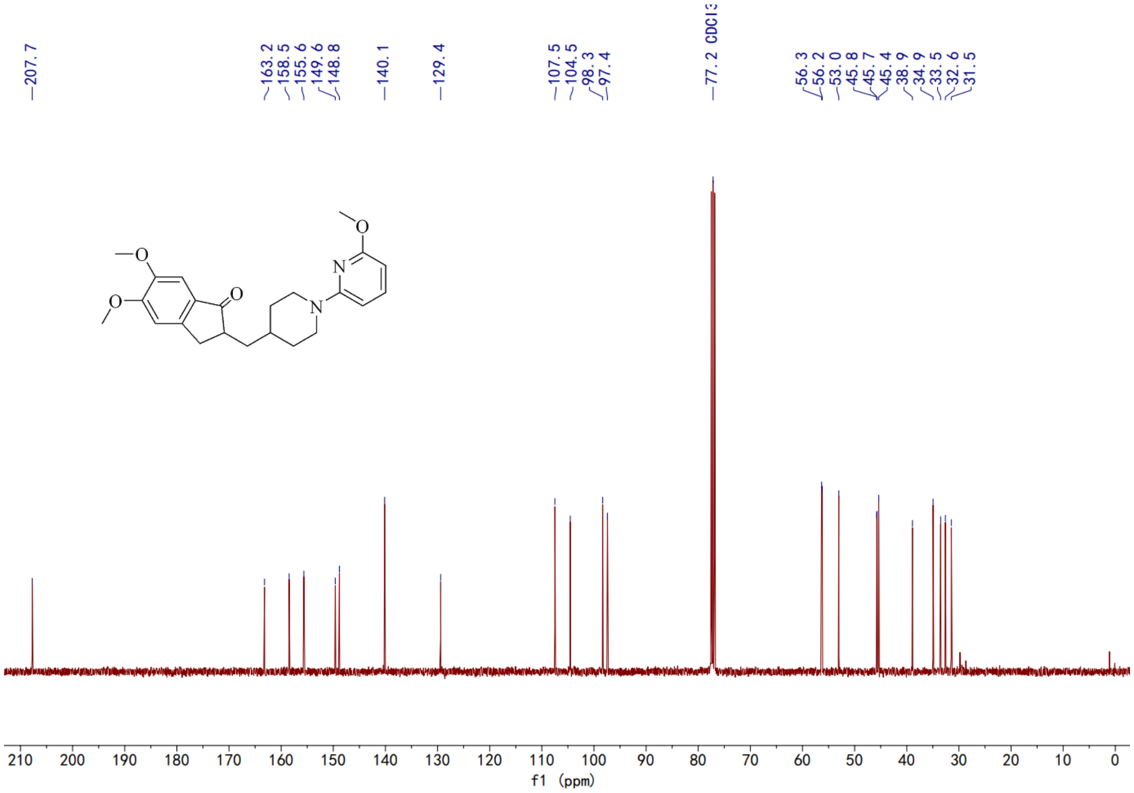

Figure 14. ^{13}C NMR spectrum of compound **6** ($CDCl_3$)

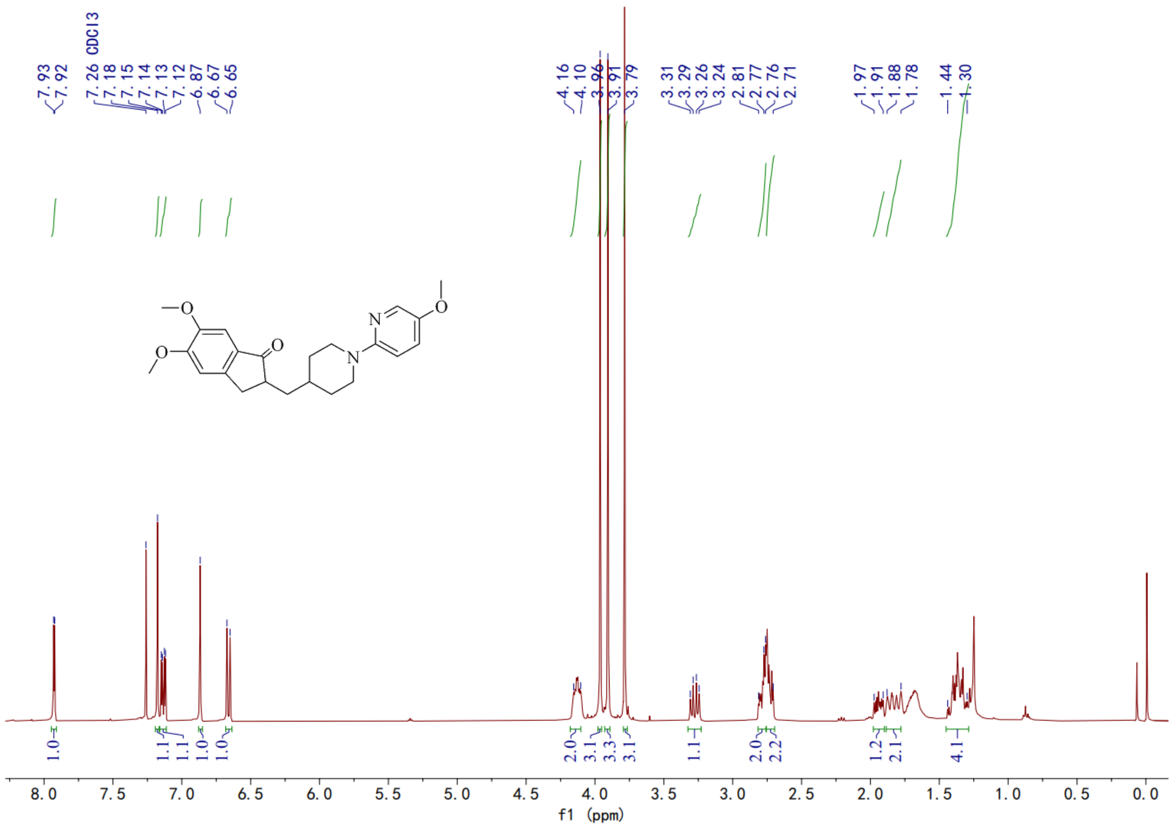

Figure 15. ^{1}H NMR spectrum of compound **7** ($CDCl_3$)

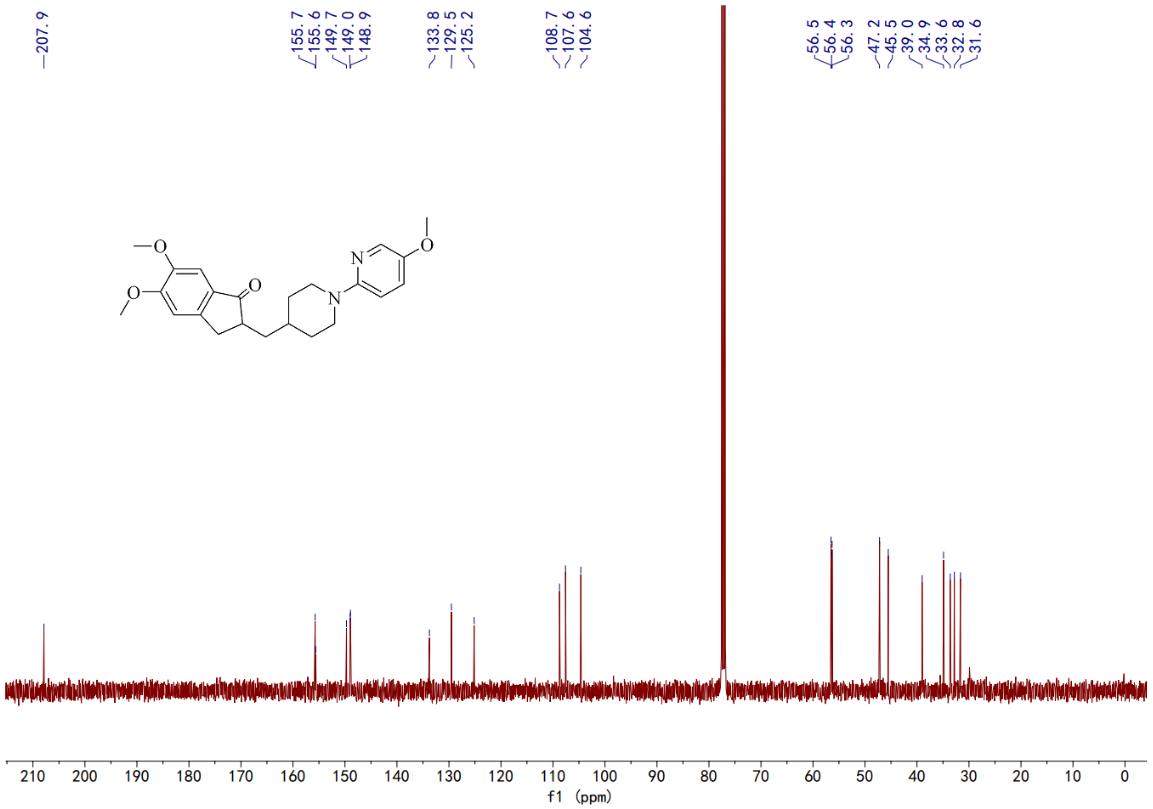

Figure 16. ^{13}C NMR spectrum of compound **7** ($CDCl_3$)

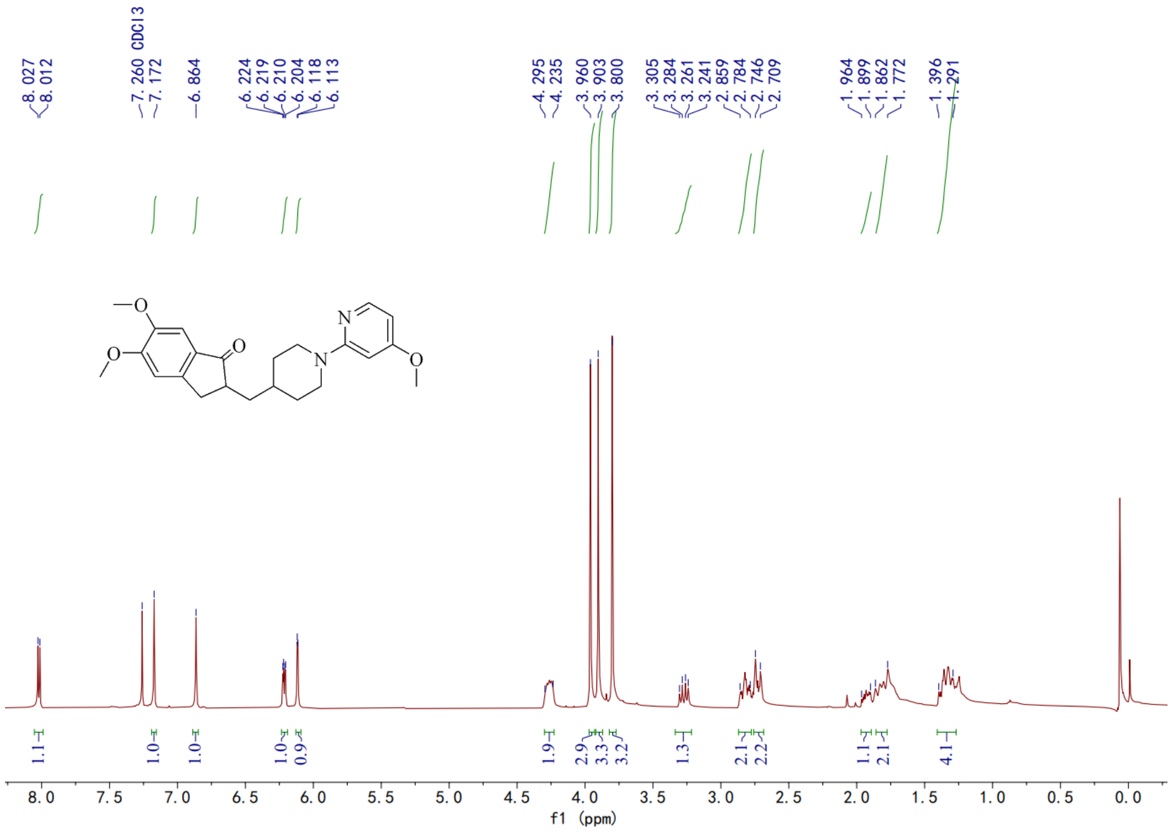

Figure 17. ^{1}H NMR spectrum of compound **8** ($CDCl_3$)

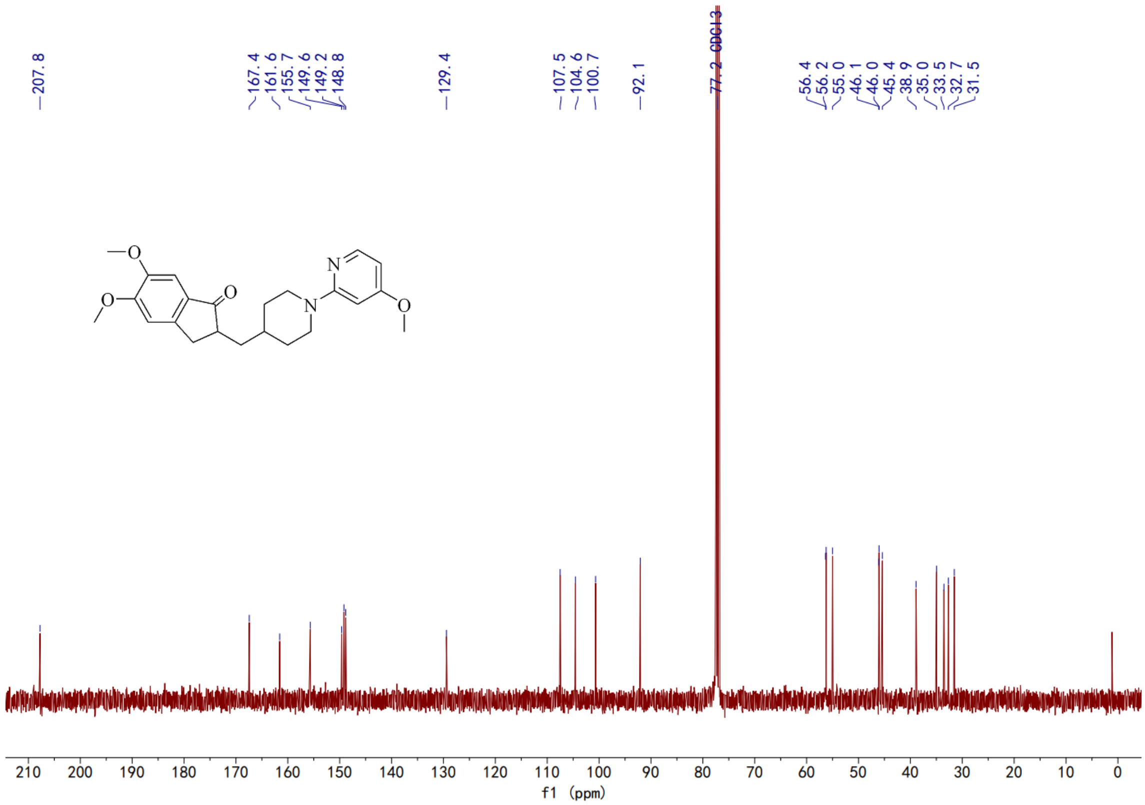

Figure 18. ^{13}C NMR spectrum of compound **8** ($CDCl_3$)

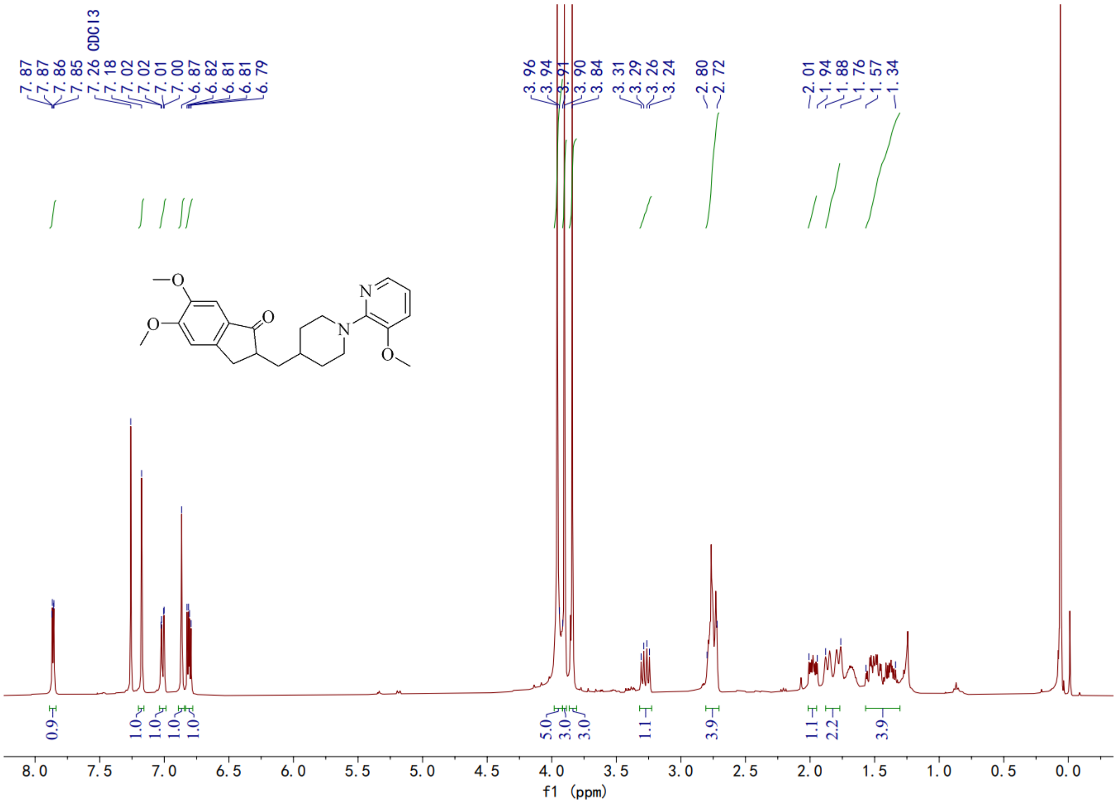

Figure 19. ^{1}H NMR spectrum of compound **9** ($CDCl_3$)

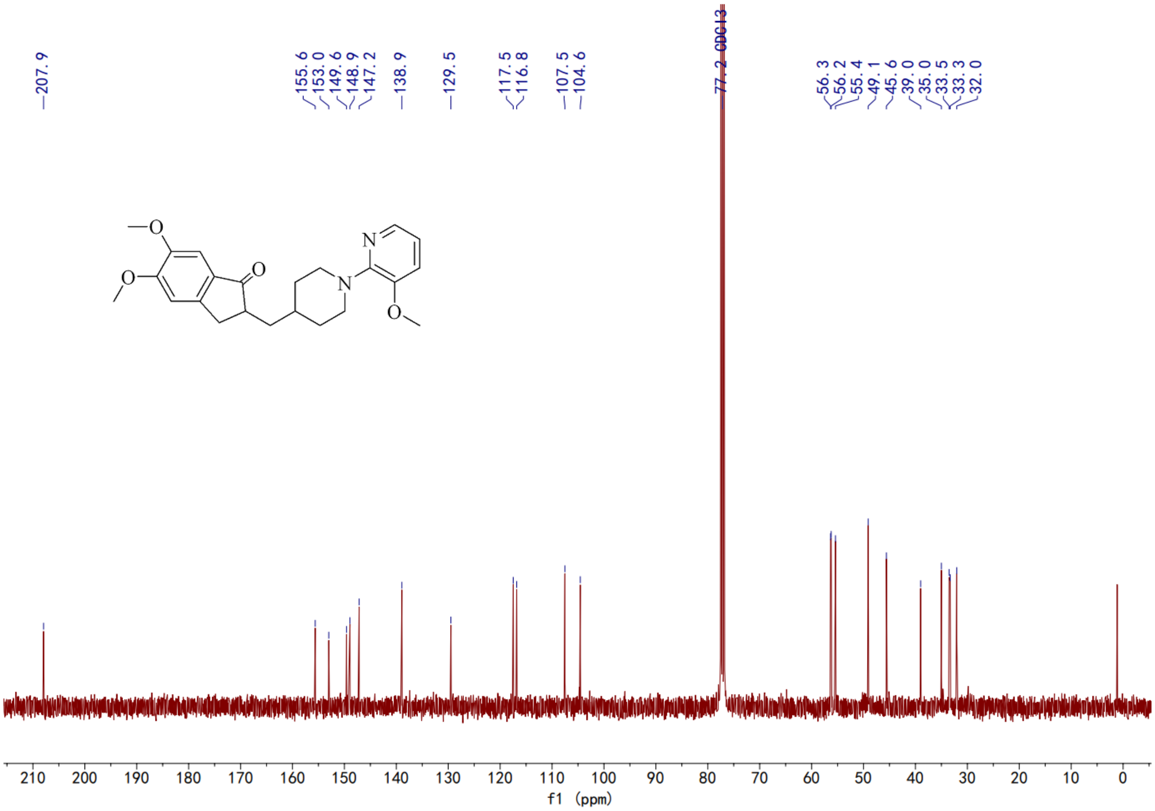

Figure 20. ^{13}C NMR spectrum of compound **9** ($CDCl_3$)

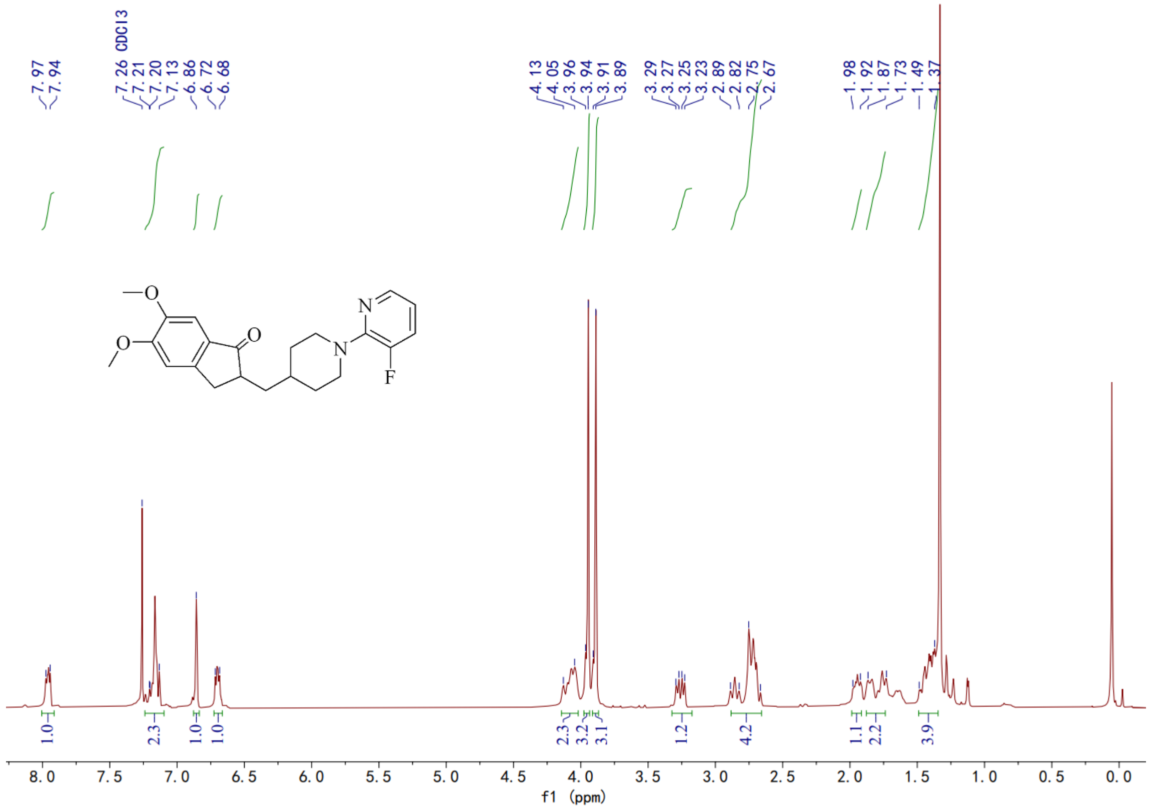

Figure 21. ^{1}H NMR spectrum of compound **10** ($CDCl_3$)

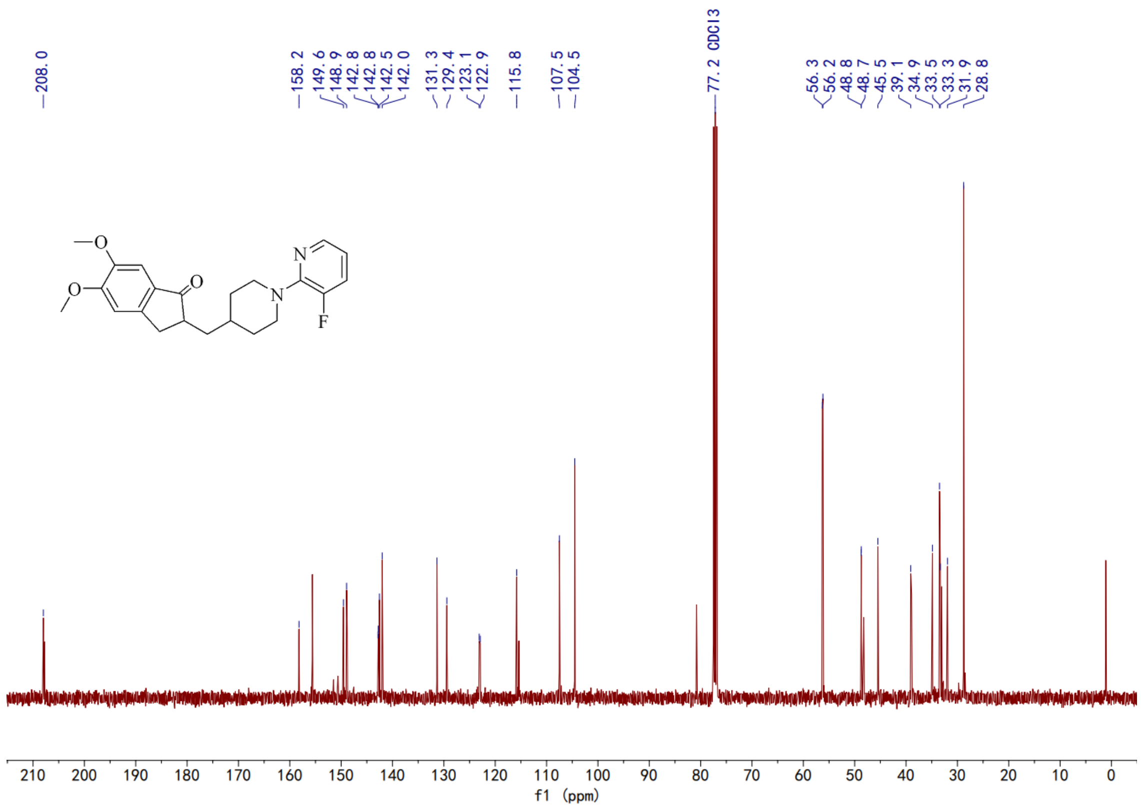

Figure 22. ^{13}C NMR spectrum of compound **10** ($CDCl_3$)

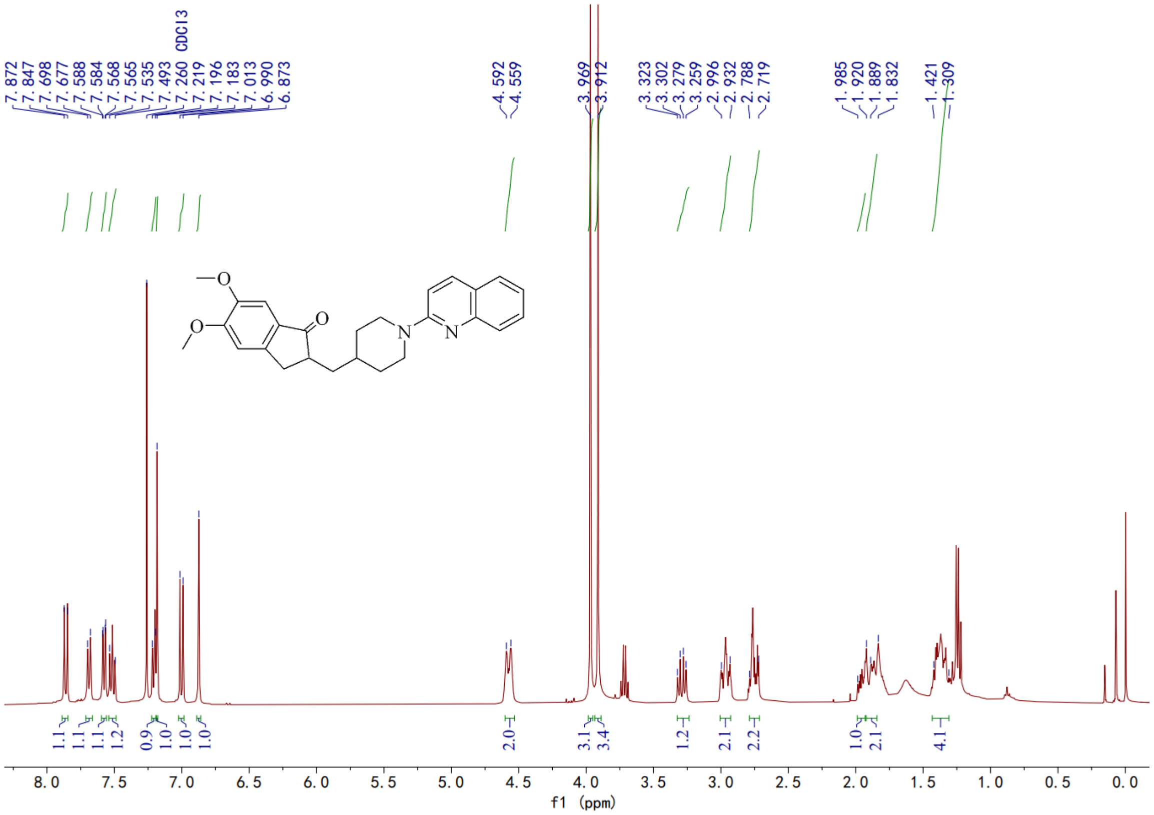

Figure 23. ^{1}H NMR spectrum of compound **11** ($CDCl_3$)

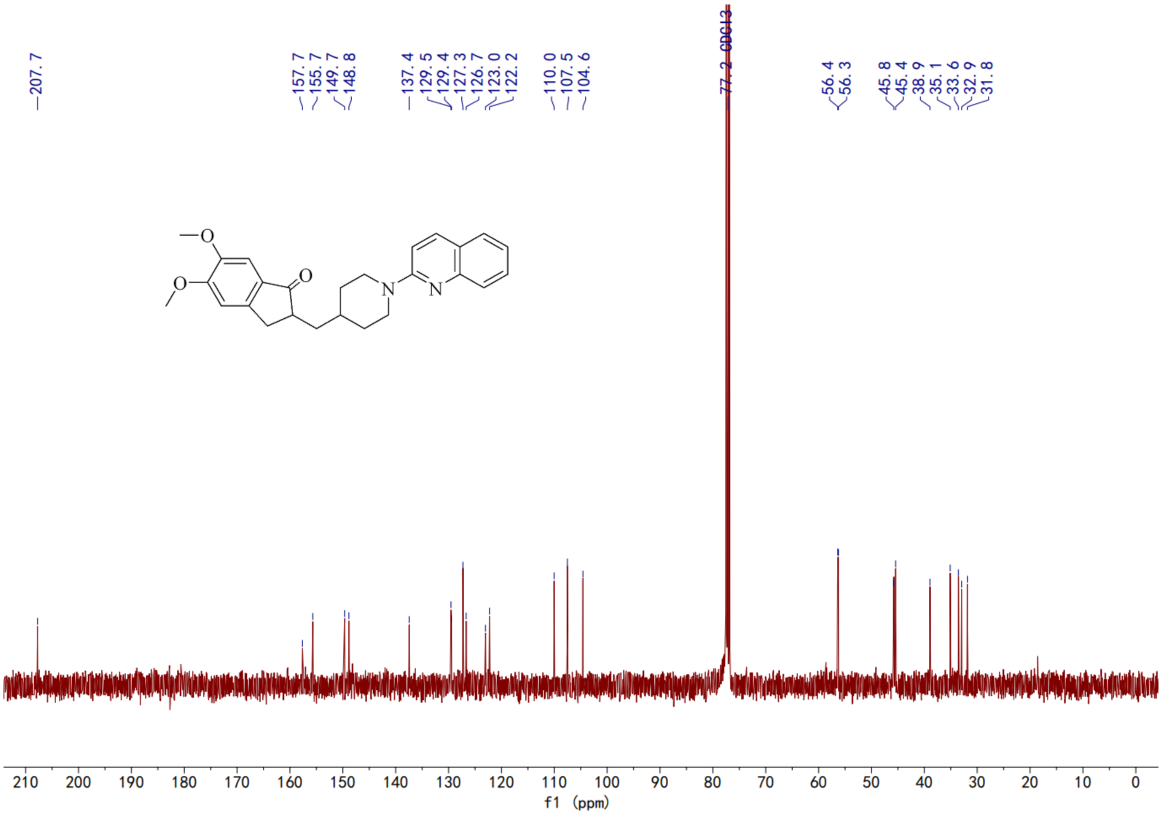

Figure 24. ^{13}C NMR spectrum of compound **11** ($CDCl_3$)

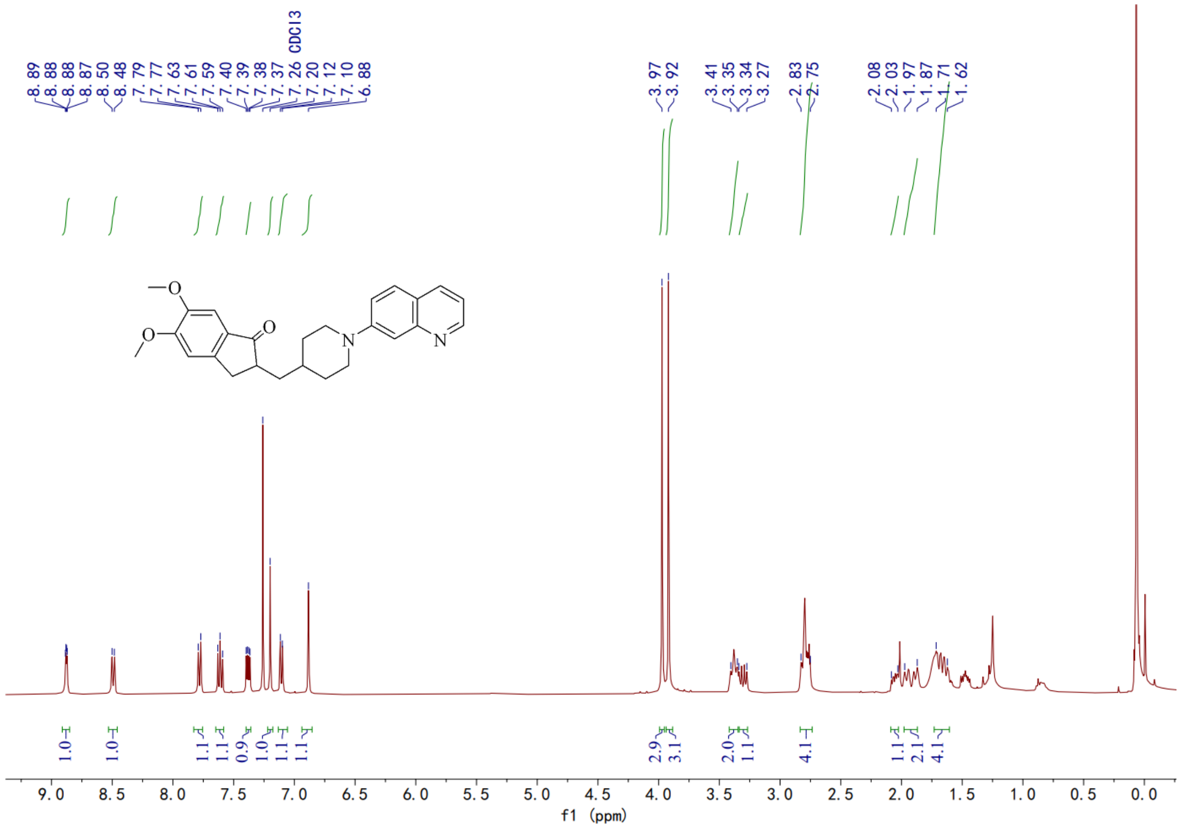

Figure 25. ^{1}H NMR spectrum of compound **12** ($CDCl_3$)

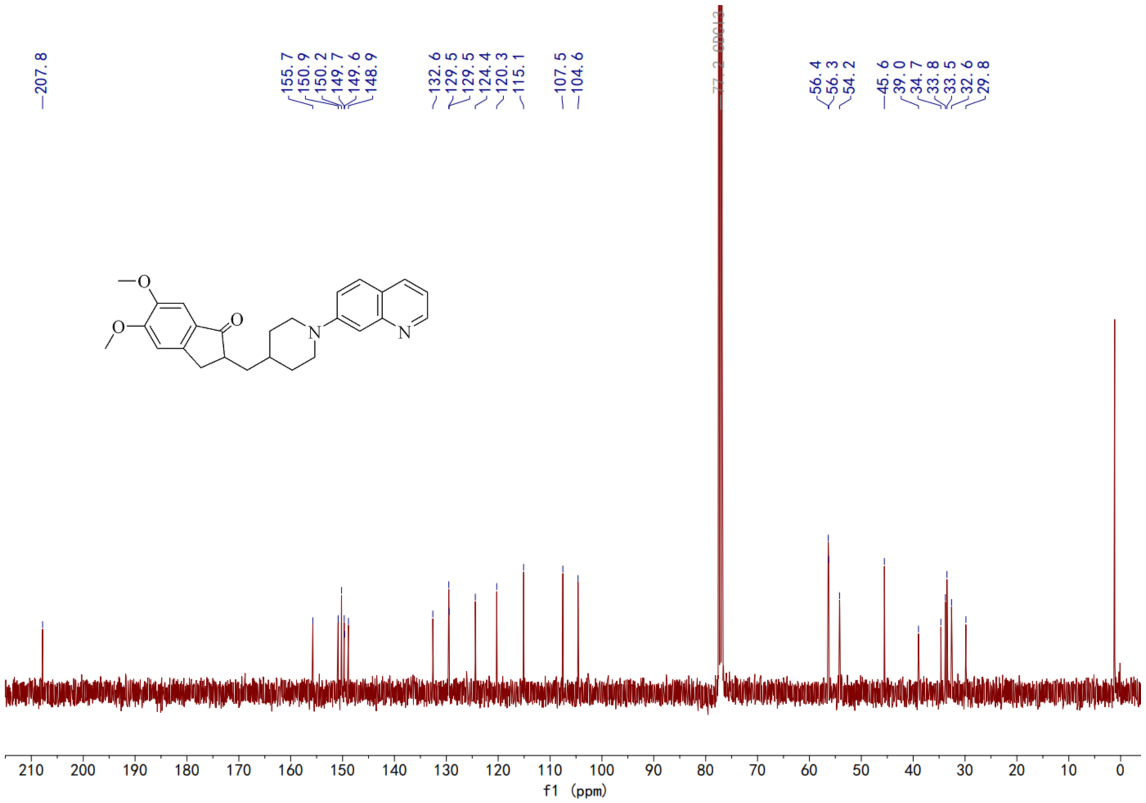

Figure 26. ^{13}C NMR spectrum of compound **12** ($CDCl_3$)

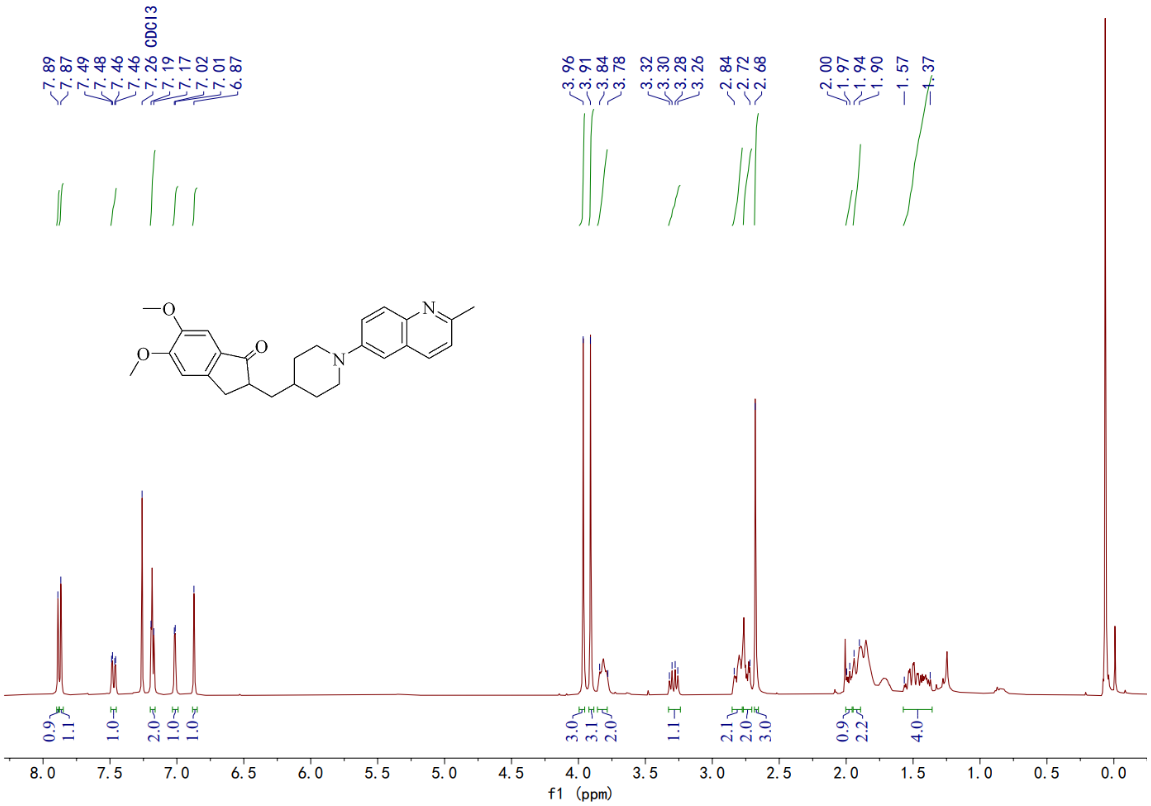

Figure 27. ^{1}H NMR spectrum of compound **13** ($CDCl_3$)

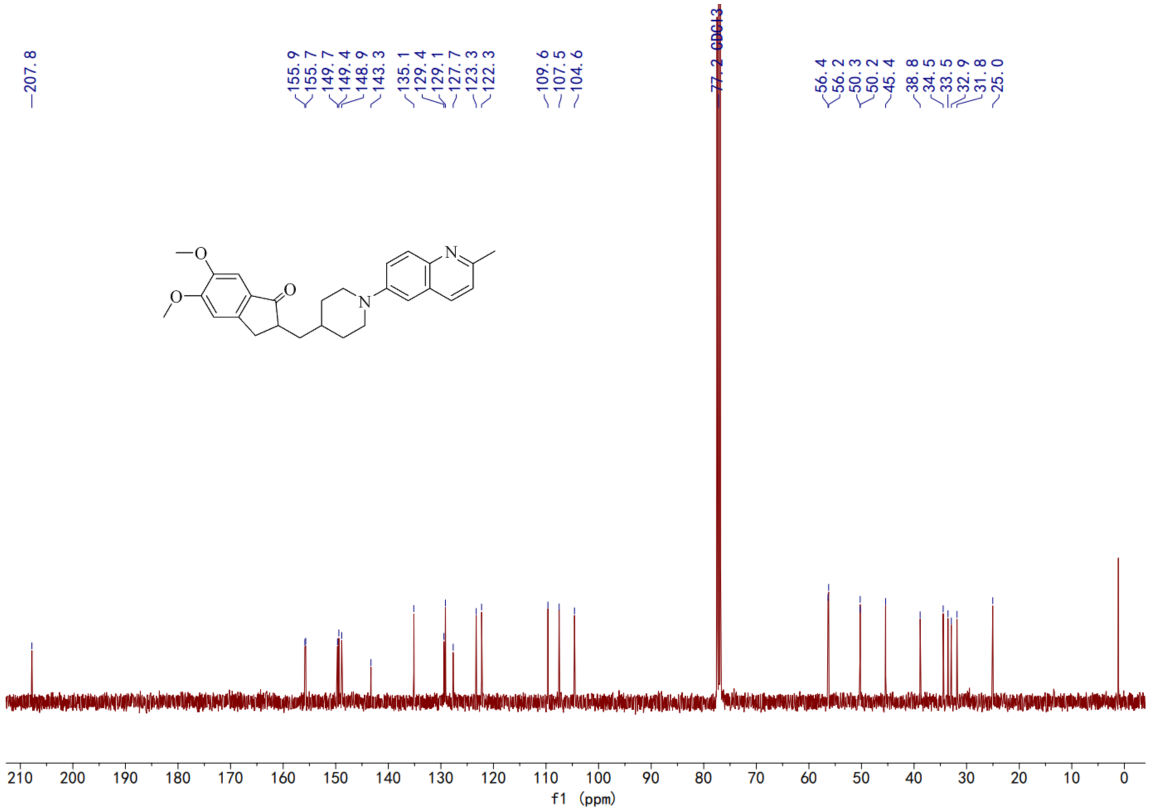

Figure 28. ^{13}C NMR spectrum of compound **13** ($CDCl_3$)

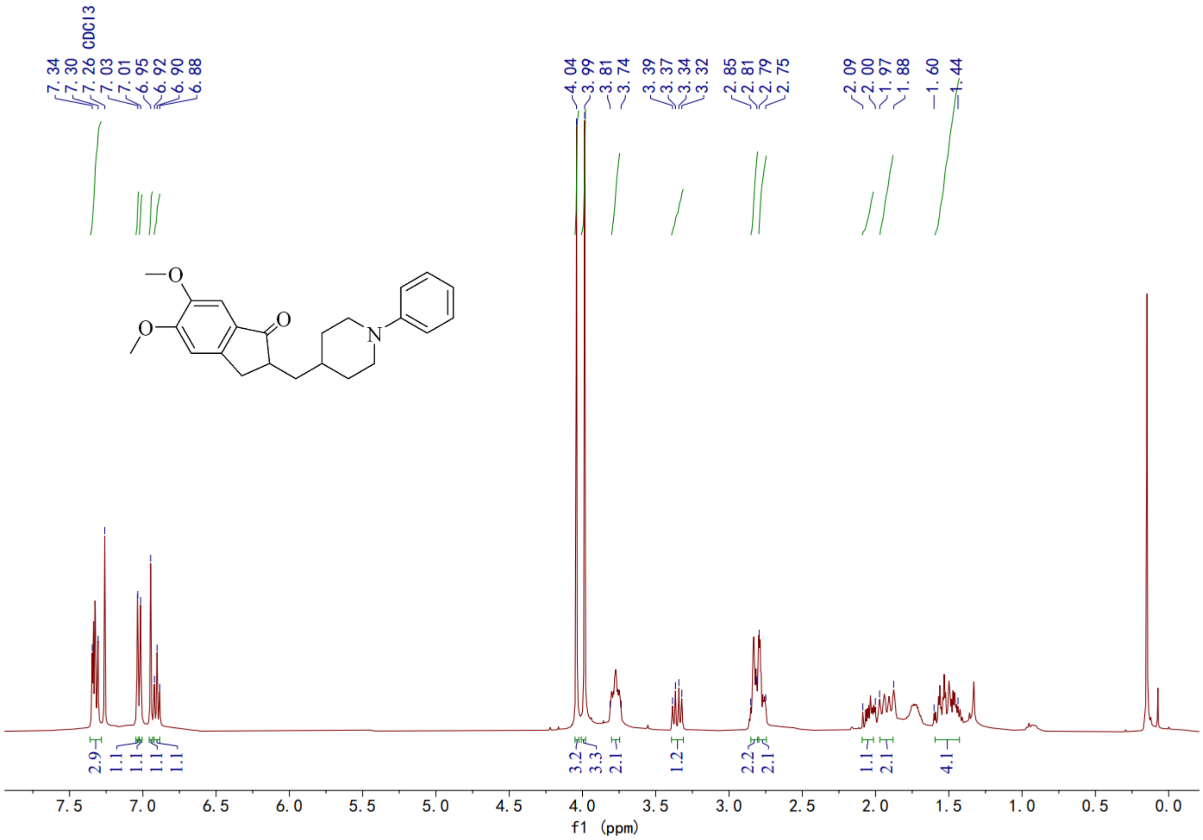

Figure 29. ^{1}H NMR spectrum of compound **14** ($CDCl_3$)

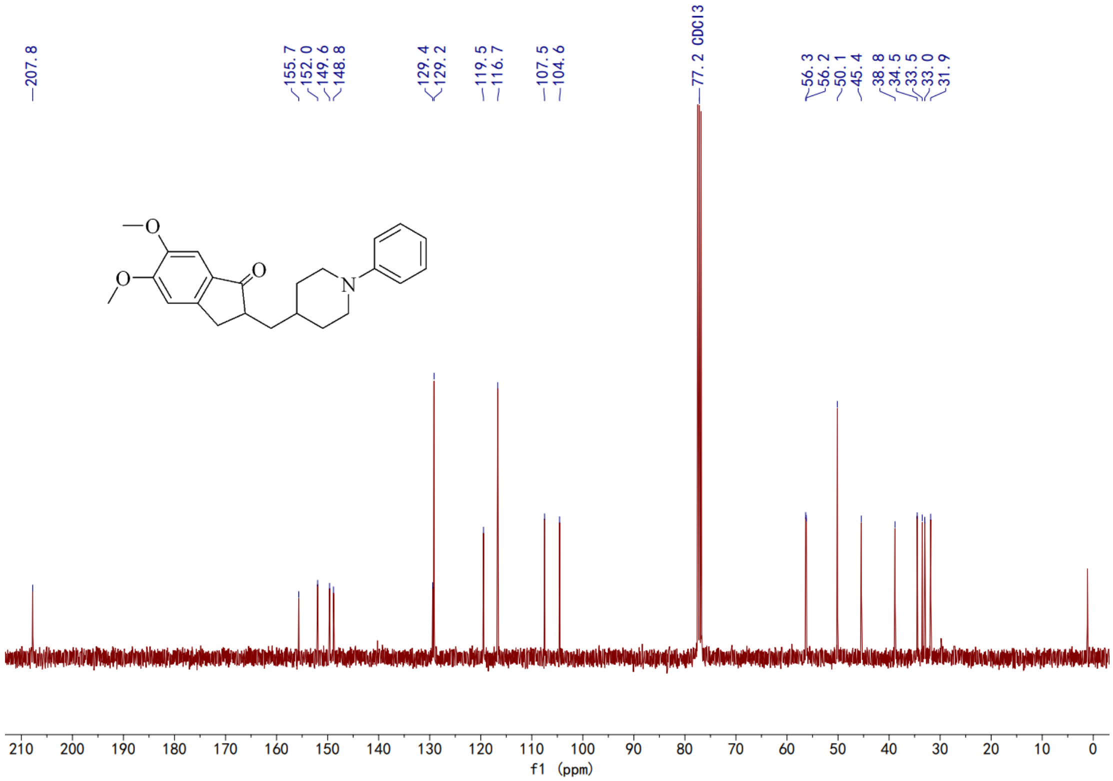

Figure 30. ^{13}C NMR spectrum of compound **14** ($CDCl_3$)

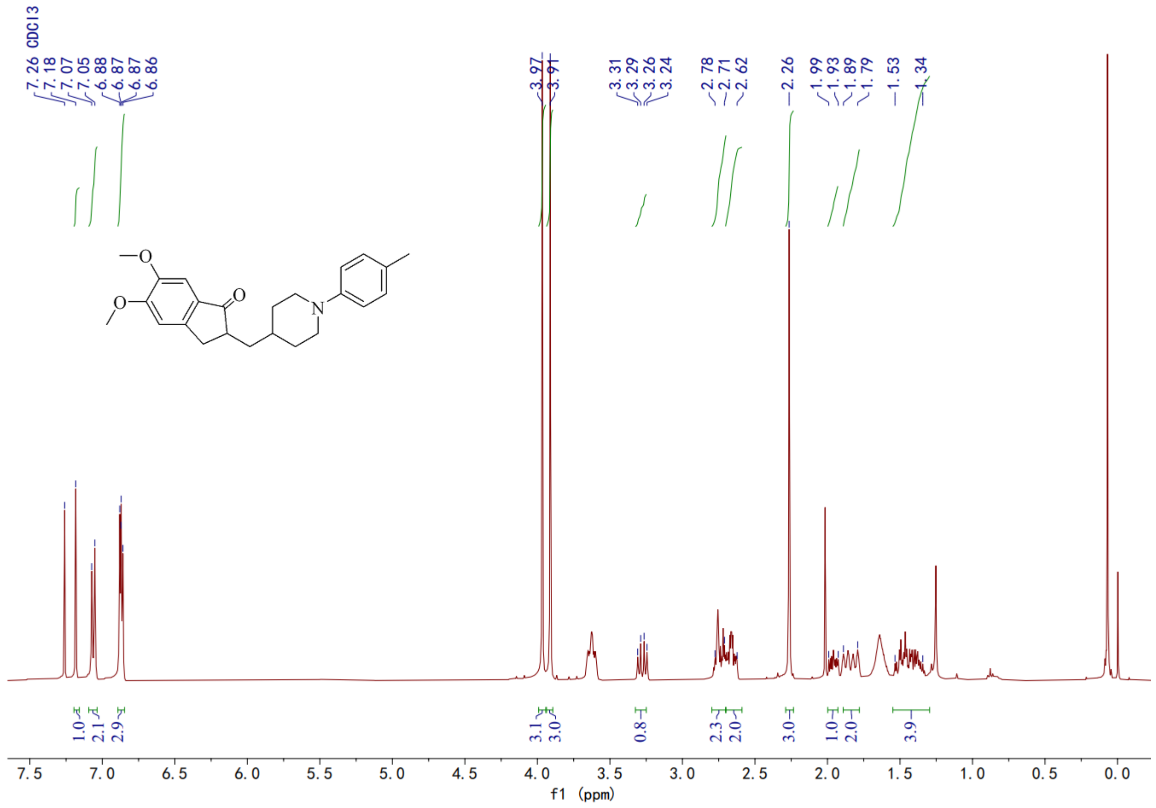

Figure 31. ^{1}H NMR spectrum of compound **15** ($CDCl_3$)

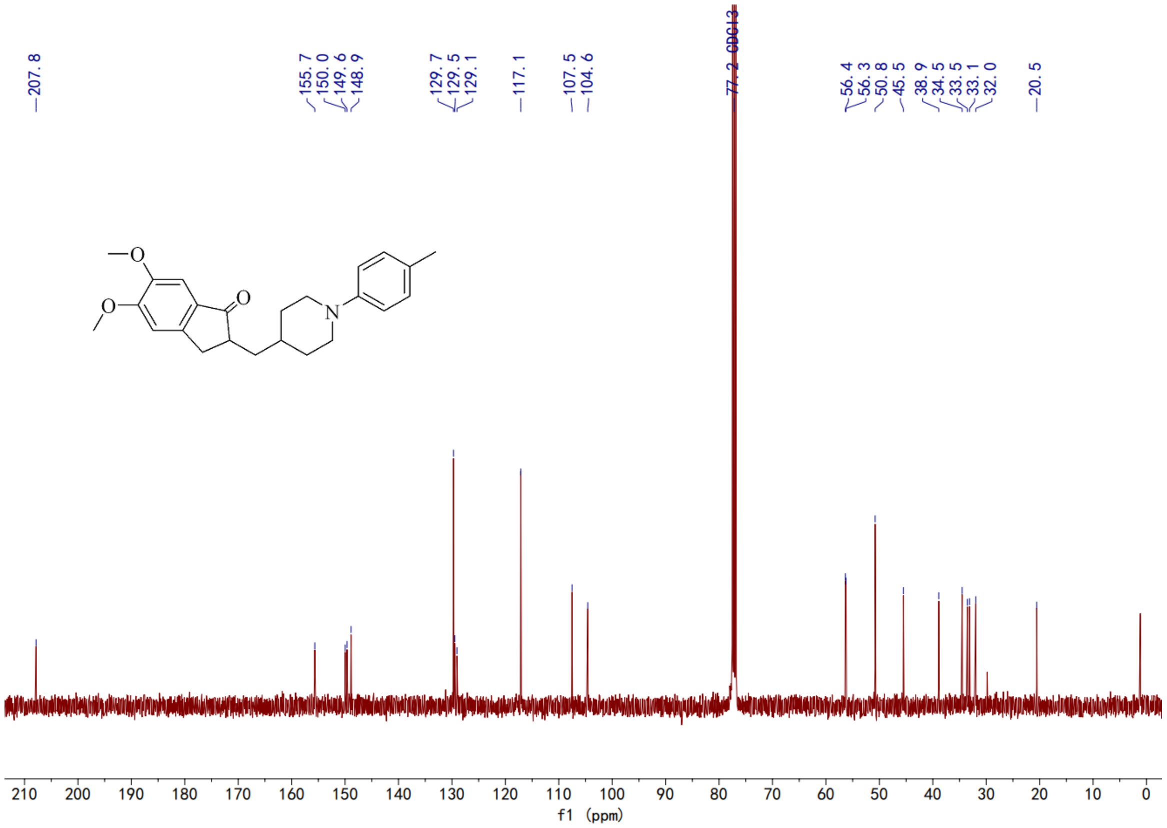

Figure 32. ^{13}C NMR spectrum of compound **15** ($CDCl_3$)

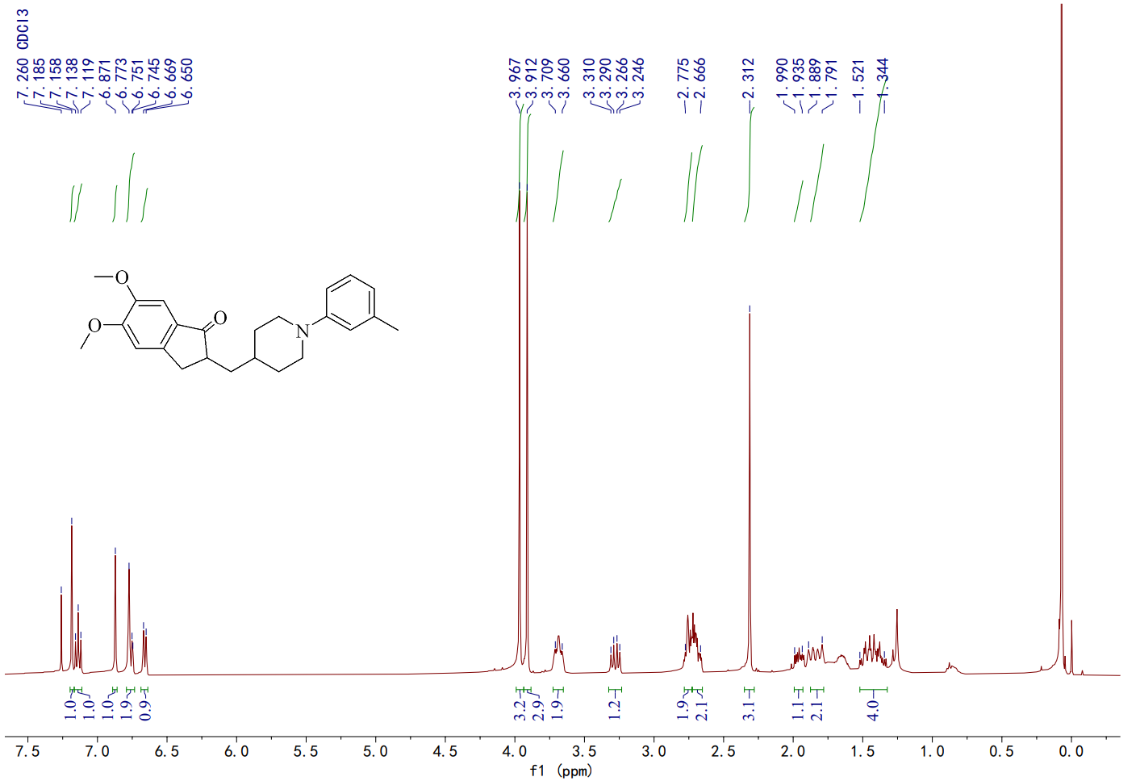

Figure 33. ^{1}H NMR spectrum of compound **16** ($CDCl_3$)

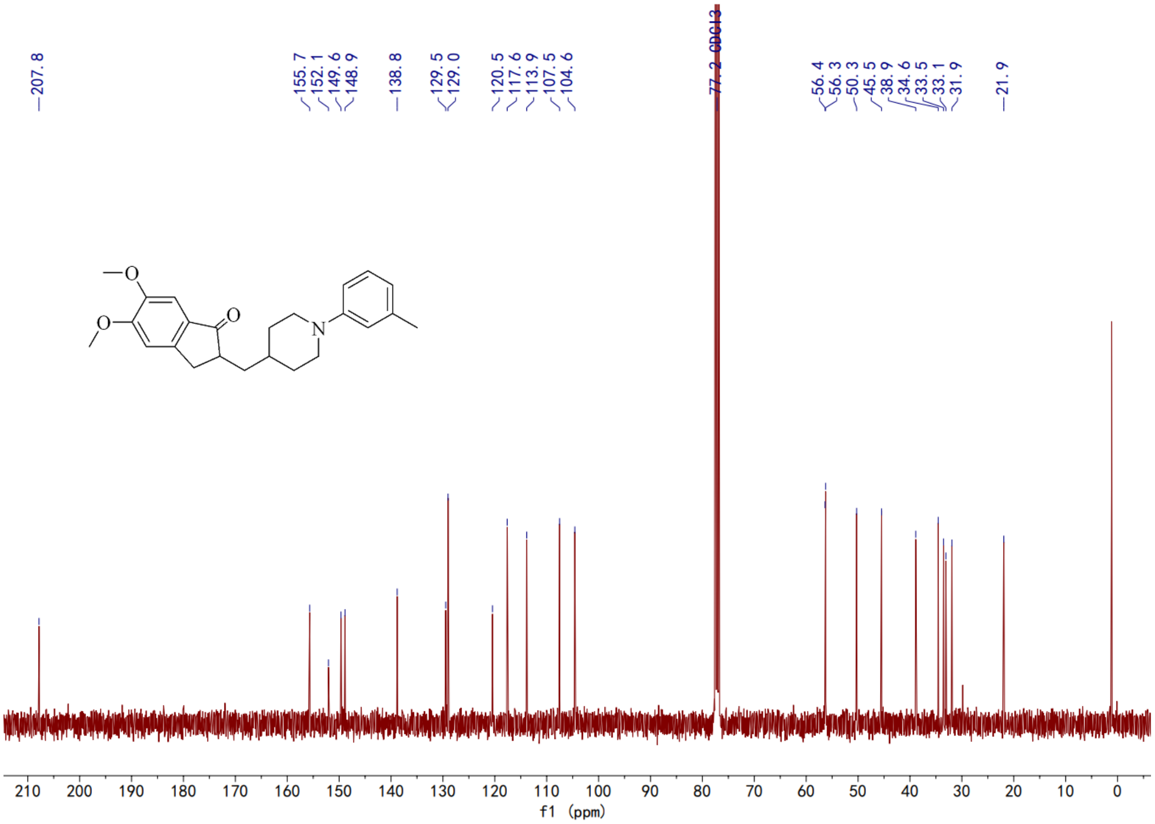

Figure 34. ^{13}C NMR spectrum of compound **16** ($CDCl_3$)

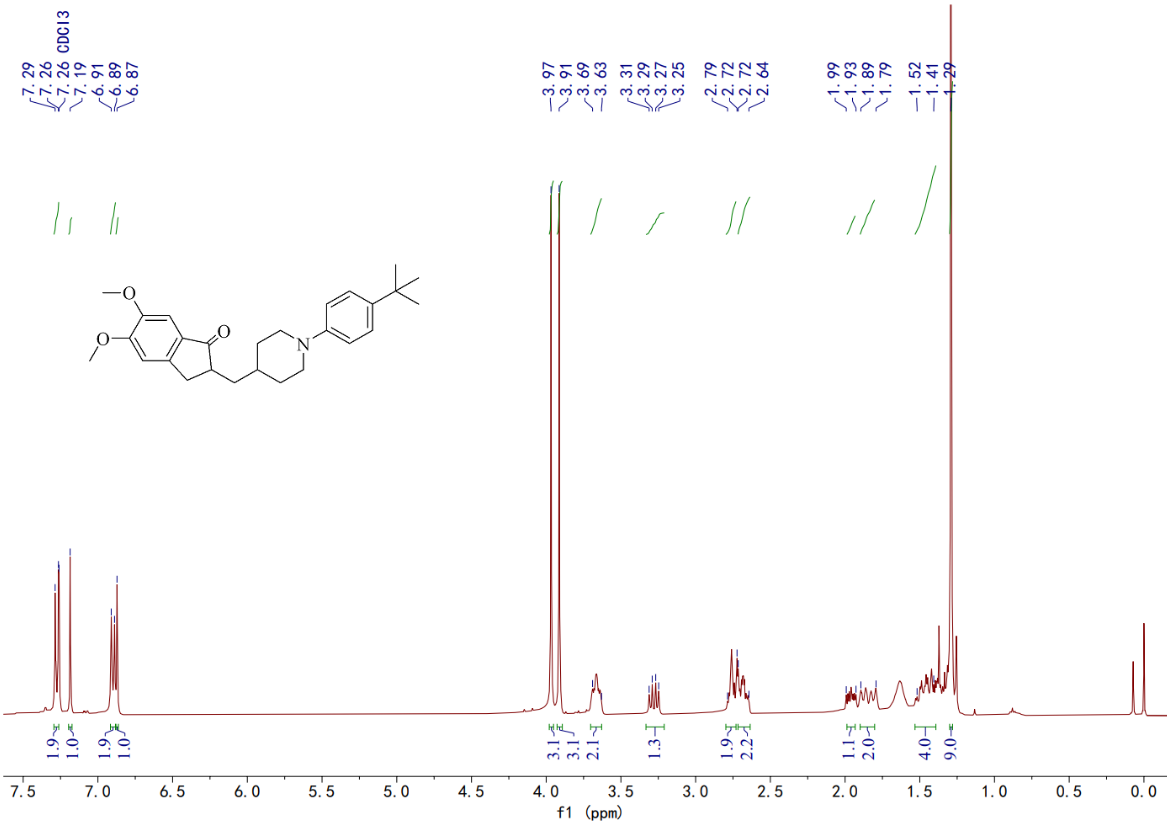

Figure 35. ^{1}H NMR spectrum of compound **17** ($CDCl_3$)

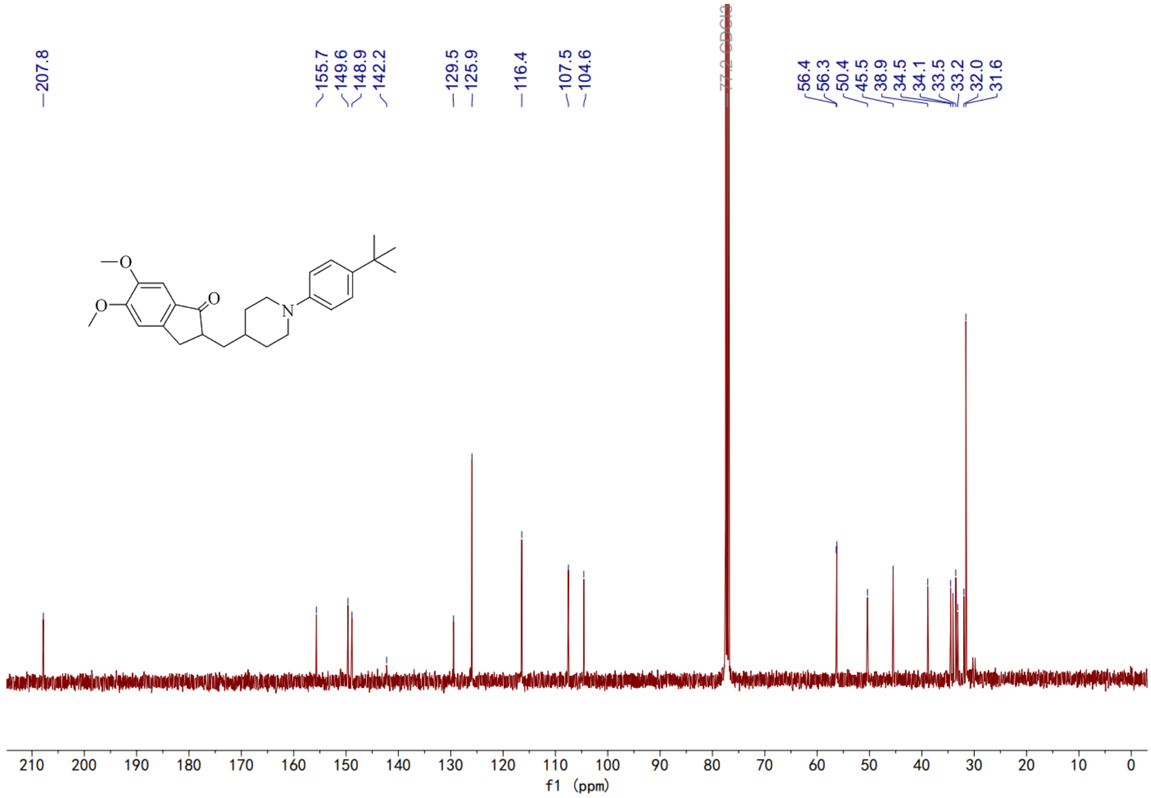

Figure 36. ^{13}C NMR spectrum of compound **17** ($CDCl_3$)

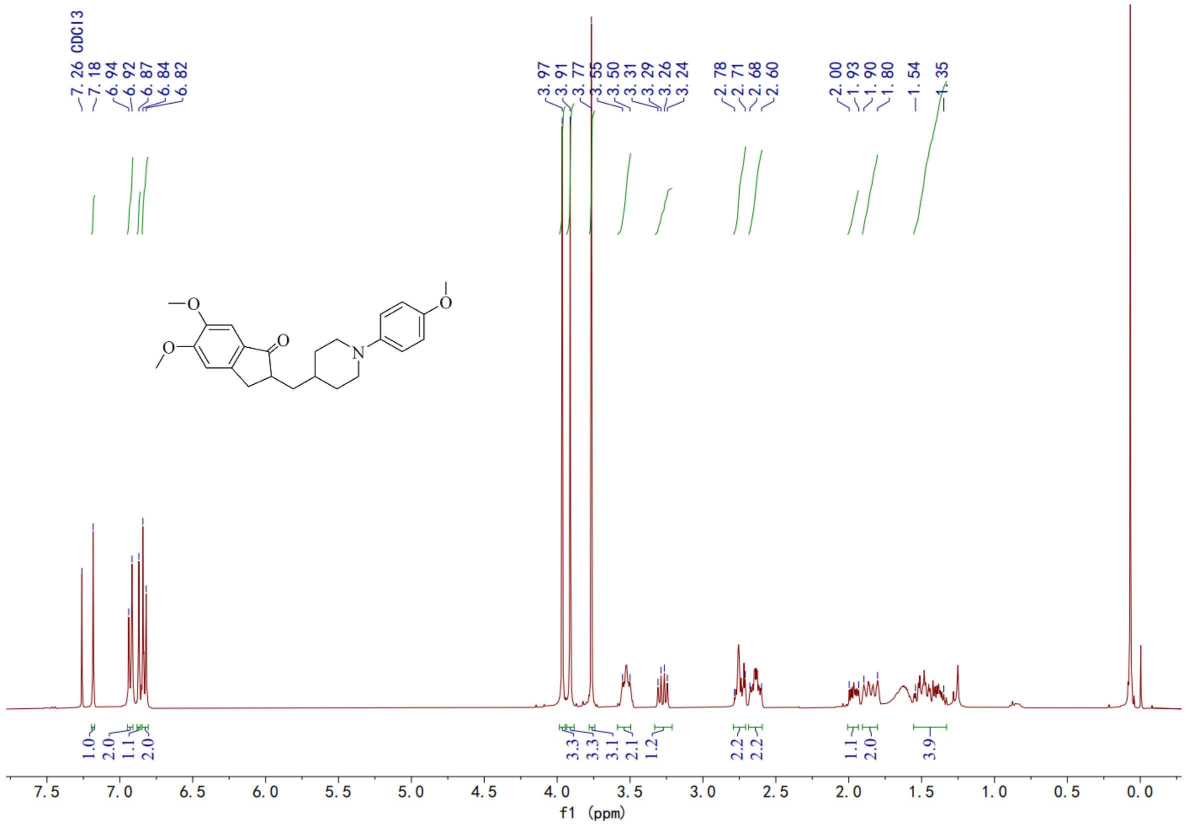

Figure 37. ^{1}H NMR spectrum of compound **18** ($CDCl_3$)

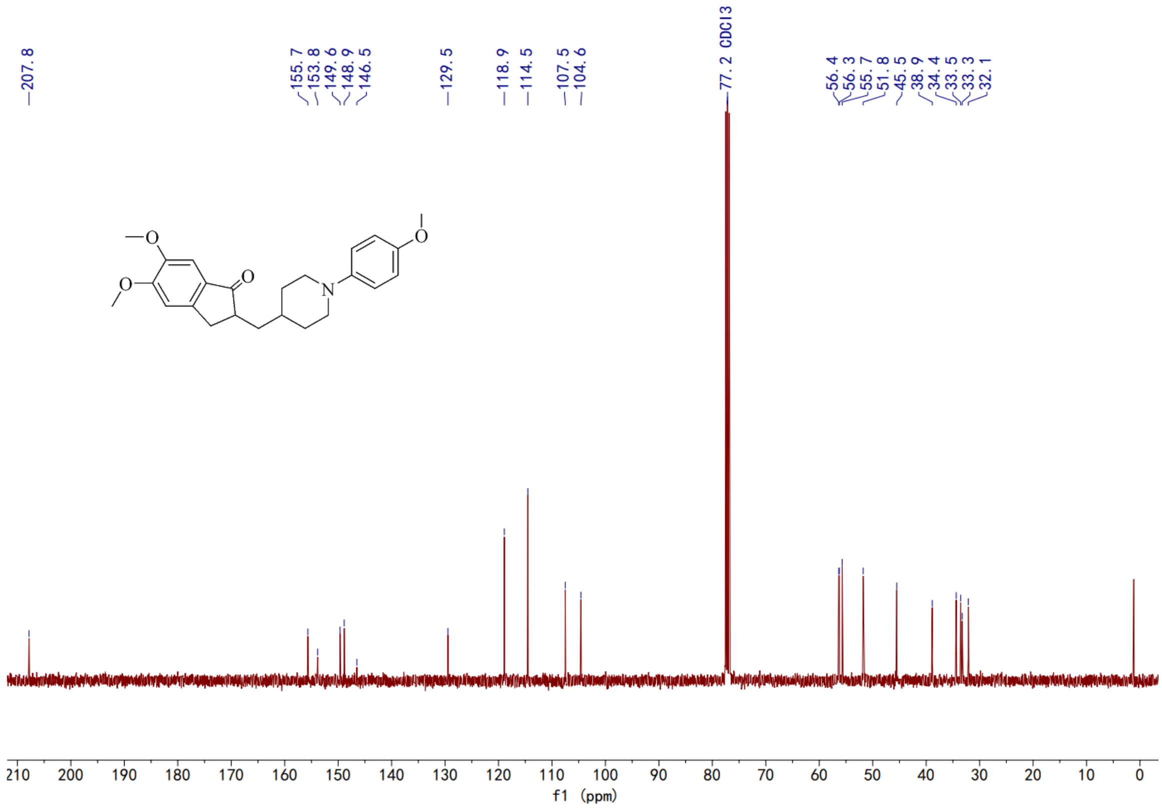

Figure 38. ^{13}C NMR spectrum of compound **18** ($CDCl_3$)

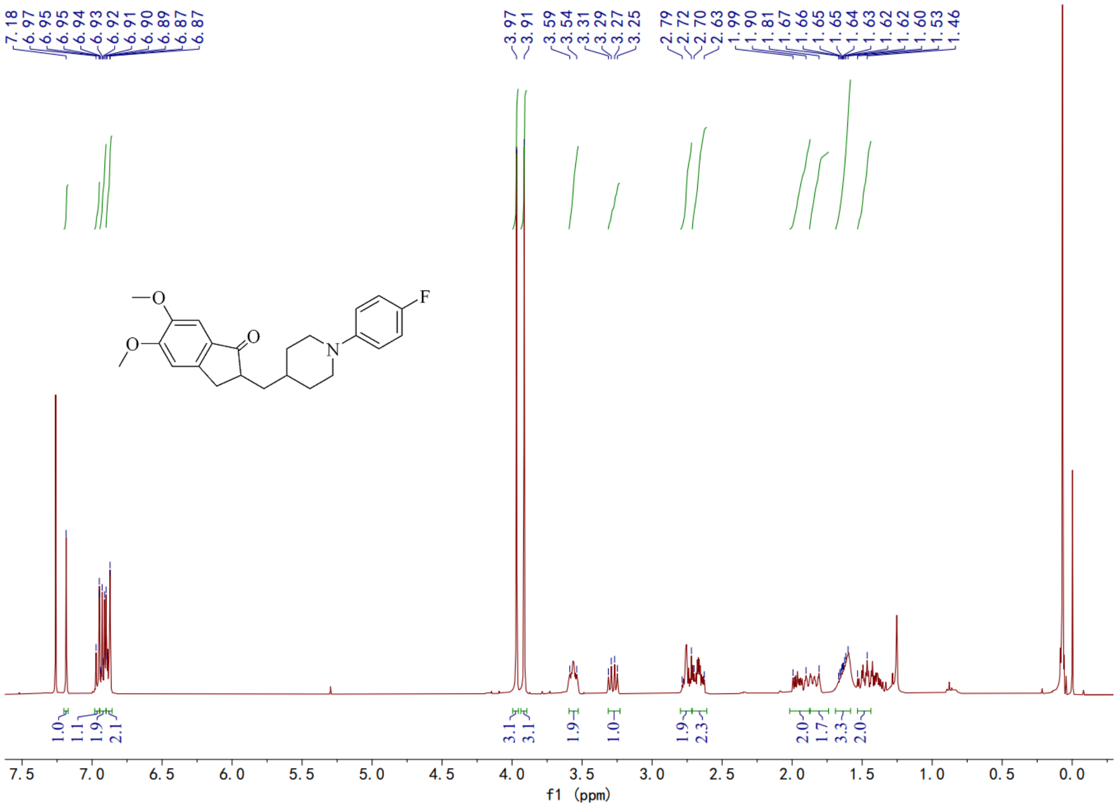

Figure 39. ^{1}H NMR spectrum of compound **19** ($CDCl_3$)

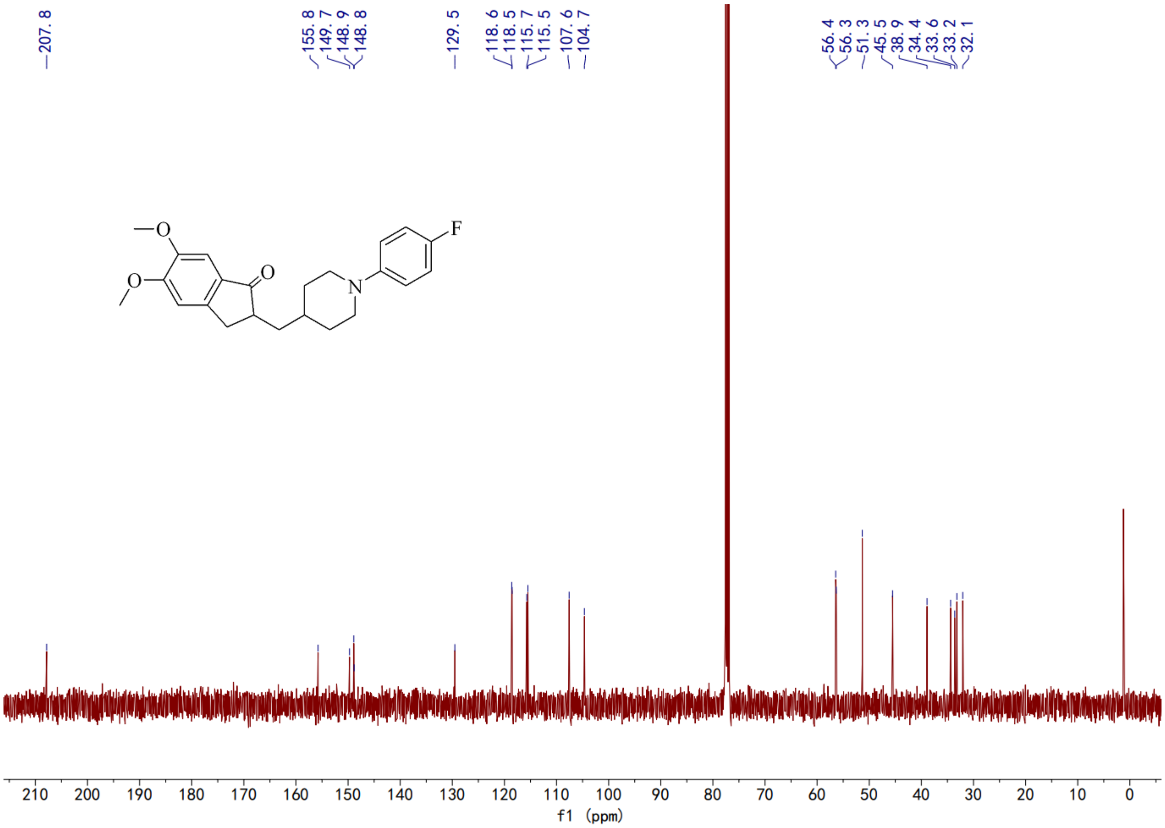

Figure 40. ^{13}C NMR spectrum of compound **19** ($CDCl_3$)

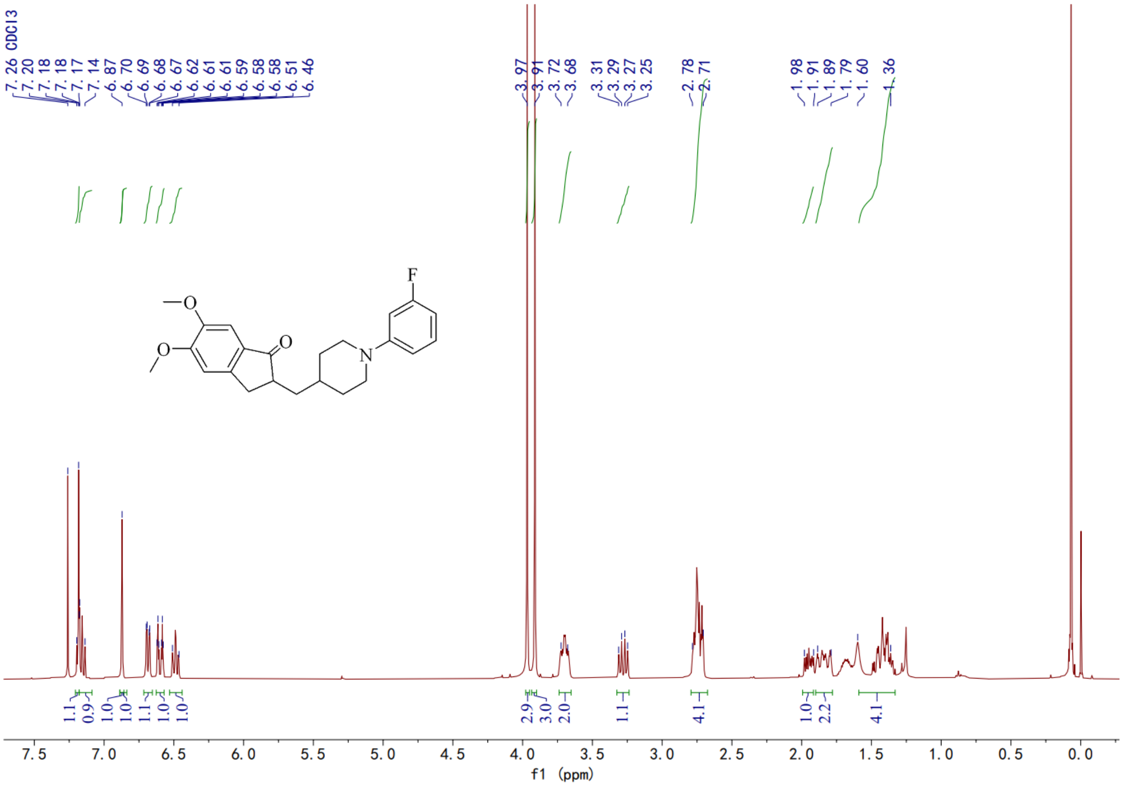

Figure 41. ^{1}H NMR spectrum of compound **20** ($CDCl_3$)

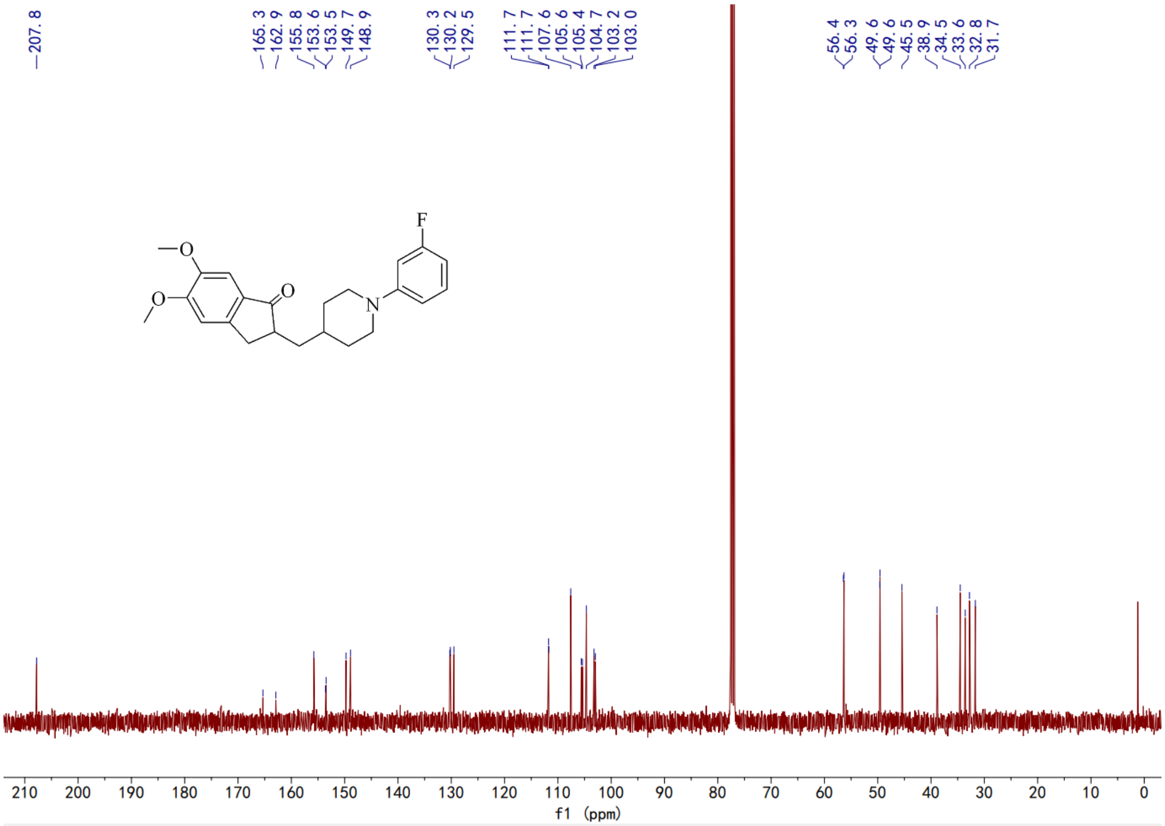

Figure 42. ^{13}C NMR spectrum of compound **20** ($CDCl_3$)

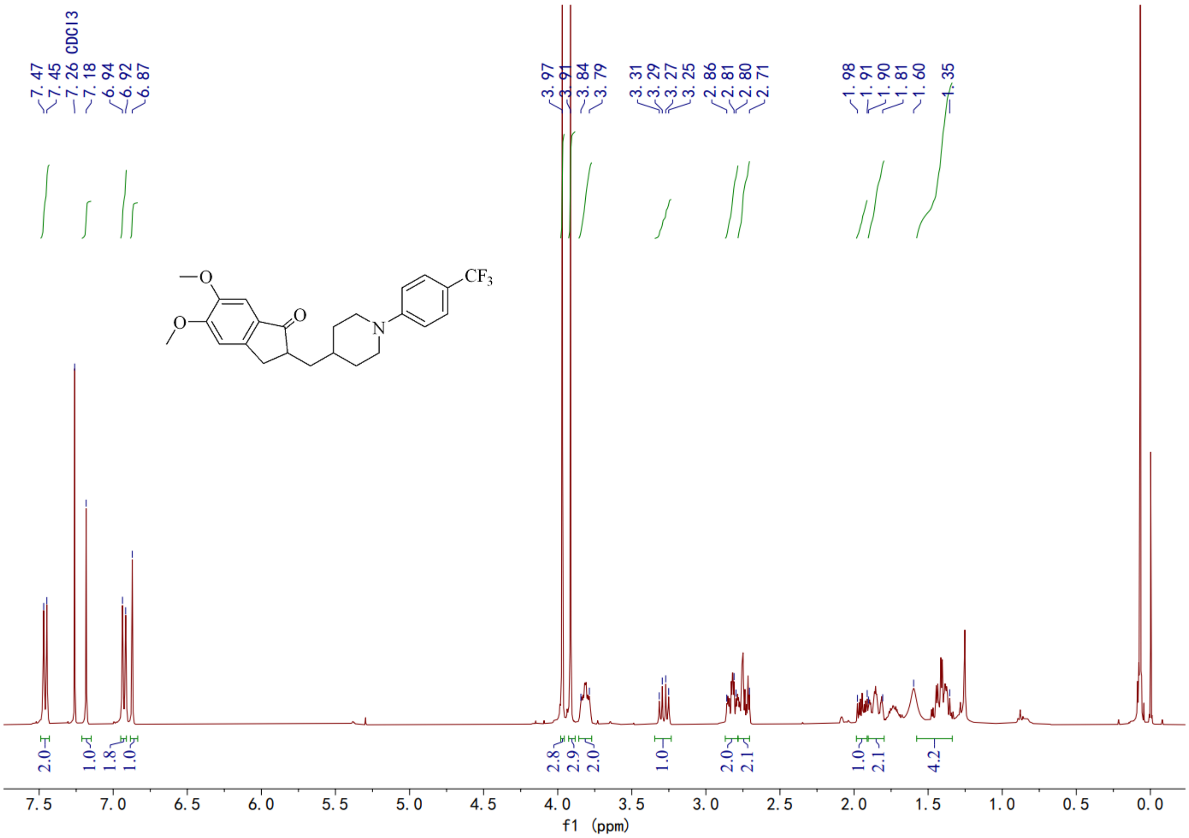

Figure 43. ^{1}H NMR spectrum of compound **21** ($CDCl_3$)

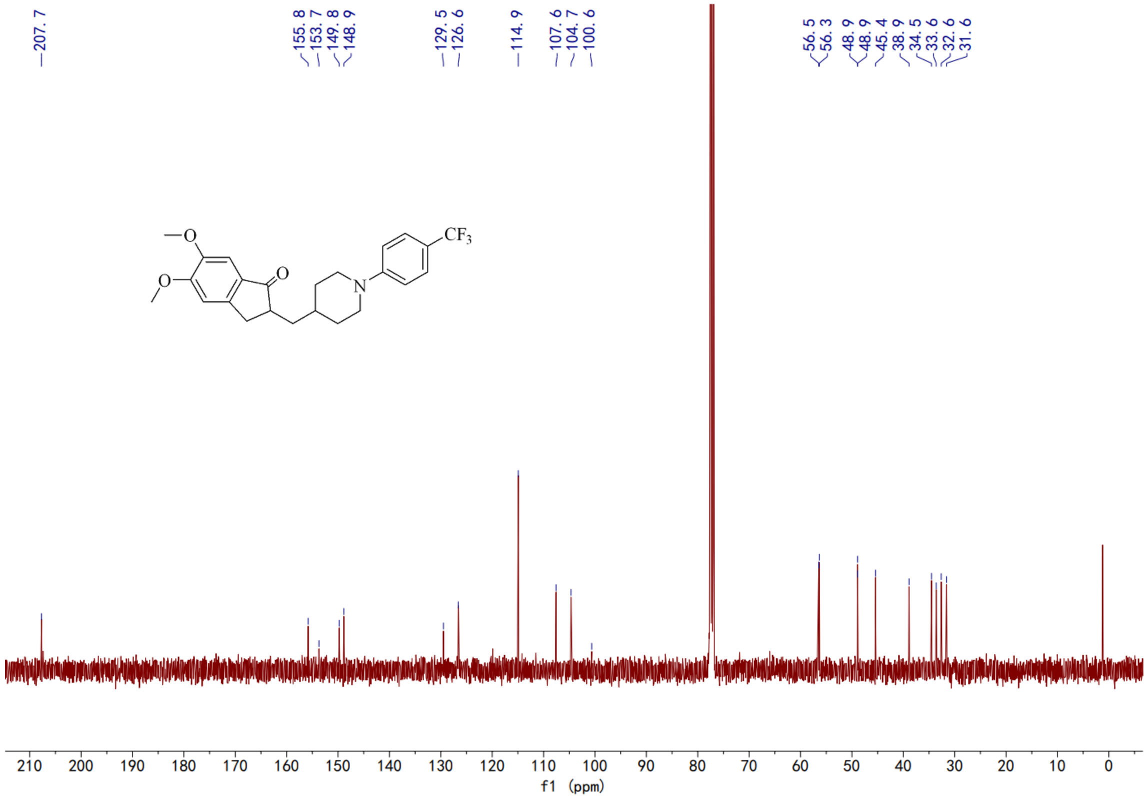

Figure 44. ^{13}C NMR spectrum of compound **21** ($CDCl_3$)

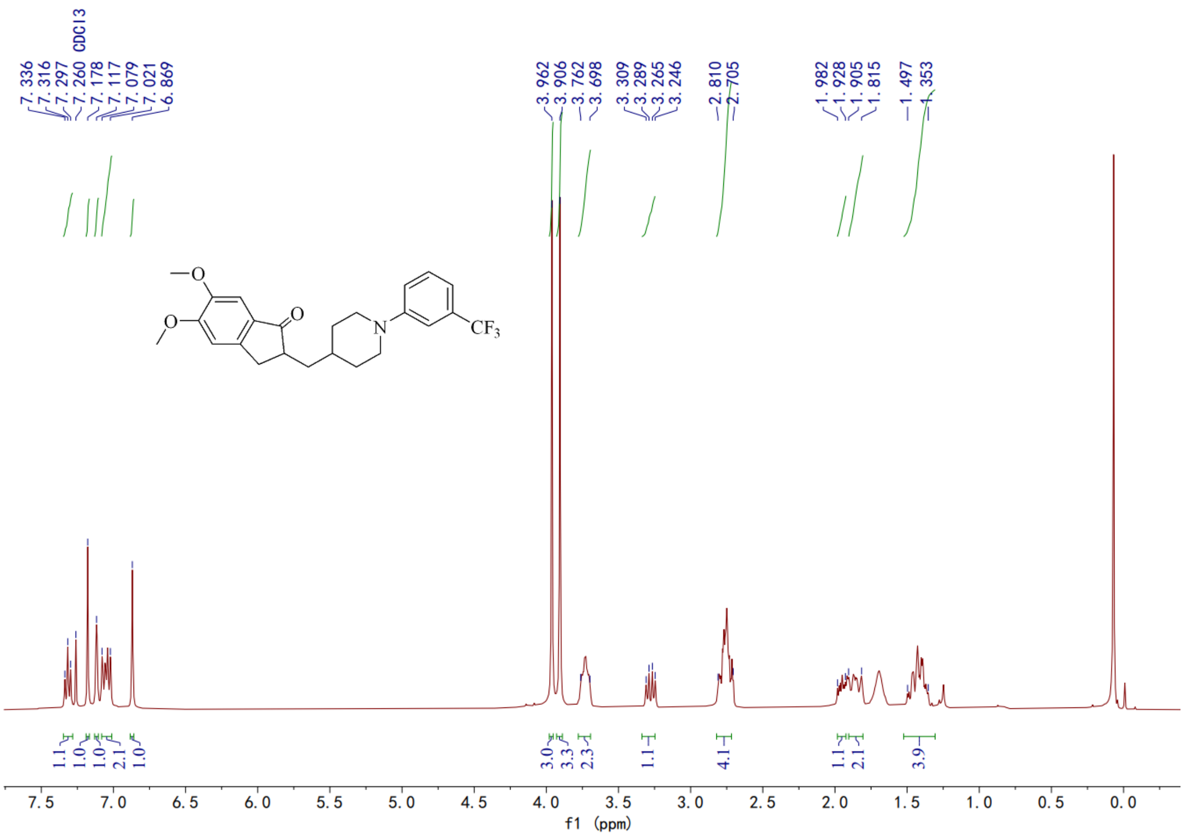

Figure 45. ^{1}H NMR spectrum of compound **22** ($CDCl_3$)

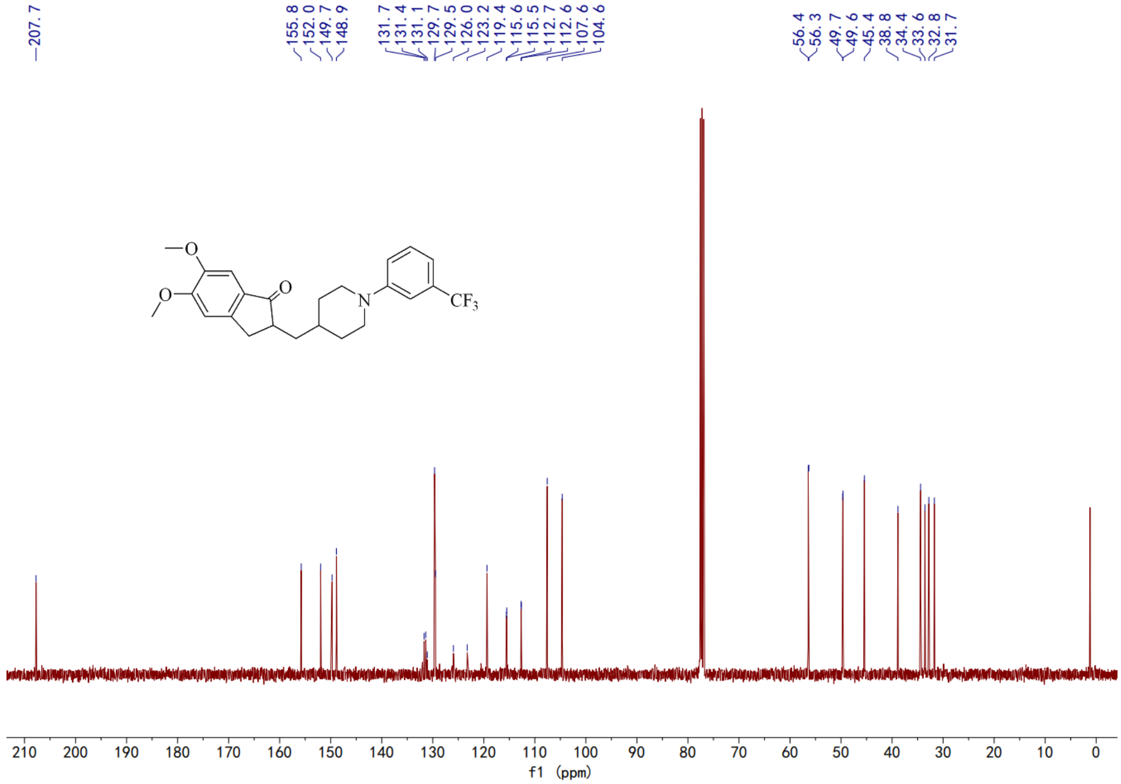

Figure 46. ^{13}C NMR spectrum of compound **22** ($CDCl_3$)

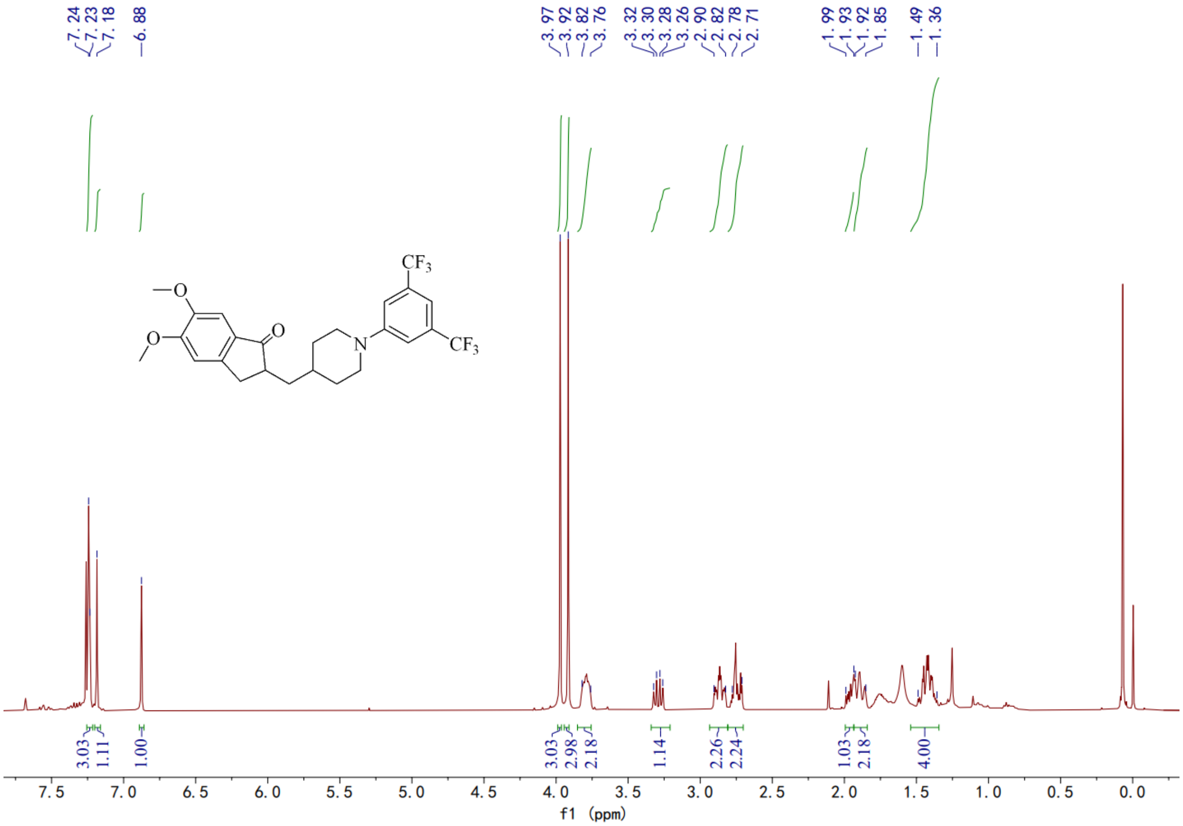

Figure 47. ^{1}H NMR spectrum of compound **23** ($CDCl_3$)

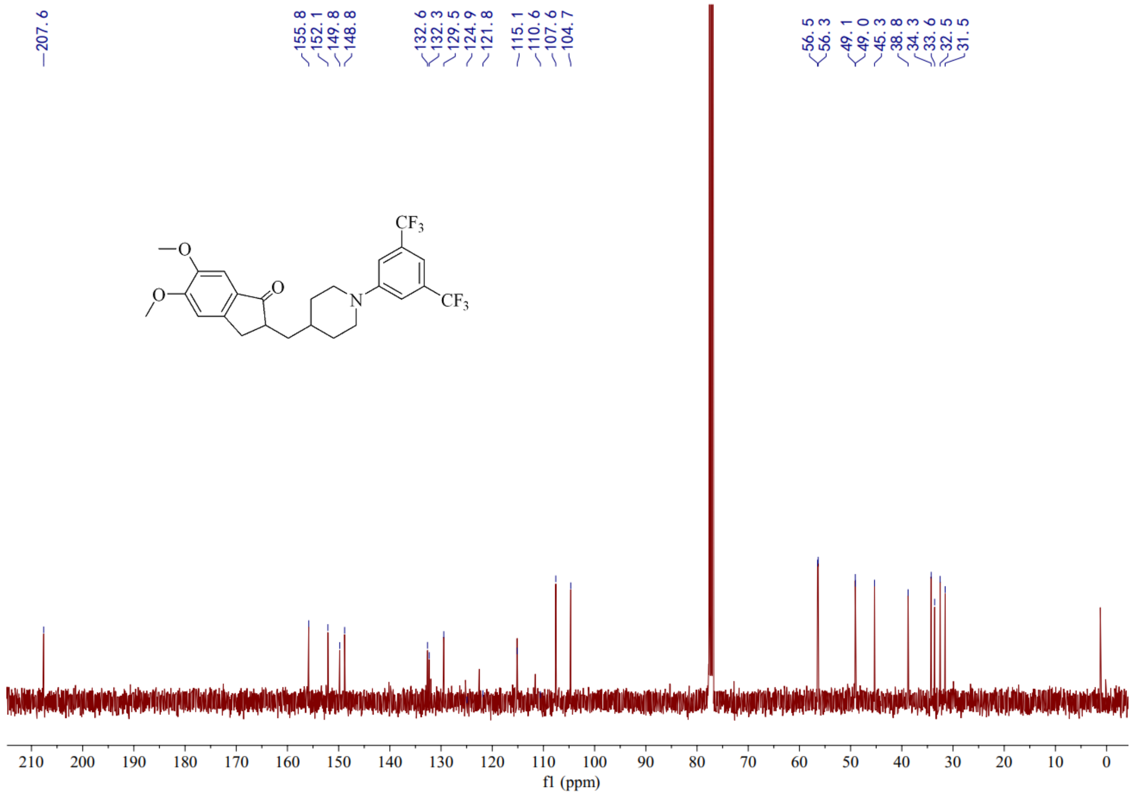

Figure 48. ^{13}C NMR spectrum of compound **23** ($CDCl_3$)

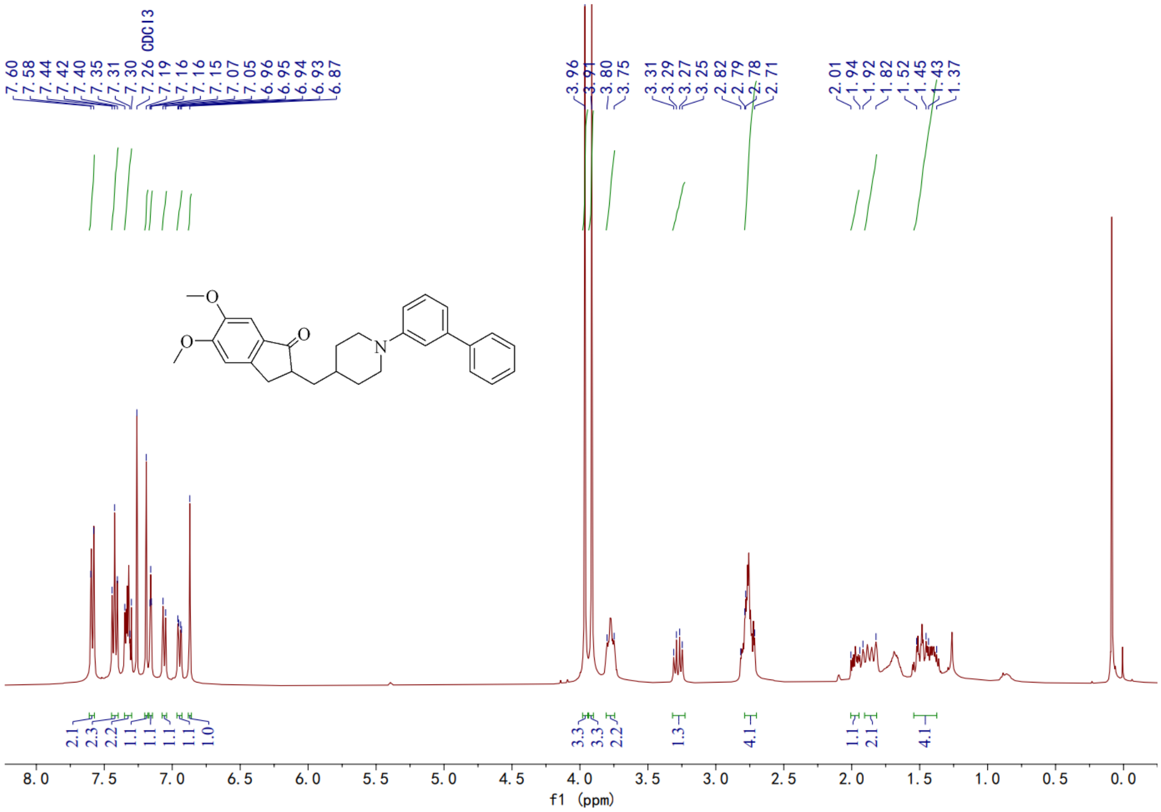

Figure 49. ^{1}H NMR spectrum of compound **24** ($CDCl_3$)

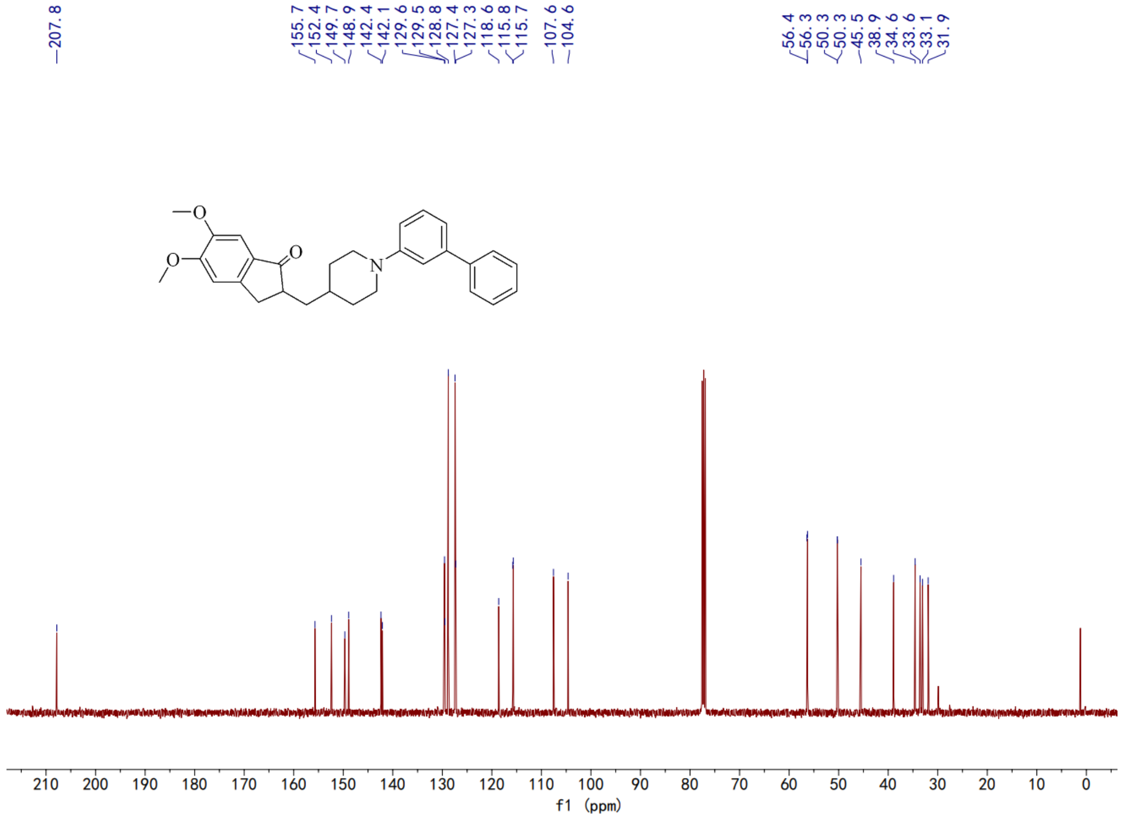

Figure 50. ^{13}C NMR spectrum of compound **24** ($CDCl_3$)

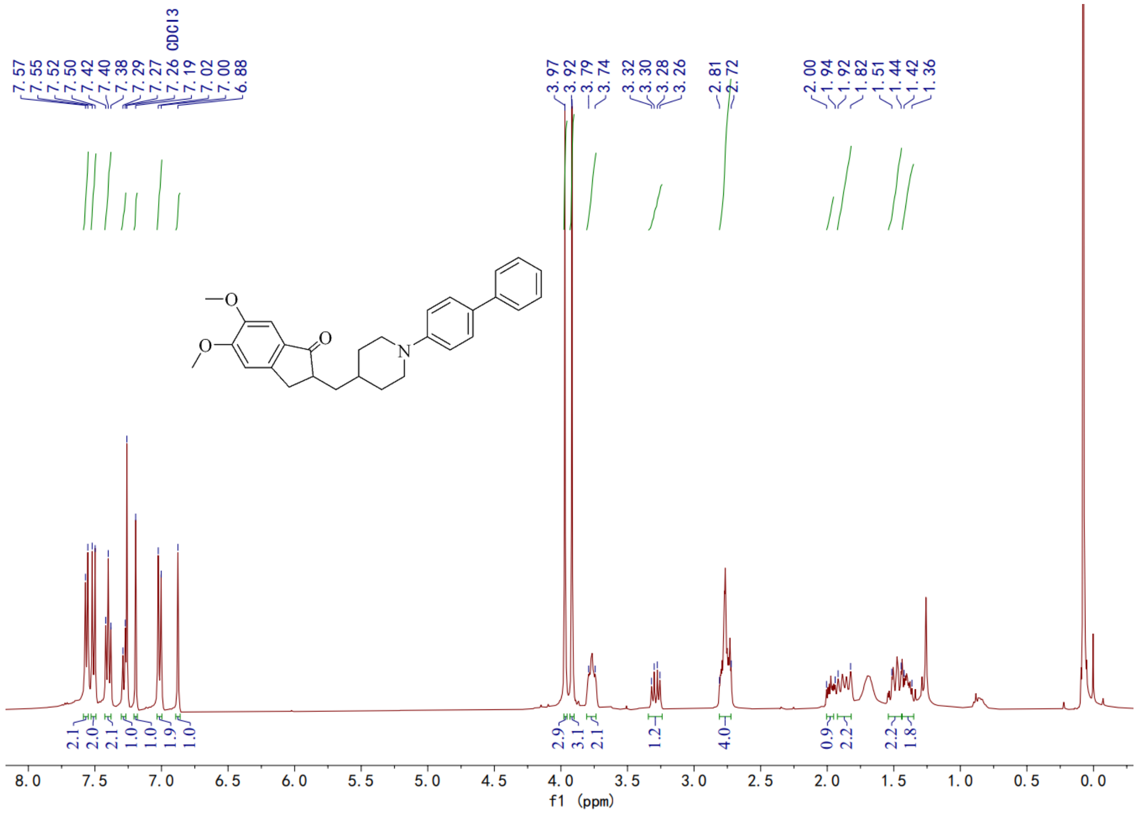

Figure 51. ^{1}H NMR spectrum of compound **25** ($CDCl_3$)

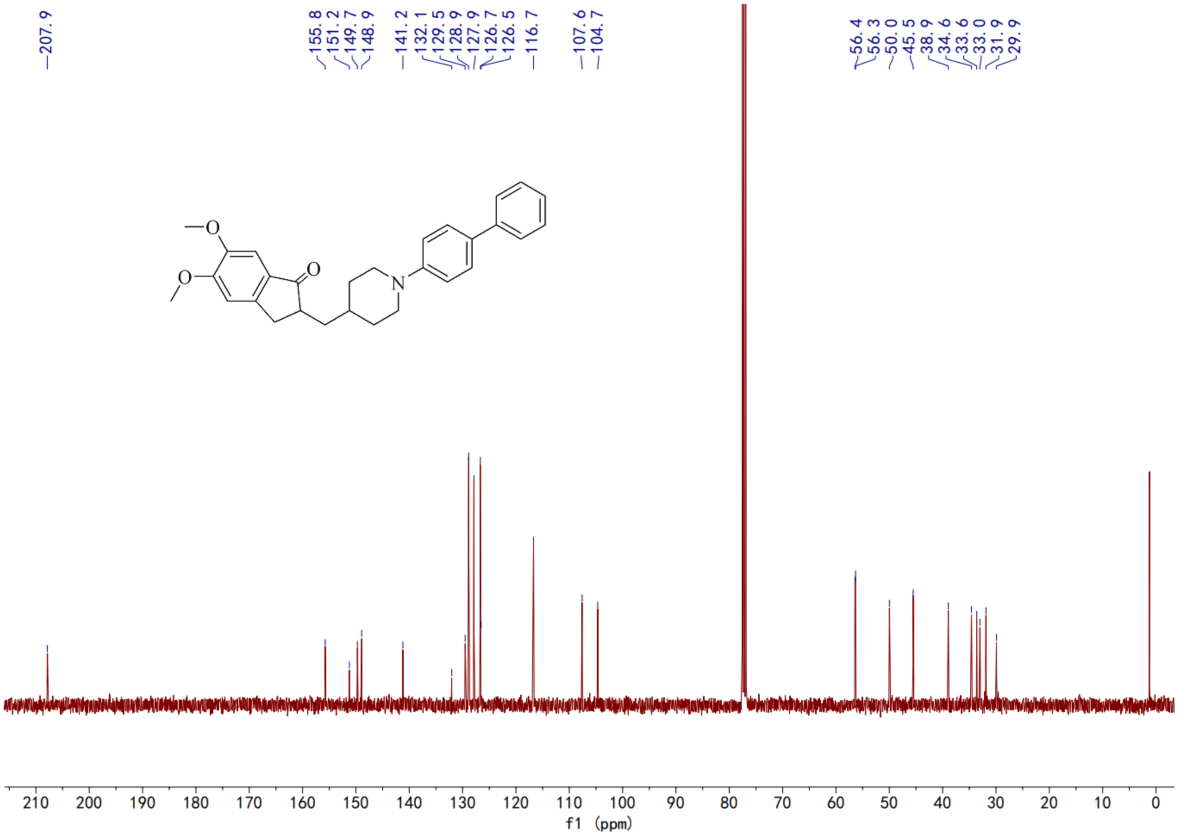

Figure 52. ^{13}C NMR spectrum of compound **25** ($CDCl_3$)

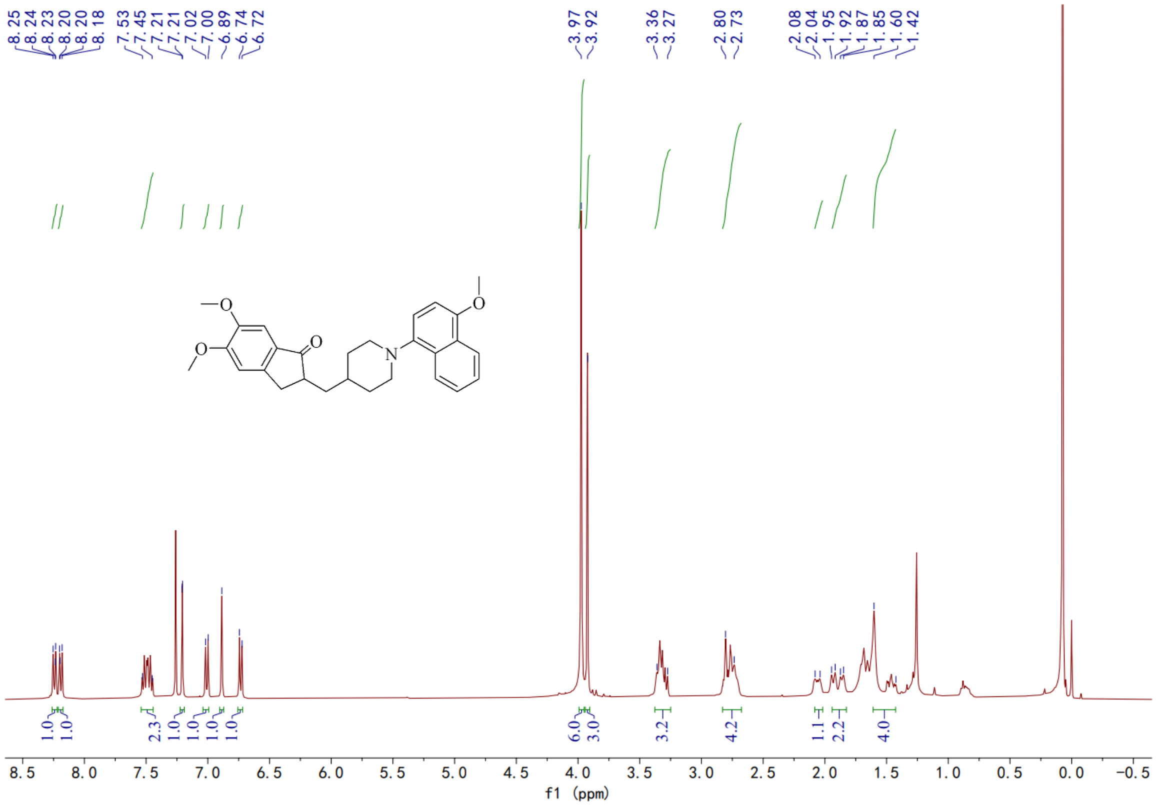

Figure 53. ^{1}H NMR spectrum of compound **26** ($CDCl_3$)

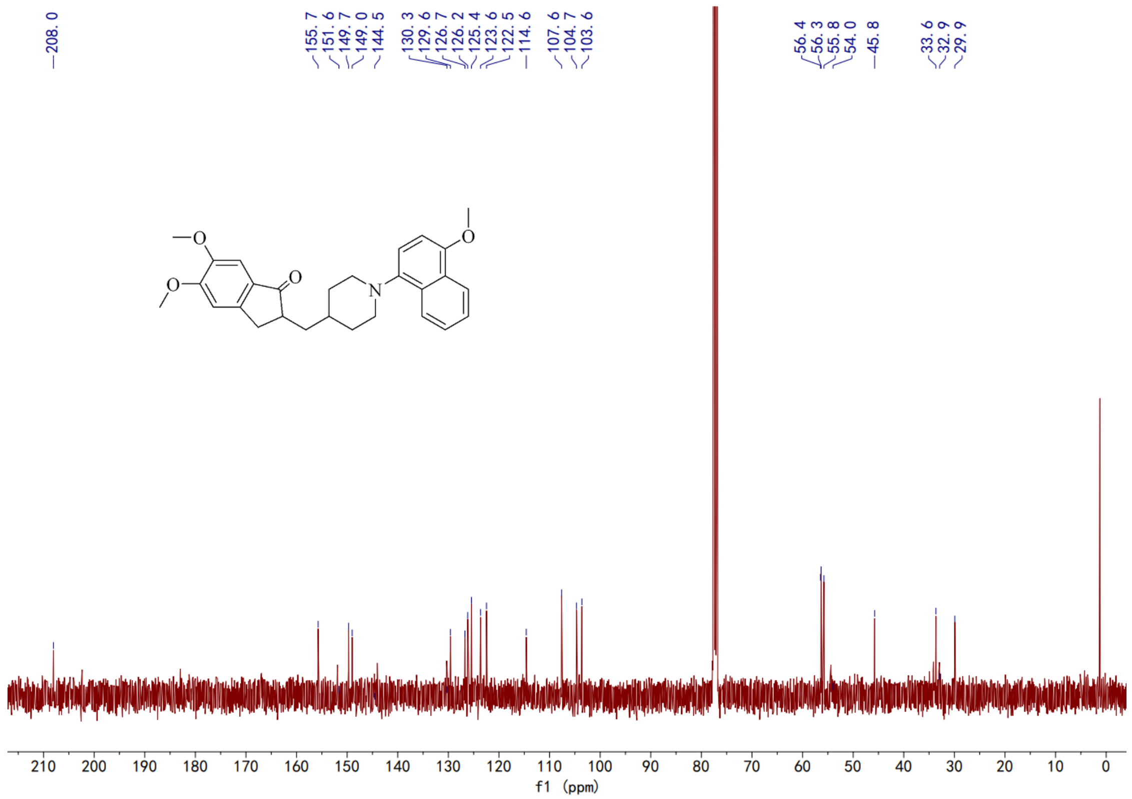

Figure 54. ^{13}C NMR spectrum of compound **26** ($CDCl_3$)
